# iCar-PseCp: identify carbonylation sites in proteins by Monte Carlo sampling and incorporating sequence coupled effects into general PseAAC

**DOI:** 10.18632/oncotarget.9148

**Published:** 2016-05-03

**Authors:** Jianhua Jia, Zi Liu, Xuan Xiao, Bingxiang Liu, Kuo-Chen Chou

**Affiliations:** ^1^ Computer Department, Jing-De-Zhen Ceramic Institute, Jing-De-Zhen 333403 China; ^2^ Gordon Life Science Institute, Boston, MA 02478, USA; ^3^ School of Computer Science and Engineering, Nanjing University of Science and Technology, Nanjing, 210094, China; ^4^ Center of Excellence in Genomic Medicine Research (CEGMR), King Abdulaziz University, Jeddah 21589, Saudi Arabia

**Keywords:** carbonylation, sequence-coupling model, PseAAC, Monte Carlo sampling, random forest algorithm

## Abstract

Carbonylation is a posttranslational modification (PTM or PTLM), where a carbonyl group is added to lysine (K), proline (P), arginine (R), and threonine (T) residue of a protein molecule. Carbonylation plays an important role in orchestrating various biological processes but it is also associated with many diseases such as diabetes, chronic lung disease, Parkinson's disease, Alzheimer's disease, chronic renal failure, and sepsis. Therefore, from the angles of both basic research and drug development, we are facing a challenging problem: for an uncharacterized protein sequence containing many residues of K, P, R, or T, which ones can be carbonylated, and which ones cannot? To address this problem, we have developed a predictor called iCar-PseCp by incorporating the sequence-coupled information into the general pseudo amino acid composition, and balancing out skewed training dataset by Monte Carlo sampling to expand positive subset. Rigorous target cross-validations on a same set of carbonylation-known proteins indicated that the new predictor remarkably outperformed its existing counterparts. For the convenience of most experimental scientists, a user-friendly web-server for iCar-PseCp has been established at http://www.jci-bioinfo.cn/iCar-PseCp, by which users can easily obtain their desired results without the need to go through the complicated mathematical equations involved. It has not escaped our notice that the formulation and approach presented here can also be used to analyze many other problems in computational proteomics.

## INTRODUCTION

Cancer and many other major diseases are often caused by varieties of subtle modifications in biological sequences, typically by various types of post-translational modification (PTM or PTLM) in protein [1, 2], post-replication modification (PTRM) in DNA [3] and post-transcription modification (PTCM) in RNA [4]. In order to reveal the pathological mechanisms of these diseases and find new and revolutionary strategies to treat them, considerable efforts have been made in order for identifying the possible modified sites in proteins (see, e.g., [5–13]), DNA [14, 15], and RNA sequences [16, 17]. For a systematic introduction about this, see two recent review articles [13, 15].

*In vivo*, PTM is one of the most efficient biological mechanisms for regulating physiology as well as for expanding the genetic code. But when body's well-designed proteolysis or other repair systems are overwhelmed by excess reactive oxygen species (ROS) [18], the oxidative stress may occur [18], weakening the damage-repairing ability. This may also bring about varieties of PTMs on proteins, including nitration, carbonylation, sulfhydration and glutathionylation [19]. Among these PTMs, the protein carbonylation has been used as a biomarker for severe oxidative protein damage due to its relative early formation, stability, and irreversibility [20, 21]. Actually, protein carbonylation is an early stage of diseases induced by external oxidative stress, aging and obesity [22, 23]. It may cause numerous major human diseases, including Alzheimer's disease, diabetes, Parkinson's disease, chronic renal failure, chronic lung disease, sepsis and so forth [24, 25]. Therefore, the information of carbonylation sites in proteins is indispensable not only for in-depth understanding many important biological processes but also for precisely aiming targets in developing effective drugs against the aforementioned diseases.

Mass spectrometry is one of the most common techniques to analyze the carbonyl level of a protein and determine its carbonylation sites [26, 27]. So far four types of amino acid residues have been found more prone to carbonylation; they are lysine (K), proline (P), arginine (R), and threonine (T) [24, 28–30]. But it would take much longer time and need more labors to utilize the conventional experimental techniques alone to determine the carbonylation sites in proteins [27, 31]. Facing the rapid growth of biological sequences, we are challenged to develop automated methods as a complimentary approach to experimental methods.

Actually, some investigators have made efforts to do so. Maisonneuve et al. [29], based on their spectrometry analysis, proposed some empirical rules to identify the hot spots of carbonylation. Recently, Lv et al. [32] and Xu et al. [33] developed two different bioinformatical tools to predict the protein carbonylation sites. These methods did have contribution in stimulating the development of this area. Since the topic's importance as well as the urgency of demanding more powerful high throughput tools in this area, further efforts aiming at prediction of protein carbonylation sites are definitely needed.

Here, we are to develop a new and more powerful predictor by (1) using the Monte Carlo sampling approach to optimize the training dataset, (2) incorporating the vectorized sequence-coupling model into the general PseAAC, and (3) installing the random forest (RF) algorithm to operate the prediction system.

As shown in many recent relevant papers [11, 12, 14, 17, 34–40], to establish a biological sequence-based statistical predictor that not only can be easily used by most experimental scientists to get their desired results but also can inspirely stimulate theoretical scientists to create various other prediction methods, we should observe the Chou's 5-step rules or guidelines [41]: (1) benchmark dataset preparation; (2) mathematical representation of biological sequence samples; (3) calculation algorithm; (4) cross-validation; (5) web-server establishment. Below, let us to address the five guidelines one-by-one. To match the rubric style of the Oncotarget journal, however, the order in addressing them may be changed.

## RESULTS AND DISCUSSION

### A novel web-server predictor and its user guide

A new and more powerful predictor, called iCar-PseCp, has been established for predicting the protein carbonylation sites. Moreover, to maximize users' convenience, the point-to-point instructions are given below.

**Figure 1:**
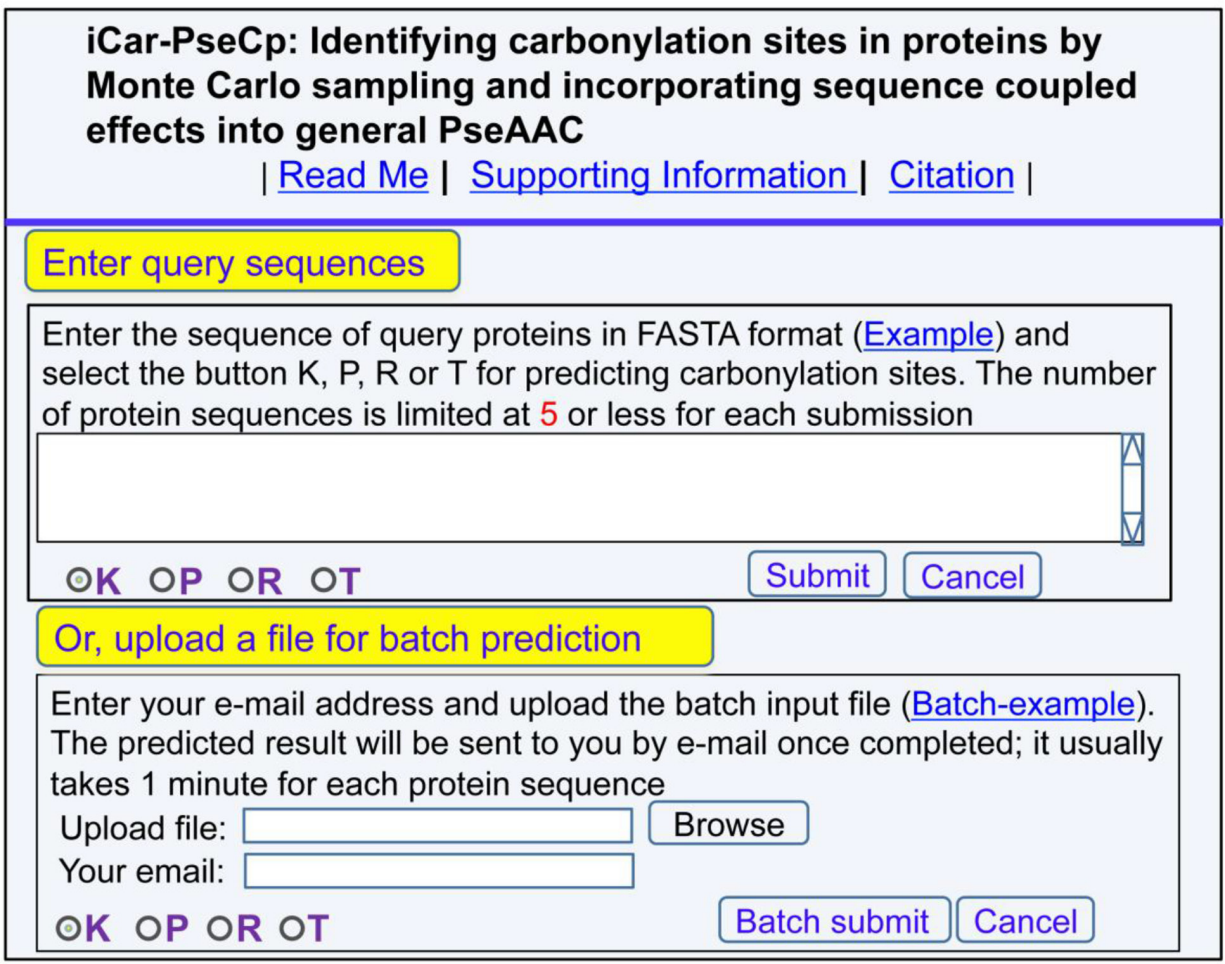
A semi-screenshot of the top-page for the web-server iCar-PseCp at http://www.jci-bioinfo.cn/iCar-PseCp

(1) Click the web-server at http://www.jci-bioinfo.cn/iCar-PseCp, the top page of the iCar-PseCp will be prompted on your computer screen (Figure 1).
(2) In the input box (Figure 1), enter your query protein sequences, which can be done by either typing or copying/pasting manner. The entered query protein sequences should be in the FASTA format. Not familiar with FASTA? Just click the button of Example.
(3) You can see the prediction results by clicking the Submit button. If you use the Sequence_K in the Example window as the input and check on the K button, after 15 seconds or so since your submitting, you will see the following on your screen: Sequence_K contains 9 K residues, of which 5 are predicted to be of carbonylation site and they are at the sequence positions 2, 14, 41, 68 and 95. If you use the Sequence_P as the input and check on the P button, you will see: Sequence_P contains 10 P residues, of which 5 are of carbonylation site and at positions 95, 122, 142, 145, and 149. If you use the Sequence_R as the input and check on the R button, you will see: Sequence_R contains 8 R residues, of which 3 are of carbonylation site and at the positions 14, 41, and 75. If you use the Sequence T as the input and check on the T button, you will see: Sequence_T contains 7 T residues, of which 1 is of carbonylation site and at the positions 14. Compared with experimental observations, the above (9 + 10 + 8 + 7) = 34 predicted results contain no false positive result $\left(N_{-}^{+}=0\right)$ but 5 false positive results $\left(N_{+}^{-}=5\right)$, which are the 2nd and 13th K residues in sequence_K, the 142th and 145th P residues in sequence_P, and the 75th R residue in sequence_R. In other words, the total number of carbonylation sites involved in the above predictions is *N^+^ =* 3 + 3 + 1 + 1 = 8, while the total number of non-carbonylation sites investigated is *N*^−^ *=* 6 + 7 + 6 + 6 = 25. Substituting these data into Eq.9, we have Sn = 100%, Sp = 80.00% and Acc = 84.80%, and MCC = 0.7018, quite consistent with the rates reported in Table 1 via the rigorous cross validation on the 250 benchmark proteins.
(4) If you have a lot of query protein sequences and need a lot of computational time, you can choose to use the batch prediction. To do so, just use the Browse button to select the desired file (in FASTA format of course) and follow the online instruction.
(5) The benchmark dataset used in this study is available by clicking the button of Supporting Information on the top of Figure 1.
(6) To see the key papers used to develop this server, just click on the button of Citation.

**Table 1:** A comparison of the proposed predictor with the existing methods based on the 10-fold cross-validation on the same 250 carbonylated proteins

Predictor	Metrics and graph	Type of carbonylation			
		K	P	R	T
PTMPred^a^	Acc (%)^d^	88.59	82.93	86.64	88.39
CarSpred^b^		87.22	82.93	86.22	86.61
iCar-PseCp^c^		84.43	86.79	84.23	86.17
PTMPred^a^	MCC^d^	0.1892	0.2573	0.1878	0.2186
CarSpred^b^		0.2268	0.2331	0.2245	0.2040
iCar-PseCp^c^		0.5906	0.6006	0.6076	0.6185
PTMPred^a^	Sn (%)^d^	23.45	21.43	20.02	22.38
CarSpred^b^		23.17	25.34	25.47	21.39
iCar-PseCp^c^		45.18	48.20	46.67	50.68
PTMPred^a^	Sp (%)^d^	92.99	93.20	90.99	91.36
CarSpred^b^		92.43	93.28	93.39	93.42
iCar-PseCp^c^		99.25	98.54	99.57	98.58
PTMPred^a^	AUC^e^	0.6858	0.6903	0.5981	0.6563
CarSpred^b^		0.6849	0.7163	0.7158	0.7134
iCar-PseCp^c^		0.8728	0.8484	0.8668	0.8603

a The predictor developed in [33], where ξ = 13; i.e. the sample length is 27.

b The predictor developed in [32], where the sample length was not fixed.

c The predictor proposed in this paper.

d See Eq.9 for the definition of metrics.

e The area under the curve of Figure.2; the greater the AUC value is, the better the corresponding predictor will be [52, 53].

### Result comparison and analysis

The success scores achieved by the iCar-PseCp predictor via the 10-fold target cross validation for K-, P-, R-, and T-type carbonylation are shown in Table 1. Meanwhile, the corresponding rates by PTMPred [33] and CarSpred [32] are also listed there. As we can see from Table 1, compared with its counterparts, although the Acc values obtained by the iCar-PseCp are within the ± 4%, its Sn and Sp values are more than 20% and 5–9% higher than those by PTMPred and CarSpred, indicating that the results predicted by the previous methods [33–34] contain much more false negative and positive events. Particularly, the MCC values achieved by iCar-PseCp are about 2 or 3 times higher than those of its counterparts, indicating that the new proposed predictor is significantly more stable.

Graphical approach is a useful vehicle for analyzing complicated biological systems as demonstrated by a series of previous studies (see, e.g., [42–51]. Here, to provide an intuitive comparison, the graph of Receiver Operating Characteristic (ROC) [52, 53] was utilized to show the advantage of iCar-PseCp over the PTMPred [33] and CarSpred [32]. In Figure 2 the red and green graphic lines are the ROC curves for the PTMPred and CarSpred, respectively; while the blue graphic line for the proposed predictor iCar-PseCp. The greater the area under the AUC is, the better the predictor will be [52–53]. As we can see from Figure 2, the area under the blue curve is remarkably greater than that under the red or green line, once again indicating that the proposed predictor is indeed much better than PTMPred and CarSpred predictors. Therefore, iCAR-PseCp will become a very useful bioinformatics tool for relevant basic research and drug development as well.

**Figure 2:**
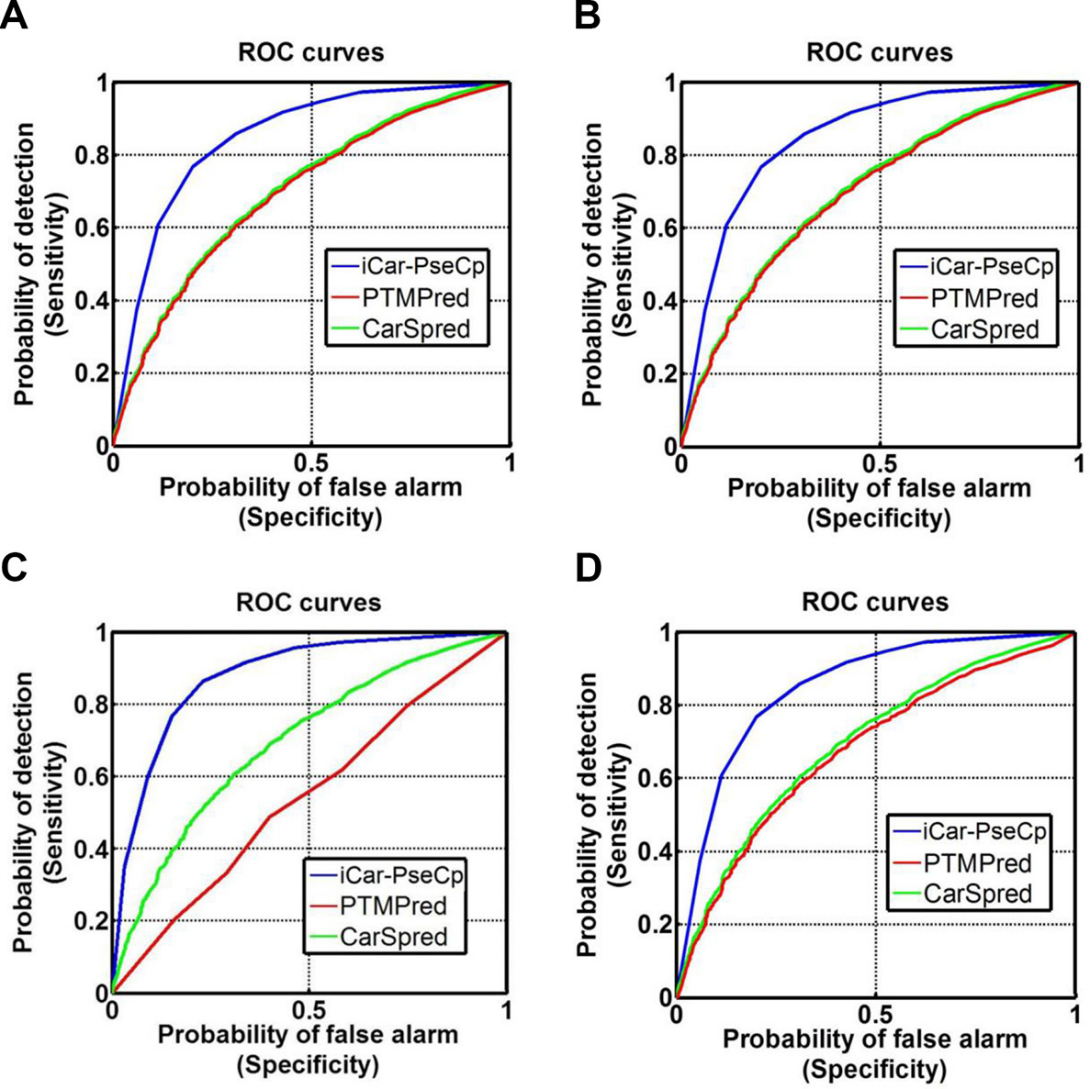
The intuitive graphs of ROC curves to show the performance of PTMPred, CarSpred, iCar-PseCp, respectively, for the case of the center residue is (**A**) K, (**B**) P, (**C**) R, and (**D**) T. See the main text for further explanation.

Why can the proposed method enhance the prediction quality so significantly? First, the coupling effects among the amino acids around the carbonylation sites are taken into account via the conditional probability approach, which has been proved to be indeed very useful in a series of previous studies [57–60]. Second, the predictor is trained by a balanced benchmark dataset via Monte Carlo sampling, and hence many false prediction events as occurring in the cases of PTMPred [33] and CarSpred [32] trained by very imbalanced and skewed datasets can be completely avoided.

## MATERIALS AND METHODS

### Benchmark dataset

The data used in the current study was derived from the 230 carbonylated protein sequences from human [26, 61–67] and 20 carbonylated protein sequences from Photobacterium and Escherichia coli [29, 63, 68, 69].

For facilitating description later, the Chou's peptide formulation was adopted. It was used for studying enzyme specificity [57], signal peptide cleavage sites [70], hydroxyproline and hydroxylysine sites [8], methylation sites [7], nitrotyrosine sites [9], protein-protein interaction [71], and protein-protein binding sites [72]. According to Chou's scheme, a potential carbonylation site-containing peptide sample can be generally expressed by

$$P_{\xi }(⊛)={\text{R}}_{-\xi }{\text{R}}_{-(\xi -1)}\cdots {\text{R}}_{-2}{\text{R}}_{-1}⊛{\text{R}}_{+1}{\text{R}}_{+2}\cdots {\text{R}}_{+(\xi -1)}{\text{R}}_{+\xi }$$ (1)

where the symbol ⊛ denotes the single amino acid code K, P, R, or T, the subscript ξ is an integer, R_−ξ_ represents the ξ-th upstream amino acid residue from the center, the R_+ξ_ the ξ-th downstream amino acid residue, and so forth. The (2ξ + 1) -tuple peptide sample P_ξ_(⊛) can be further classified into the following two categories:

$$P_{\xi }(⊛)\in \{\begin{array}{ll} P_{\xi }^{+}(⊛), & \text{if its center is a carbonylation site}\\ P_{\xi }^{-}(⊛), & \text{other wise} \end{array}$$ (2)

where $P_{\xi }^{+}(⊛)$ denotes a true carbonylation segment with K, P, R, or T at its center, $P_{\xi }^{-}(⊛)$ a false segment with K, P, R, or T at its center, and the symbol ∈ means “a member of” in the set theory.

In literature the benchmark dataset usually consists of a training dataset and a testing dataset: the former is used for training a model, while the latter for testing the model. But as pointed out in a comprehensive review [73], there is no need to artificially separate a benchmark dataset into the two parts if the prediction model is examined by the jackknife test or subsampling (K-fold) cross-validation since the outcome thus obtained is actually from a combination of many different independent dataset tests. Thus, the benchmark dataset $S_{\xi }(⊛)$ for the current study can be formulated as

$$\{\begin{array}{ll} S_{\xi }(\text{K})=S_{\xi }^{+}(\text{K})\cup S_{\text{ξ}}^{-}(\text{K}), & \text{when }⊛=\text{K}\\ S_{\text{ξ}}(\text{P})=S_{\text{ξ}}^{+}(\text{P})\cup S_{\text{ξ}}^{-}(\text{P}), & \text{when }⊛=\text{P}\\ S_{\text{ξ}}(\text{R})=S_{\text{ξ}}^{+}(\text{R})\cup S_{\text{ξ}}^{-}(\text{R}), & \text{when }⊛=\text{R}\\ S_{\text{ξ}}(\text{T})=S_{\text{ξ}}^{+}(\text{T})\cup S_{\text{ξ}}^{-}(\text{T}), & \text{when }⊛=\text{T} \end{array}$$ (3)

where the positive subset $S_{\xi }^{+}(⊛)$ only contains the samples of true carbonylation segments $P_{\xi }^{+}(⊛)$, and the negative subset $S_{\xi }^{-}(⊛)$ only contains the samples of false carbonylation segments $P_{\xi }^{-}(⊛)$ (see Eq.2); while ∪ represents the symbol for “union” in the set theory.

The detailed procedures to construct the benchmark dataset are as follows. (1) As done in [74], slide the (2ξ + 1) -tuple peptide window along each of the aforementioned 230 + 20 = 250 protein sequences used by [32], and collected were only those peptide segments that have K, P, R, and T at the center. (2) If the upstream or downstream in a protein sequence was less than ξ or greater than *L*−ξ where *L* is the length of the protein sequence concerned, the lacking amino acid was filled with a dummy residue X. (3) The peptide segment samples thus obtained were put into the positive subset $S_{\xi }^{+}(⊛)$ if their centers have been experimentally annotated as the carbonylation sites; otherwise, into the negative subset $S_{\xi }^{-}(⊛)$. (4) Using the CD-HIT software [75], the aforementioned samples were further subject to a screening procedure to winnow those that had ≥ 30% pairwise sequence identity to any other in a same subset.

Note that the length of peptide samples and their number thus generated would depend on the ξ value. But preliminary tests had indicated that it would be most promising when ξ = 7 or the sample's length was 2ξ + 1 = 15. Accordingly, hereafter we only consider the case of ξ = 7; i.e., the samples with 15 amino acid residues. Thus, the benchmark datasets thus obtained for $S_{\xi =7}(\text{K}),\;S_{\xi =7}(\text{P}),S_{\xi =7}(\text{R}),\text{ and }S_{\xi =7}(\text{S})$ are given in Supporting Information S1, S2, S3, and S4, respectively. Listed in Table 2 is a summary of their sizes.

**Table 2:** Summary of carbonylation site samples in the benchmark dataset^a^

Subset	Carbonylation type and number of samples			
	⊛ = K	⊛ = P	⊛ = R	⊛ = T
Positive	300	126	136	121
Negative	1,949	792	847	732

a See Eq.3 and the relevant text for further explanation.

### Incorporate sequence-coupled information into general pseudo amino acid composition

With the avalanche of biological sequence generated in the post-genomic age, one of the most important problems in computational biology is how to formulate a biological sequence with a discrete model or a vector, yet still considerably keep its sequence order information or essential feature. This is because all the existing machine-learning algorithms can only handle vector but not sequence samples, as elaborated in [15].

To address this problem, the pseudo amino acid composition [76, 77] or PseAAC was proposed. Ever since the concept of pseudo amino acid composition or Chou's PseAAC [78–80] was proposed, it has rapidly penetrated into many biomedicine and drug development areas [81–83] and nearly all the areas of computational proteomics (see, e.g., [84–91] as well as a long list of references cited in [92, 93]).

Because it has been widely and increasingly used, recently three powerful open access soft-wares, called ‘PseAAC-Builder’ [78], ‘propy’ [79], and ‘PseAAC-General’ [92], were established: the former two are for generating various modes of Chou's special PseAAC; while the 3rd one for those of Chou's general PseAAC [41], including not only all the special modes of feature vectors for proteins but also the higher level feature vectors such as “Functional Domain” mode (see Eqs.9–10 of [41]), “Gene Ontology” mode (see Eqs.11–12 of [41]), and “Sequential Evolution” or “PSSM” mode (see Eqs.13–14 of [41]). Inspired by the successes of using PseAAC to deal with protein/peptide sequences, three web-servers [94–96] were developed for generating various feature vectors for DNA/RNA sequences. Particularly, recently a powerful web-server called Pse-in-One [97] has been developed that can be used to generate any desired feature vectors for protein/peptide and DNA/RNA sequences according to the need of users' studies.

According to the general PseAAC [41], the peptide sequence of Eq.1 can be formulated as

$$P_{\xi =7}(⊛)=P_{\xi =7}^{+}(⊛)-P_{\xi =7}^{-}(⊛)$$ (4)

where

$$P_{\xi =7}^{+}(⊛)=\left[\begin{array}{c} p_{-7}^{+}({\text{R}}_{-7}|{\text{R}}_{-6})\\ p_{-6}^{+}({\text{R}}_{-6}|{\text{R}}_{-5})\\ \vdots \\ p_{-2}^{+}({\text{R}}_{-2}|{\text{R}}_{-1})\\ p_{-1}^{+}({\text{R}}_{-1})\\ p_{+1}^{+}({\text{R}}_{+1})\\ p_{+2}^{+}({\text{R}}_{+2}|{\text{R}}_{+1})\\ \vdots \\ p_{+6}^{+}({\text{R}}_{+6}|{\text{R}}_{+5})\\ p_{+7}^{+}({\text{R}}_{+7}|{\text{R}}_{+6}) \end{array}\right]$$ (5)

and

$$P_{\xi }^{-}(⊛)=\left[\begin{array}{c} p_{-7}^{-}({\text{R}}_{-7}|{\text{R}}_{-6})\\ p_{-6}^{-}({\text{R}}_{-6}|{\text{R}}_{-5})\\ \vdots \\ p_{-2}^{-}({\text{R}}_{-2}|{\text{R}}_{-1})\\ p_{-1}^{-}({\text{R}}_{-1})\\ p_{+1}^{-}({\text{R}}_{+1})\\ p_{+2}^{-}({\text{R}}_{+2}|{\text{R}}_{+1})\\ \vdots \\ p_{+6}^{-}({\text{R}}_{+6}|{\text{R}}_{+5})\\ p_{+7}^{-}({\text{R}}_{+7}|{\text{R}}_{+6}) \end{array}\right]$$ (6)

In Eq.5 $p_{-7}^{+}({\text{R}}_{-7}|{\text{R}}_{-6})$ is the conditional probability of amino acid R_−7_ occurring at the left 1st position (see Eq.1) given that its closest right neighbor is ${\text{R}}_{-6},p_{-6}^{+}({\text{R}}_{-6}|{\text{R}}_{-5})$ is the conditional probability of amino acid R_−6_ occurring at the left 2nd position given that its closest right neighbor is R_−5_, and so forth. Note that in Eq.5, only $p_{-1}^{+}({\text{R}}_{-1})$ and $p_{+1}^{+}({\text{R}}_{+1})$ are of non-conditional probability since the right neighbor of R_−1_ and the left neighbor of R_+1_ are always ⊛ (namely Lys, Pro, Arg, or Thr, respectively). All these probability values can be easily derived from the positive training subsets taken from Supporting Information S1, S2, S3, and S4, respectively as done in [98]. Likewise, the components in Eq.6 are the same as those in Eq.5 except for that they are derived from the corresponding negative training subsets therein.

### Expanding positive samples by Monte Carlo approach

As we can see from the Supporting Information S1, S2, S3, and S4, the negative subset $S_{\xi }^{-}(⊛)$ in each of them is much larger than its corresponding positive one $S_{\xi }^{+}(⊛)$ in number of samples. Although this might reflect the real world in which the non-carbonylation sites are always the majority compared with the carbonylation ones, a predictor trained by such a highly skewed benchmark dataset would inevitably have the bias consequence that many carbonylation sites might be mispredicted as non-carbonylation ones. Therefore, it is important to find an effective approach to minimize this kind of bias consequence. To realize this, we adopted the Monte Carlo simulation [99, 100] to expand the samples of positive subset. The concrete procedures are as follows.

Step 1. Suppose $P_{i}(A)$ (*i* = −7, −6, …, −1, +1, …, +6, +7; *i* ≠ 0) is the probability of the 20 native amino acids occurring at the *i*-th position of the carbonylation samples that can be derived from a training dataset in the positive subsets of Supporting Information S1, S2, S3, or S4, respectively.

Step 2. For simplicity, let us formulate the probability thus obtained according to the alphabetical order of the single-letter code of the 20 native amino acids (note that the dummy amino acid X introduced in the Benchmark Dataset section was treated as the 21st amino acid); i.e.,

$$P_{i}(A)=\{\begin{array}{cc} p_{i}^{1} & \text{if }A=\text{A}\\ p_{i}^{2} & \text{if }A=\text{C}\\ \vdots & \vdots \\ p_{i}^{19} & \text{if }A=\text{W}\\ p_{i}^{20} & \text{if }A=\text{Y}\\ p_{i}^{21} & \text{if }A=\text{X} \end{array}$$ (7)

Step 3. Generate a random number ℛ between 0 and 1; if

$$\sum_{j=0}^{k-1}p_{i}^{j}\leq ℛ\leq \sum_{j=0}^{k}p_{i}^{j}\ldots (1\leq k\leq 21;p_{i}^{0}=0)$$ (8)

then the *k*-th amino acid is drawn for an expanded positive sample at its *i*-th subsite. For example, if *k* = 2 and *i* = −7, then the amino acid thus drawn should be C for the left 1st sequence position (cf. Eq.1); if *k* = 19 and *i* = −6, then the amino acid drawn should be W for the left 2nd sequence position; if *k* = 20 and *i* = +7, then the amino acid drawn should be Y for the right last sequence position; and so forth.

Step 4. Repeat the above steps until the number of positive (the original plus the expanded) samples is the same as the negative samples.

At first glance, the rationale of the above Monte Carlo sampling procedure seems like a circular argument. But it is correct as elucidated in [54]. Particularly, these expanded positive samples were used only for training a model but not used for testing it, as well be further discussed later.

### Random forests algorithm

The random forests (RF) algorithm is a powerful algorithm and has been used in many areas of computational biology (see, e.g. [11, 12, 71, 72, 101−104]). The detailed procedures of RF and its formulation have been very clearly described in [105], and hence there is no need to repeat here.

For the current study, all the involved peptide samples were converted into a 14-D (dimensional) vector according to Eq.4, and then entered into the RF operation engine as the input. And the output would indicate whether the center residue ⊛ of the query peptide is a “carbonylation site” or “non- carbonylation site”. Note that, in using the current prediction method, one must observe the self-consistency principle: if the center residue of a query peptide is ⊛ = K then the corresponding training data must be taken from $S_{\xi =7}(\text{K})$ if the center residue of a query peptide is ⊛= P, then the training data must be taken from and $S_{\xi =7}(\text{P})$; and so forth (see Eq.3).

The predictor established via the above procedures is called “pCar-PseCp”, where “i” stands for identify”, “Car” for “carbonylation site”, “Pse” for “general PseAAC”, and “Cp” for “sequence coupled effect”.

As pointed out in the Introduction section, one of the keys in establishing a useful predictor is how to properly evaluate its anticipated success rates. To realize this, we need to consider the following two things: one is what metrics or scales should be adopted to quantitatively measure its prediction quality; the other is what validation method should be utilized to calculate or derive the metrics values. Below, we are to address the two problems.

### A set of four metrics

The following four metrics are usually used in literature to measure the quality of binary classification: (1) overall accuracy or Acc; (2) Mathew's correlation coefficient or MCC; (3) sensitivity or Sn; and (4) specificity or Sp (see, e.g., [106]). Unfortunately, the conventional formulations for the four are not intuitive and that most experimental scientists feel difficult to understand them, particularly for the one of MCC. Interestingly, by using the Chou's symbols and derivation in studying signal peptides [107], the aforementioned four metrics can be easily converted into a set of following equations [5, 35]:

$$\{\begin{array}{ll} \text{Sn}=1-\frac{N_{-}^{+}}{N^{+}} & 0\leq \text{Sn}\leq 1\\ \text{Sp}=1-\frac{N_{+}^{-}}{N^{-}} & 0\leq \text{Sp}\leq 1\\ \text{Acc}=\wedge =1-\frac{N_{-}^{+}+N_{+}^{-}}{N^{+}+N^{-}} & 0\leq \text{Acc}\leq 1\\ \text{MCC}=\frac{1-\left(\frac{N_{-}^{+}}{N^{+}}+\frac{N_{+}^{-}}{N^{-}}\right)}{\sqrt{\left(1+\frac{N_{+}^{-}-N_{-}^{+}}{N^{+}})\right)\left(1+\frac{N_{-}^{+}-N_{+}^{-}}{N^{-}})\right)}} & -1\leq \text{MCC}\leq 1 \end{array}$$ (9)

where *N*^+^ represents the total number of carbonylation sites investigated whereas $N_{-}^{+}$ the number of true carbonylation sites incorrectly predicted to be of non-carbonylation site; *N*^−^ the total number of the non-carbonylation sites investigated whereas $N_{+}^{-}$ the number of non-carbonylation sites incorrectly predicted to be of carbonylation site.

According to Eq.9, it is crystal clear to see the following. When $N_{-}^{+}=0$ meaning none of the true carbonylation sites are incorrectly predicted to be of non-carbonylation site, we have the sensitivity Sn = 1. When $N_{-}^{+}=N^{+}$ meaning that all the carbonylation sites are incorrectly predicted to be of non-carbonylation site, we have the sensitivity Sn = 0. Likewise, when $N_{+}^{-}=0$ meaning none of the non-carbonylation sites are incorrectly predicted to be of carbonylation site, we have the specificity Sp = 1; whereas $N_{+}^{-}=N^{-}$ meaning that all the non-carbonylation sites are incorrectly predicted to be of carbonylation sites, we have the specificity Sp = 0. When $N_{-}^{+}=N_{+}^{-}=0$ meaning that none of carbonylation sites in the positive dataset and none of the non-carbonylation sites in the negative dataset are incorrectly predicted, we have the overall accuracy Acc = 1 and MCC = 1; when $N_{-}^{+}=N^{+}$ and $N_{+}^{-}=N^{-}$ meaning that all the carbonylation sites in the positive dataset and all the non-carbonylation sites in the negative dataset are incorrectly predicted, we have the overall accuracy Acc = 0 and MCC = −1; whereas when $N_{-}^{+}=N^{+}/2$ and $N_{+}^{-}=N^{-}/2$ we have Acc = 0.5 and MCC = 0 meaning no better than random guess. Therefore, using Eq.9 has made the meanings of sensitivity, specificity, overall accuracy, and Mathew's correlation coefficient much more intuitive and easier-to-understand, particularly for the meaning of MCC, as concurred recently by many investigators (see, e.g., [14, 16, 38, 39, 71, 72, 108–113]).

Note that, however, the set of equations defined in Eq.9 is valid only for the single-label systems. For the multi-label systems whose emergence has become more frequent in system biology [114–116] and system medicine [117], a completely different set of metrics are needed as elaborated in [118].

### Target cross-validation

With a good set of metrics to measure the predictor's quality, the next thing to consider is what kind of validation method should be adopted to calculate the metrics values.

The following three cross-validation methods are often used in statistics to derive the metrics values for a predictor: independent dataset test, subsampling (or *K*-fold cross-validation) test, and jackknife test [119]. Among these three, however, the jackknife test is deemed the least arbitrary that can always yield a unique outcome for a given benchmark dataset as elucidated in [41] and demonstrated by Eqs.28–32 therein. Accordingly, the jackknife test has been widely recognized and increasingly used by investigators to examine the quality of various predictors (see, e.g., [84–87, 120–127]). However, to reduce the computational time, in this study we adopted the K-fold cross-validation, as done by most investigators with SVM and random forests algorithms as the prediction engine.

When conducting the K-fold cross-validation for the current predictor iCAR-PseCp, however, some special consideration is needed. This is because a dataset, after expanding by Monte Carlo sampling, may contain many hypothetical positive samples. It would be fine to use such an expanded dataset to train a prediction model, but certainly not for validation. This is because the validation should be made on a testing dataset that only contains experiment-confirmed samples without any added hypothetical samples [14, 104]. To ensure this, a special cross-validation, the so-called target cross-validation [113], has been introduced here. During the target cross-validation process, only the experiment-confirmed samples are picked out from the testing dataset for validating and scoring [11]. The detailed procedures of the target K-fold cross-validation (without losing the generality, let us consider K = 10) can be described as follows.

Step 1. Before expanding the positive samples, both the original positive and negative subsets were randomly divided into 10 parts with about the same size. For example, for $S_{\xi =7}(\text{K})$ in Supporting Information S1, after such evenly division we have

Sξ=7(K)=Sξ=7(1)(K)∪Sξ=7(2)(K)∪⋯∪Sξ=7(10)(K)=∪Si=110ξ=7(i)(K) (10)

and

$$S_{\xi =7}^{\left(1\right)}(\text{K})≜S_{\xi =7}^{\left(2\right)}(\text{K})≜\cdots ≜S_{\xi =7}^{\left(10\right)}(\text{K})$$ (11)

where the symbol ≜ means that the divided 10 datasets are about the same in size, and so are their subsets.

Step 2. One of the 10 sets, say $S_{\xi =7}^{\left(1\right)}(\text{K})$ was singled out as the testing dataset and the remaining nine sets as the training dataset.

Step 3. Based on the training dataset, use Eqs.4–6 to derive the sequence-coupled information. Also, based on the same training dataset, use Monte Carlo sampling to expand its positive subset making it have the same size as the negative subset.

Step 4. Use the sequence-coupled information and the expanded training dataset obtained in Step 3 to train the model and perform the prediction for each of the samples in the testing dataset.

Step 5. Repeat Steps 2–4 until all the 10 divided sets had been singled out one-by-one for testing validation.

Step 6. Substituting the average scores obtained from the above 10-round tests into Eq.9 to calculate Sn, Sp, Acc, and MCC.

It is crystal clear to see from the above steps that the validation was made only for experiment-confirmed samples, and that none of information from the testing datasets was ever used to train the predictor.

## CONCLUSIONS

The iCar-PseCp predictor is a new bioinformatics tool for identifying the carbonylation sites in proteins. Compared with the existing predictors in this area, its prediction quality is much better, with remarkably more stability and less false predictions. For the convenience of most experimental scientists, we have provided its web-server and a step-by-step guide, by which users can easily obtain their desired results without the need to go through the detailed mathematics. The reason of including them in this paper is for the integrity of the new prediction method, and that these techniques, such as sequence-coupled approach and Monte Carlo sampling, may be of use as well in developing other tools in computational biology.

We anticipate that iCar-PseCp will become a very useful high throughput tool, or at the very least, a complementary tool to the existing methods for predicting the protein carbonylation sites.

## SUPPLEMENTARY MATERIALS


