# Supplementary material for: iCar-PseCp: identify carbonylation sites in proteins by Monte Carlo sampling and incorporating sequence coupled effects into general PseAAC
Source: Oncotarget. 2016 May 3;7(23):34558–70. doi: 10.18632/oncotarget.9148 (PMC5085176; doi:10.18632/oncotarget.9148)
Supplement: Supplementary file 1 [file oncotarget-07-34558-s001.pdf]

iCar-PseCp: Identify carbonylation sites in proteins by Monto Carlo sampling and incorporating sequence coupled effects into general PseAAC

Supplementary Materials

Supporting Information S1: The benchmark dataset $\mathbb{S}_{\xi=7}(K)$ used to train and test the model for predicting the possibility of carbonylation at Lys site. It contains 300 positive samples and 1,949 negative samples. None of the sequences included has ≥ 30% pairwise sequence identity with any other in a same subset. See the main text for further explanation.

(1) List of the 300 peptide samples in the positive subset $\mathbb{S}^{+}_{\xi=7}(K)$

SAPAKGRKKSKMGFQ
ENSLAEDKDGRRKGK
VTTGIRDKKGVTVKT
QCRLKLLKLERIKDY
NIGYGSNKKTKHMLP
VTGGAASKLSKIRVV
QTTWRKYKLKTDLKR
RKLLMLKKEKLEKVQ
QKKWKIMKKAALLIQ
GIGRLILKEEMKARS
DKLTTADKLLGELQE
KLQVLPQKASERLQF
GITNLGYKVLIQPSN
LAEKVFAKVLREEDS
SERSGTPKKRKAPPP
AAKEMEEKISNLKEH
ADAGRDKKEKVFEKH
SSAINILKEKKKREK
GPRLGDAKLKEKFKD
VSGEHKQKGKVKRKL
QQEVVRMKLRLQHSI
DLQEQMNKNKMMPVL
GQQDTIKKALNYSTA
SKVTYLGKELLRYVS
DEKMKAMKRSRTWGQ
KRQLQTPKEKAQALE
KEPVGEGKGTKAFKQ
EADRTSSKKTKTQEI
QRVLDILKKSSHAVE
HKKPIDLKAIGKLPI
VKQTVQSKILTTGEN
KRLGRRFKKLDLDKS
IPVQLVFKNKIKLYW
SQEIKDTKECVQNKE
TRFLEEIKNQDKLNK

CNHIREVKSLLEELS
DCSQDLLKKREHHIE
KTLFSCWKSENEKLL
SPPAACEKGKEQHSQ
AKEHKEPKQKDGAKK
YRIELLRKVREQVLK
RKFVEHVKSQGHKDK
KTLNDSLKKVENKVS
RQICVTQKLLPAKRS
TEKEMIQKLDKLELQ
KQIEKELKQMELIKD
YDASVRNKQQELHLE
KHLKTLSKTVFSETW
RSIIGFVKLRSNKVK
NKVKRSQKLVYMGKL
YTKKDTKKNAKKSSD
CNKLLAAKNINLRLF
SGYEEALKIFQKIKD
PIKQVEEKPQRAQNI
EPTERPLKGIQSRLR
EPAPKKQKLSASVKK
DKLRISTKKLEEYET
LQIIPGAKHGNIQVG
RKLVERRKQVKQLMK
FKLGNKVKSEVNKLY
IEEKDLEKLDLVIKY
ECREEILKFLCIFLE
GLFAGPCKVINAKDV
MASLRRPKRIIIRGH
KVPADLLKRAFVRMS
GNGGVVFKVSHKPSG
IPEQILGKVSIAVIK
DSRSLAQKIVATYRL
NIPPLTMKRIRERFI
ISGLGGEKDRWTEAA
RNIYQGVKKKQYDIL
LKLESKLKADKLYLQ
EVQEVKDKAQKIVDE
KVLMPKLKAKPIRTA
LLNRLNRKMEIKPLQ
HFRSLRTKLLLMSRN
VIQEIVDKSGVVRVR
GRNQEKLKKFMIRIG
KKRIKKRKGEAMALN
TKEKAGPKGSKVSEE
ERIFTGNKFTKDTTK
KILKKINKAIVSKKN
VANAVFVKNASEIEV
CIVILTGKDPLGETF
TEKLAGVKQEVIKES
EREAELKKLQEARER
KKYLEDVKLQARGQL
AECVKQAKGVRQQAV
SISQKKVKIELDKSA
PLAGKKAKKPAKAAA
AAKARVTKPKTAKPK
FQQATKIKKLRAGKL
SVRGPRIKHVCRRAA
RFQLPLDKGNGKKHK

MASQRQAKIQRYKQK
VFDKVSEKLGDKRLF
QGKENLWKENLRKEE
SGKLYKTKSNKELHG
DRKTIPIKYPLKEIV
ASFQAERKFNAAAAR
GSLLGSMKLRKSESK
ESPHFYRKGTTPPRS
KKKYEKEKEKNKILR
IQYVVRLKKENVRLA
TANIQDLKEEYSRKK
ERLKRLQKSADLYKD
DILQALQKLSEDKNL
KHLQQKLKEKDRELE
GEDDERSKRIWLDYP
PDLRVVQKYPLLKEP
TNDFTKIKGWRGKFH
TLAPVVAKLGNSGAS
AKEADQLKQDLQEAR
AKKPAALKPKVPKKE
PGTTKTTKSSAVPPG
IDIYLLEKSRVIFQQ
INRALDAKLSRQFFI
NKLMTNLKSTAPHFV
HKLEGNLKLNRESME
LEVKSDSKEDENLVI
KLSLEESKKKGYDLR
KETYEKQKGHYLAGK
SQQMQSGKEYRKDYE
LLSVKQEKEIQMKMI
IISFAVQKETQFHTE
GEKYLQSKEDLRLML
LMLIELKKKQEAGFA
VTGREGAKDIDISSP
IPKMKMPKFSMPSLK
KLKGPKLKMPEMHFK
EMHFKTPKISMPDVN
NLKGPKMKGDVDVSL
EKAKSPVKEEVKSPE
APKKEAPKPKVEEKK
KARLERFKLRRMKAN
LQSQEETKERRHSHT
ADAKKKKKKSWVYEL
ISLKKAKKPPLPPSR
IEIKSKNKVRPGSLF
LAAMDATKLEYERAS
KEEKKKKKIKTIKSE
GLGVMPPKAGQTITV
GLEAAQIKELEELRQ
ELKAWQEKFFQKEQA
RKVEEVTKVCEGRRK
TLVPAGIKSLGLAVS
REVSQSRKPSASERL
RGGGSPEKPPSRRRP
KKFLPVLKEILDRDP
IHVIQQGKTGNTEKF
KIIKSSSKVHSFGKR
ERMKRHQKALVRERK
EMKKQTLKSLIIKLS
PPPIKSFKEMKFPAA

QKATTGLKPVDSGCV
IHKDLIKKPTISTAV
CRGIIGGKSQRVNGL
QLDRLLPKLARGSLS
SIVRIIGKMLPLEPC
KEKELENKLEELKKQ
KDLQEQNKKNEERMF
EGKDEDAKKSLRTME
EMSAKRAKKDVLHSS
LLLRMLEKRQMDRAE
LESMIPIKMVNFPQK
SIEKTSAKDLAPTSK
VLAKPTPKAETTTKG
MPRVRKPKTTPTPRK
KEERVSLKVLAKNFG
KNPKEEKKPKEKKKK
SRTVRLLKELRRALG
ARVTEVMKALKCNVR
LLLAPSSKKGKARLS
DMLRLANKDSIGFFI
GDRIQVRKVPPLKIP
TLADIKAKAQLVKAQ
KSIRAFAKGFLAEEK
QMYEEQIKNLEKENY
AVLLKTKKKGQKKSG
HGVCSVLKGSEFMFE
AMPGAEYKAKKAKGD
RLLDEATKRSNRDSL
TEHPYKSKKAVWHKL
LLSVRMGKEEEKLMI
MVTSLFCKLGVLVRH
HFLPLMEKLKKKAAT
EDGEKEEKAKEDKGK
VIPVLQTKTRTNVPT
PEPKKIKKAASPSPQ
TQNSYRAKEPILALR
DCENLLKKLLVLNPI
LLLAYKIKYPENFFL
ERKSPEDKKSMILSV
MYLMLDNKRKEVVHK
WSQRGKGKVFPKLRK
RGKAAILKAQVAARG
AYPRLVVKLMPNGLR
VPAAYVKKLDPAQSA
KKFDDFQKDLKANES
MAASRRAKLNESHRL
VEHWKELKQLAAARG
TELIRQEKLEQLAAR
LKQSGQKKSYTLRVR
KYDAIKFKINQLSEL
DELKSSVKTKDKKII
GRIRLPRKATKTKKN
RRKKRAPKADISKSL
ALMEVKMKEMKGSIR
SKKIPEEKVPVPVQK
EKPKAPPKGPEISEK
PIKGVPKKTPSPIEA
VVPRSEGKVHTLTLR
PKIKVDVKFKDTVIL
LKVTEITKDSVSITW

LDGGSKIKNYIVEKR
ESSVLRAKEVTWYKD
VQEEISQKALRSEEI
IGAARTTKKRIPNTK
GRNVKSRKLKEIFNK
PRPPSLKKKQTTKKP
GAVKGLCKLFCLTLH
LSSLGGFKLAHGLLE
HGLLEELKTVLSSHK
RLIGNESKGEHVPGF
KAADALGKLISIHIK
IGSINLKKNEPPLTC
FEALKYPKFSKAIVI
LTKQDKLKSPFKFSD
RKMEVGFKARGQPKS
FAKNIPVKSKNIRVV
SKKPDKEKPIKEKDK
EKLKEILKERELKIY
KKLALSAKKASTLWR
VNEQEPCKFLLDAVF
VFKTRILKIIDEGLK
LQLSKKLKTVLDQAR
KKKLTSKKPPGELLV
GLTVGFDKKDMMDIK
QQLNGVQKQIIICGR
DDVALVSKALQAEEM
TGDFESKKNELPDGL
AARRRFLKLQQDQQE
SKAKPSYKQKRQRNR
ETRESETKLKELPGV
ANRLFGDKSLTFNET
LKLEDTPKINSRFFG
FFGEGTKKMGLAFES
EKLIKDLKSKEVPEA
EKLTALTKKYRITEN
KFLDMLIKKLKSFDY
SYNETKIKFDKYKAE
IDLLKIKKITAIITQ
VSEHEATKCQSFRDH
YLAPNNLKPVVAEFY
YAVAVVKKDSGFQMN
MNQLRGKKSCHTGLG
LFSSPHGKDLLFKDS
LSHHERLKCDEWSVN
EGCAPGSKKDSSLCK
VPQNTGGKNPDPWAK
LCLDGTRKPVEEYAN
AVVTRKDKEACVHKI
YLGEEYVKAVGNLRK
TVKVPMMKRLGMFNI
RSVQLTEKRMDKVGK
SNTKVDKKVEPKSCD
LHTLFGDKLCTVATL
NECFLQHKDDNPNLP
LFFAKRYKAAFTECC
AEFAEVSKLVTDLTK
LLVRYTKKVPQVSTP
PVSDRVTKCCTESLV
CFAEEGKKLVAASQA
SEVAHRFKDLGEENF

KAACLLPKLDELRDE
SSAKQRLKCASLQKF
DSISSKLKECCEKPL
VLLLRLAKTYETTLE
CCKHPEAKRMPCAED
SEKERQIKKQTALVE
KHKPKATKEQLKAVM
DNEETFLKKYLYEIA
WLNGKTFKCKVNSGA
VATALAHKYHXXXXX
VHLTDAEKAAVNGLX
GMYELLLKVRPEQLV
EVERTRNKFLFLKAD
LTAFTNLKIKHPTYC
DICTLPDKEKQIKKQ
AADKTNIKNCWGKIG
KVADALAKAADHVED
KAGQYTDKGLRKCCE
MSAKEKGKFEDMAKA
DDSPDLPKLKPDPNT
KGACLLPKIETMREK
VGTRCCTKPESERMP
EKTPVSEKVTKCCTE
LVELLKHKPKATEEQ
XXXXXMVKVGVNGFG
TVDGPSGKLWRDGRG

(2) List of the 1,949 peptide samples in the negative subset $\mathbb{S}_{\xi}^{-}(\mathrm{K})$

SIAVQMMKRIHSLLE
SPHEQEIKFFAKILL
FGEQLIQKSEPLDAV
MASFSFLKDNSTDVC
HSRALLVKTTLNISF
IKVLSSSKVLSEEIS
ETWRLWQKFLDDYSR
RINEYVGKAATRLSI
EKQKSEAKDRKVLEI
EYRLDWAKWKAKIQS
PGEHVFVKGYEKGQP
GILDRSSKSQSSASL
ARLEADEKKQLCVLQ
QNLQDAAKDMKKFEA
FVILRDEKWGGNKTY
RQHPDTLKYSTLMDS
PTTLIPAKAPEIIDV
QIQALEDKKEKEMSA
THFVARLKSWRGNEP
WYNLLSYKYLKKQSR
AQPAAPAKVPSPGQQ
SPQPESFKTSRSSKQ
AARVTGMKKWPRTPK
ECLVSAQKVLEGSEL
VTSNEQVKGYGTHLM
NRGWFCEKVVILCPF
RILAAGGKSNHLHLW
NEMASTEKLTDVARY
GSKDREPKPKRKREP
QWQFLVQKSAEKSQK
HAPQFPEKEFNITVQ

KMALEVYKLSLEIEQ
RNTRILRKCIEKVAK
KAVMTSIKQLSSEEL
IRMSARQKQIMEIEE
HLSSPPTKFFVSTPS
LENSSGTKSAFVTVR
EELRDYVKARLKVFY
IRVCALNKVGLGEAT
VINYIVEKQDTRKDT
KCSKTSFKVENLTEG
FSQPIINKVKPQLLK
YRVEAAWKLSQWDLV
KARKLFYKAIVRGKE
NAEMHKNKLQYFMEQ
PRLPKLFKQEEQREL
ALEVDFEKMKKERDQ
YSAVNPEKDIHSGLI
SSVNSLRKAAHEALQ
RNTILWVKENKVPCL
HTLVESLKLSITDQQ
ALDEFATKLIQNNHY
EVLMVWYKQIEQVLI
NWCSQMDKGMLHFGS
EKAPATPKTEEKKDS
AIPLLAAKANTKNTS
ESIHNSPKSCPTPEV
KEVYQRNKSNCTIEP
KREPKEPKEPRKAKE
VSFIENSKFFEQYEV
ALLADSEKPSHKSFA
KLVVTGLKEGAFYKF
FESHQNYKDVQDPSV
PLQQFCSKKLFSIPE
REPAGDGKSIRTFKE
RYLYTLEKVCQPLYN
NDVLEHVKHFVINLI
QALEDIIKKFASVPE
KGPALSRKRKKEVDA
VFLTLYEKVFEDTYT
TKVPEVSKKIVPQKP
TEFQNHEKQESQDLR
QEWFLGAKAAAKESS
YGALAPFKPSEPGAN
TTILQHWKKCDSWWA
CRGLLLPKVVEDRGV
GDYVDRGKQSLETIC
NVLQYCRKSGLQTDY
PKPPSKVKSSSKESS
GRPLVASKKYRISFK
MQLPYRAKKFSLYCT
DLSGADIKAICTEAG
SKADAPVKWFKDGKE
DGFVDQKKKLXXXXX
DVQFYQLKLPMVTVA
LVNPAGEKAVFVNVR
KEGLLQMKLPEPVKL
RVNAWQAKANNNKQW
ITGQLGVKPQTGNSI
GCFWVIIKGCSPFLD
AISDVETKETTYSSG

SKPPGNLKECSPWMS
LRWTRVNKDYVVYDT
PVAAPEPKKETESEA
LGTNGTVKYSISAGD
EETARREKQQLLDVQ
HQSADRAKSEMATMK
EPPPSTVKTYHYLVD
ENYRPLAKTRQQNIS
LTMEINPKVPVNLLR
DGPLSPGKMEDISPV
EMDTDQLKLYEEPLS
LYRSVYEKNKMKIHI
TEFLALMKKVSKSPL
DNAPQFLKSKYFTPV
KPEELVSKELSTWKE
EKKNEVHKVEMFLGE
RGPQVDVKGPFVEAE
EMLTPEEKALLYEAI
ITLGPPSKPKGPIRF
IEKSKSLKQTDQPKA
RNYLPALKVEYNTSA
KRGAASVKTSGSPRS
LCNQCRGKKAKNPKE
EVCRAGSKHSRPIPL
QKELVLSKPCKFEEK
SRTRDVVKSALGFIK
STDWSGVKKPIYLSK
TGASGSFKLNKKASS
KKAKKPAKAAAASKK
GSELELAKMTMLLLY
YYSALSGKSISDGHS
KGSLEEEKRRAADAL
GSLEQIMKDRWMNVG
GETTHTDKVPGGEDK
PLLPSLLKCLKMDTD
SLESISTKVIVTQTT
TSKMDLEKPNYIVPD
QAHPVMRKCLQSLCD
FHLVRDVKQGNLPPG
IMEAPLLKHKDDIED
TGPPQVGKTGSYLQF
EEDWEINKDSAVEMA
RVLQALMKRFYLPGT
LSFINGYKNIYAEKV
PLQEAESKVSMALEE
VNRETDTKRPDARLA
EHSEIHTKLYFLQWL
ETADQFLKRSLEMRE
RGHEELRKLESTLDG
REHPFLVKGGEDLRQ
TLLENGNKQHQLGVW
ERSAEESKPRSLQEL
KYQALLSKMRAIDLQ
NFSPNQTKFTVQRTD
LVFCERGKLQPPRKA
AFSASARKVVNRDSC
EKPPPVNKQENAGTL
LLETGQEKMAGDQKI
SLLIGVFKHGYERYN
DITLTNDKPATAIGV

EVSEIHIKVKPTTKS
CFPEGLVKSCSETLL
TAFVQEPKVGETAPG
PFSSADVKPKVEDLD
DLLSLSGKTLCVTAG
SIEEPEGKLKGPKFK
INQVNTIKNEAEVIN
YQPLRRSKRRWVITT
TAAVQLLKPEGVLVY
NTSLLSQKKKLEADV
ESTPLANKPGKQSGH
SCQLSREKANVKWYR
DIAEQLLKANPPGKD
DLTTILTKLKAKTDN
RVMGFPEKSDIFDVD
EDTATSSKRRPRTRA
YSIQDWAKRMKALVE
NSFISIPKMEVKSYT
AGAQALEKLEAAESL
MTTQLICKLLRSREA
LRLLEPVKRAQEMDA
XXXMAKRKEENFSSP
IEIGLEGKGFEPTLE
RSVDSSAKREKPVVR
MESICIMKGMKPERK
EISESVGKNQFTSES
APVPRGRKGKKTKNQ
QSPAVLDKADGQKPV
KLNMMLSKGELLSTL
YTTQSTIKPXXXXXX
HTQAGWRKEGNLGTH
AGTERWMKVVTLKPT
KEAYEHTKAYGYTLG
WYRLRLLKPQPNIIP
EEFVTSYKALKSRIS
WNLRLDVKKNPVQDK
TSLAIAQKLMELKLG
KVEDLPLKLTIYSEA
VTACNPYKPPGPPST
RGQVDYSKIMNGNLS
GVIYTVAKEELKKFE
TWSKDGQKLPPGKDY
LANSAARKKKLLEAQ
HPVSGVRKEQGGGCH
IFTDSEIKDEAFLEY
GEVQLTAKDFKTHAN
SPTSPFYKACDTVFK
SHDNIRVKWFKNDQR
HMYKMPEKILPKILG
KEKQEANKQKVPNPS
ISFDDSSKTQSHSDA
GAGEEEAKGGKRPKE
GEEEVPAKKTKTIVS
VFLDSGRKTRSARRR
NMVLPDEKGAGALPF
SSEKLFVKYDLILTS
TNLTDDLKALYKVAG
FNEGPCSKILIQCKA
VVPGASIKYDAVKEE
MLQVTQAKKSQAIAS

KLLKELHKESKTRDD
IEAHEQGKDIDLNKV
EVREAAAKTFEQLHS
PIPPPDAKELELMFG
TVWRQADKHNIPRIC
PRIQQGAKTSLQEEM
QSLLAPGKFSPAGVE
IDFRLFAKLNAFCVI
TSTSDILKVPKPEPI
ELYSPVQKANPGTLA
GATCNNNKLSLSNAI
EELPEISKTKEAATT
QDPACSDKAPGMEGT
QTLAGTQKFSIRPSP
GKGSTFAKASFVASG
IIPHISTKTIDSWMS
ITSEERTKHDRQFDN
CIDDALRKNDFKPLK
NRFEDCQKEEETKQQ
HESTSVAKDKSSTAS
AAHTQSFKQPSCTHI
IQNVHASKRILFSIV
ETTTAAPKMTKETAT
QHTLNYWKEQSLNVS
YKADTVAKVQGVEFS
DFEEQFLKEKRFHDQ
IVALIAQKGNFSKTS
LKIEAENKYDAIKFK
QNEYNAVKEREFHNQ
IETLRLAKNYIWALS
GAWEQQIKQLEAELS
WAEMCSIKAVTAIEK
PVDNHRYKWNGRWWE
ILLNLLEKIPGKNAI
FAQVFLSKFTMVKNK
FLAGCQAKVEQAVET
RIEEALQKAAEKGTH
LRDGQTYKFRVLAVN
TLSWFPPKDDGGSKI
DATRLTDKLLELCNK
ENQTEINKPKAKRCL
NQTVTEFKQLISKAI
PEAKTPAKEEARSPA
SSSIFCLKMHKEMIF
YRCWRAHKEYLAILK
KKEEKEPKKEIKKLP
HTSVTLLKCTCTISM
QLWHHWCKKDKELYH
VSSPVIGKLNISETA
DYHKTKAKINIPADM
GHTFLLEKPPVPPKP
QVTMPGIKVGGSGVN
GVSVADQKGKSTVAS
FLAQADCKLLECRNV
ALHPNAFKRLGASLA
SSCIDMGKELLARSH
NASKEERKRWQATLD
DSGTYTCKVSNVAGG
TQILEELKKTKMDNL
TVEARLIKVEKPLYG

TSTTQHNKVSTSPKI
MSLDFYTKVLKSCRS
PSPVGALKGSDVILQ
DAMTHLIKISRIIRT
GENMTSGKLTVAGGA
ENRFGVSKPLESAPI
GTRPGDVKEEAPQEM
ARPAFNLKKPSKYCN
HIFFDAAKNLVWKER
VRYENNLKPVLKHVK
KYEGAKVKVLEERQR
FISDGVRKSLKVVPE
GIPPPTLKWEKDGQP
QELEQENKLFKDDME
QDNLGLQKQLDLQRE
SEAGVEVKKEVGVSI
AAAEENSKMEQSNLE
LAMAVIRKKHGMXXX
ELLETLQKFKAVDKE
WYQETHEKQQDLNIM
RSVLESLKRYRFGKD
LSSHHSSKRDWSKSD
VQVITHVKEKLHFMD
ECPDAKLKGPKFKMP
DVETQSGKTVIRLPS
IDYNPFAKGFRDDGL
LNELTQLKQLVDAQK
QLSDLNYKVEGEKLK
WQEKFFQKEQALSTL
VVSITCLKHKSGGHA
SASLAHMKAKFRETL
EEKQTSWKEIDNDFT
TDEDTDFKEFLKKLA
SCTLLSEKDSESSSP
EKASQLEKTGNDASK
FSFPASPKVFGGRLY
CTKPITCKDELAPPT
KVFERASKDTFQLEA
MSPDQADKLPQLQGE
EVLDGVEKMKSMSQL
LLEFHYYKCLVLGLV
DLSAAPAKQFNLEII
GFVLRGAKAETPIEE
ESFFSNCKSLPEAPL
QSLDRETKERFVLMI
LRYRQAAKPPDLNPE
HLNEVSLKLPLSDVA
LRIVVPLKDTRVKEQ
SAATQALKRNGAQIA
DSASIMVKAINIAGE
NTFSFAEKNFEVNYQ
ASLENCMKLSQMAVQ
IQLPVVSKQHCKIEI
RQSWSVCKQVFPGLN
MDWGAQQKKANRSSE
SGILDREKEERVSLK
LSLQSEHKILHDQHC
PYIAHSQKMQDLFSP
LTSEETAKMMVKIEA
RPEEEEPKVEPKKLE

DMMATLAKSQVTTVK
EGKEEWEKGKDKEVR
AAVSEDGKSDDELLS
QKELAEQKSVETAKR
AYEDLSQKYKAAQEK
NEEIDISKAAQTTIE
KGDKGRYKIVLQNKH
PFLVMIIKNLKKYFT
SHVILGDKVTKNSSG
TQEWDNSKSILGVQC
TPHLRILKVGKGDSG
HLLQYFLKFVPAGYE
TSNGLVTKALEHAFQ
RADQRKAKLGDSHDL
RGGSGFLKSGGEMLK
PDKEKPIKEKDKGQW
LLFGQRAKTIKNTVC
GEELVSLKEKSKSPK
EDDCLAFKVHQYFNV
FHLEFGEKSNAKIKD
YFTTQSWKTAQQHLR
EILQDLQKRLESSEA
AETEIVCKVVESSSI
LDLKINLKASYIIVP
VQAASSSKERGGVSL
RAEVHLRKSAFDMFN
FLCIFLEKMGQKIAP
PKKTRNLKKITREQR
IQIVYLHKFLMSLLN
GLDTPCFKTSVNDSQ
KREVELEKNTKEEED
SDVVWETKPKKKARW
DLSSQLLKVLGTRKL
GQSQDNEKELAALFQ
LILKEEMKARSSSYA
VANLEEGKSYFFRVF
VFFQSCAKAVMWQKM
LSSDDYTKKKKKGKK
TARFLYGKKVEGTAF
DGAKKARKPREASGT
FTVQLSNKLIMDIRR
EKEQLRSKLEEMYEE
QDLCSIGKEHVFSLE
VEEGLTYKFHAAWSS
LSSHDSIKMEIVDHA
GFIDDAVKKLNELSF
SSQDVESKRSDKTDF
QDLEDLIKAKEKEVE
DPMAGLLKFADDLGM
PPGQLQVKAQPQARM
EYDVTMPKVESEIKV
FTEPAIAKNPYDPPG
GHRKRASKSPRKTAS
ESTIGHLKDVVGRIA
QAEFADLKKLQDLTL
LPYQNMDKTKTDYTR
QKEIYQVKQQRLELA
KNDFKPLKTLLQIDI
EFDVNLSKANVDISA
PYMVKMLKXXXXXXX

RLYRKANKSSKLVSA
STGTLVSKREVELEK
TRVVHAAKAALAAFN
ADEMDRMKKEYTDCV
YYWGMESKIPVEDNK
DSFTSAPKQTPDSGK
FHKSVEIKPSDKHRL
ETQIIPGKGQEKPLQ
RENFDKEKGKTPKYN
TQQCVQCKESVGSGL
KVASLLGKKGASATK
IFENLANKADRDQYE
LQDQLLQKEQENAKL
CSALLSVKEPATITE
EGEEEGDKNGDGGCG
GRSEERDKEELEDLK
EEGLRSQKSDERQGV
VKKLEASKIVKAGDS
ERYLENYKCYRKMEE
EEKAKEDKGKQKLRQ
XXXMDDDKPFQPKNI
SLPGSITKAGDFLEA
ENLAGPGKPSKSTEP
SSHRSNFKDPLQVAN
HLCRIIEKKHVSLNK
GDRVNYIKRSLQSLD
DEVPPTIKLRLSVRG
AALDNLRKVVPCYSK
LSGVPLYKGDVPTQD
IIPTFQDKSLSFPQH
IGLHLVEKEIDIEKQ
GTDYQLSKEYTLDVY
KRRPQTPKEEAQALE
NDSQEIFKLAKDVLI
EEPWLEIKHLHESLL
VNTATCDKNPSASKN
TVVERLVKVTLYGSQ
TQDTTPFKITTLKTT
VVMAFSMKTMQNAKN
VTKNVRHKLTSRNER
AIITQGCKSLSSEMY
AFAICDQKSVHGTFS
VSTSQAGKLPTRITV
LKAMIMCKGCGAFCH
ILSLNQRKEDLLVDL
VVYVAPAKSLVGQVA
VSISSAVKSAPEENS
LPYFIRNKLCKVIVD
DELALFDKSINEFWN
YKDGKRLKNYNMPWG
LDDISQIKAQVKELS
IFDDWARKVQQRNLG
KAARGEEKDKETKNA
AESDTDVKLSIFCEQ
DRTRVPNKFRYPFYY
GKYTCQIKNDAGMQE
PVSASTPKIGAISSL
ERPDVDEKRSDLLKL
QIPKRNGKENGTENL
QKPVLDLKLSGVLTV

FVCSFLQKNRKHPSS
VKSMKEPKTEAXXXX
MELLEAEKERNNKLS
LEADIRGKPIPDVVW
YGEALGIKYPVQVPY
PTMSIDLKYLGVQLP
DFIQHIMKSSPLMFV
ARQSFFLKTYLDHEI
GPIIVEEKLEAPDID
IVTRTTIKDQEDLKW
THTELVIKEADRGDS
QAAFRGMKTRRHLKS
VTMQSVVKPFSIFGQ
SESSAPAKTEGRSLE
NSYAGQLKSARFEEA
PKFGFGAKSPKADIK
LDATPVTKGVASAVV
EDSDSPTKSSSTEPS
GYSISHVKRVLDAEP
RSPGGPTKLIEIISD
RQSNRATKEIQLHQM
IALLAARKNKILEMK
KSQEVEDKTKEYELL
SSNQEEPKAQGKVEE
YYRAYVSKKEFLSLK
WQAFLDLKKRIDDFS
ELWIRDTKYVQQPCV
QEMEAFGKKAANRSW
LVGIYGRKGLPPSHT
ETSQRISKLQEDTSA
LRKKMNLKPIMRMNG
KGPELDVKGPKLDAD
SKAMSVAKQVFNSLT
KKLINFMKMLSQKLK
KKLTRCLKPFENSRL
PDRDERLKKEKQERE
LTQLSLLKDTLSAYI
TETTEPVKASEAPSP
VFETLIPKPMMQHYI
SDEAEDMKEAMNRMI
ISNVSAEKATLTWTP
PGPLPPGKYMAGLNL
PYLEFRYKRRVYAQN
KLLESQIKQLEHGWE
LEKEKAAKLEILQQQ
EAQVRERKLQEEMAL
ISQTPLLKESGFRVD
ARPGLPEKEENEKSE
KKEAPPAKVPEVQKG
TRWVPVNKSAIPERR
EKYLTILKAVKVLQA
LNVIVLDKPGPPTGP
GLWLPLTKNVVGIDL
KEALKVQKNQKEILL
ESDSVVAKNAFVTPG
EASDVETKLREFEDL
LEVVDVTKSTVTLAW
STDIVPAKKWDNAAI
QKTTLVVKDCIRTDG
DASVPSMKVHAPGLN

KMVVAELKSELAQTK
LLRDLEEKVDKTELL
KVQINVVKLIAALKN
EDITTIFKTLHGNIW
VEANQAAKEDTVVLK
AELCGVLKAVLQRKT
QEQLNELKTEIEALK
ASDVSTAKSPFSAVG
GANTGIGKETAKELA
LESMMYGKKVLSQEP
HLEITIPKDLKLKEA
RVGAGPMKRENGLHL
VVFKVSHKPSGLVMA
IEDEQREKSVSHQTV
EEFCVFYKMMSLRRD
NILEGLEKLKHLDFL
YREDSNDKISEVSSD
LTNADEAKAQQELMK
PSEPTITKEDKTRAM
MSDQECVKRNREEEI
ELEELRDKNESLTMR
ELQLLNDKVETEQAE
HPGYALPKAQTTVTC
NLGSGTYKLTTMKKV
VKSPEAVKSPKRVKS
KLKMEIKKSRRHPLG
KAKLAVEKGRKARKL
LHQSRLTKLQSVQAL
FVVPDAPKAPEVTTV
SQDSEWSKREQIPLQ
SSSFPLPKQYLDVSS
CLLAILLKTTNRASD
LFKKLNFKRLGAGML
FGPAGTGKTESVKAL
ASTTLDIKDSISAVS
REAERAAKASSSAHE
VPLTQVAKEALREAA
LKKMDSIKGSFIDLE
ACPFLREKRGKNITT
LEASKVAKQGESIQL
WFLAAGRKWSCEAIE
GKAMHTPKPAVGEEK
QEMMSQIKYKKNALE
ELCRRLYKLHFQLLL
KASMASLKKTISQIK
AFDADMLKVFRNAEK
AVESIREKLKAKPLL
EIQQLLDKHKEYFQG
ADLTNPTKVTHLYNH
NQQQNHPKYSGQKNT
VHGSGPSKDSFGELS
MSVEEELKKDHAEMQ
CIPNEYRKGSTVKSS
LYGPIAFKDFILSKG
ECSLSLNKKSRSSTP
IEVVFVTKFLYSILA
FDAAMNEKMDLLNDA
YPNFEETKSLPWILL
LTAQASLKTKPIHTI
CVDCYRMKRKNCQQG

PIFVQWLKEKEEVIR
IITEAFPKPKDIPQA
IINITMTKKLDVEEP
KKFEALIKKDLQAKE
CSIQQQRKIAQYLKG
EWEESEVKLKKSKIK
WTLAAPFKEQHHHRG
AAASGGAKELKNIHV
RMASLCLKPPKTRRV
RCNKLLQKVQKNKEL
DKMSFVEKVNKMTAK
RYYHTLIKKDLNMVV
RPRSELLKAPSIVAD
ELVVSGNKRLKELSY
EQDSLNAKLEGVSDF
VTVENASKPDFTKNS
EEPGTEGKQLGQAVN
QTFYLLQKITKDNNA
IKDTEAQKVSTQKTS
DLLAYIHKQTEEGGN
VTRFEISKLTEHQEY
NGHVNFEKFLELAKQ
LTEKRMDKVGKYPKE
KTKNAVEKFTDAKRS
RPDLIDMKSVKHRSN
VRMLYYLKAEILGEA
VGTALPGKFKIVLDS
PAGPPPRKTSDAVVQ
EWSRCTEKMIKVRQY
DAEANQLKEELEKVT
EVYLVSGKNVGYAKY
LVSDVDYKNYLHQWT
KEKFEKTKGQPYAST
LSSTAQLKVLEADPY
KSQAIATKKIISDYH
TMEQALNKSNKLNMD
EIISDVLKIPVQLVF
NHLETCLKTQQIKSH
LIQELQHKLNQKKEE
QKNKPISKKEMIQRS
EPFSAMSKSTVSTSH
SDMMENSKEEGTSSS
VAHPLNEKMWSAVFG
NMVEELEKALGMKLP
LKQMELIKDQYQKKN
DSDSEESKKPRYRHR
YTCDCSQKEGVYRVA
PISVVWLKDRGEVIR
GLVYRGVKVMPFSTA
SSYFHREKSNIFSPQ
IEQFSTVKGVELEPT
QELIALAKNRFSLKD
ELDLWHSKLNELDSE
REQRRHMKRWEYFIA
DTRTHFLKTKAAVIS
PNSELCCKPLCLMLA
KPEKSLAKVEKELTP
PKIMSPEKSHRVISP
GKRHYEEKQKEHLDT
VFCAETEKELSKQWK

YELTEEEKFYRNALT
RRQLLLQKLLQRRRK
DLLLETRKPDQVFQS
ALHRLIAKSRELYEK
WRSSETEKHGQADSE
ELIAKMGKSXXXXXX
EKEVRAQKLADDLST
SPTEGKRKIIIXXXX
EYNTVVQKPRQILCQ
CQITGTPKIRVSWYL
MRYSAEWKSTVSRLG
PVAEVAPKAQEGEST
ATLMTQCKEGAQKEG
KKTNEILKGIKLLKL
FCDQQLRKEEENASV
AILTALLKGTNASAF
ARIHIPFKGRPTPEI
QTERGRNKDKAPEEL
VCNPHSGKEYFRVPD
EAMTITWKPPLYDGG
TSTGHKNKEFVARIK
HSEAPFAKSDNQPST
LNEDREVKNAEILRM
LHAVWEHKLGSQVSL
EVLHYSQKAQEKLLV
PLNSAAHKESPPTVD
LWLFEGEKIVLQVPA
PYHSHCMKVTAQNSD
VMDWINDKEAIVTSE
AFSTLIDKGTHVAFL
DSDINANKEELLRVL
HSLNLLLKSIGATLT
CDKRLWTKIDLSRCK
LLISGGRKGFTYVFD
QPLHRDGKLSLDQAL
DFDDLERKYWKNLTF
FGDTEEAKKQINDYV
TKEVEMMKAAYRKEL
VSSSMEFKYDFNSSM
CPPSIDLKEFMEVEE
NFSSVLYKEDVSPGT
APTIEWYKDDKELQT
RNLPSDYKYAQDRAS
PGQDNLVKQLRDAFD
ELEGQIAKLEEQWLS
TPDLLRAKRGQKLQS
ITNYIVQKRDTTTTV
STSDRAEKFCGQKKR
NALDFVTKSYNETKI
EHLEHEDKQNRLRAL
QASLHVWKIVVSNTP
QGEQLIEKSEPLDAA
FVQAFVNKDPLASTK
RSEVSQVKREKENIQ
KPLAASPKPAGLKES
RQRNRKNKMSLALKN
STVEVERKQVHILQQ
AVDPTLLKTFEALKF
MEKEHLNKVQTANEV
QHAELRDKTTMLGKA

KGEVETLKAKIEGMT
PASGADSKDWLAGPH
DYIPYVFKLLKENLC
ARQLLREKHKASIVI
QALELPQKAEALKLF
VLTEDALKSLENQTY
HTILPGSKTREPSNA
ESPSPAPKPRKVELS
PDSTVEFKAILKGTP
ENHTPEMKHFVGLEK
NYFAIRNKKGTELLL
SAVGEHEKVINNQKE
EEEYEPEKMEAEDYV
LGREVGAKFWELLLV
YKRAMEIKEAETSLL
QPGPDYVKQRFQEGV
EGLQPAAKAGTEDEL
SLSQGRIKTFSQSEK
VPEPILRKIETFLNH
YSDDEYIKKIQKRLE
RETQGIEKLVLINKS
RPEGLREKNSVIRSL
LFLPFSYKNKYGMVA
ETTSASLKKRHADSL
ELQQLDSKFLEQVHQ
QVPTPVVKQTKLKQR
CFESSETKKSVEQKL
QLQLRHLKAIGKYEN
FADIPTGKTSPYILR
SRSFREEKDRSNKAE
LQINELQKEIEILRQ
YSPSPPPKRRTASPP
KVVSVTRKPEEDGAR
CLSAFLDKYELQIPS
LLSIKWNKLEDVAQL
VTDVIEMKDRLVSPD
VQSLEGEKLSPKSDI
DCIEAEEKVHVIEKK
CVLRLGNKEKTSTAK
LLSLLQAKVASLTLH
KAALKTQKTSERKYE
VREEILQKEITKLLE
RLFIISTKAGSLGIN
ESGPEQSKQKKVAPR
EGFFRSNKVFVESSE
LCLAVPDKTVRWCAV
VSELATVKRPQERSY
GVKQEVIKESKVEEP
NPNSSGRKKLTDSPG
TPQILHAKKSYDLQS
NVTFSFVKYRDTDTN
GFVYIAGKCGLVPVL
RASLKRLKPHVPLGR
EKKLLMFKRVQEAED
DSCVVAWKPPASDGG
FCIQEIEKSGGSKEA
FTSLEVRKVAETKEK
FTGDEDIKIPENPLE
EELAQFLKVEQKMNN
KKEAGNKKSTPVSIL

ITCPADPKKTLGIKL
DVDDLIFKLAYYEIR
ATYLLDGKVVNVAGG
IDHPVHLKFNVSLGK
GSMNVSVKLKTCTLD
KKLTKMDKVLGSSIH
SWMPTLEKICEEVIV
PEHLRTTKVNKQISD
ENAAGVGKPSHPSEP
MLEQTLQKVTELENR
SLEMPLRKQEELEST
KAEHVKQKGHYVGVP
XXXXXMPKSGFTKPI
KLEYSSFKTNQEKEF
IELFSTEKKDKNDSE
KMPQDARKILSNQEM
LLDIILAKNPSGLTQ
ERQVSHAKEKLTALT
RWRKLVSKTQLEMNL
NYIDEREKSISELSD
PIEECRYKLYQEMFA
QDRIPSGKQHQETSA
ASDRLTMKNDHISAH
LQMISACKGETGAMV
VPSDEKFKCLQDQNT
EDDDGSLKNVHMAVI
KELNLLQKLYGLYDT
TVSIMLTKYAAYNTF
SKAGVPSKSSGSAAL
STFDYSLKDRAPFTV
LFQQQGSKQKLHDCL
DEEDEEAKAWLQAHP
EVINRIQKLRKKCNL
IKVDVKFKDTVILKA
LPLMESGKILPGVRI
IRTALFEKVLDKIVH
AGDGKSIKAFKESAK
VKPQAKKKAGDQEEE
IMIDRLEKASSNQLV
ANDLAAQKTKLWSES
KAITCGEKEKQDLIK
PISITWLKDDQILDE
ATTTEEAKGRNPRAG
QIIQIEDKTTIINNT
ASQELDVKRREAIYN
AVDELNQKKEGFRST
EYEEDRGKGFFPQTI
SRSPRRRKSRSPSPR
RLEERARKLQETNLR
VMYNAESKGTSAQTW
IFNQLEVKNPDSKMM
ELLSLVYKGIAPGDE
WDSPMRVKLSIWKPY
GISDLLGKSGITEDA
SEEADSLKHQLDVVI
NDEFEIEKPKAYIPD
AGPTQGVKAYPDATM
RCFVLQPKEKIVISV
RISSSLGKKPSLTSE
VLQDLEDKIRALKEN

CCPDMITKFLGVIVK
GVAQANIKNGKHLLM
DGTSANSKMEGLESK
EASEGSIKLPKMKLP
QYIFEFDKNDRLSSV
EQSFYQLKVVASGGT
QENTLTVKNKEGEIH
VLSQVYSKVHNGSEI
RHSDGTEKNKLPSQP
IKEVEQSKATSQELS
LQTLLDTKFVHFIDA
PIGAAASKEYSLEKN
FKSVLLNKTIEESLI
RPGQQDGKEGSQPPP
NPVEARSKYDVPGPP
AYLGPTVKPDDKAKT
GTSQISQKIIPLLEL
VSHSPPPKQRSSPVT
FGGRNIKKQCCKMRK
TVTIVDSKRSDTGTY
EIIADGLKYRIQEFK
QTNYRAVKEQLKQWE
VSGPADQKTGTATVL
DDEPEEGKKRTGKQN
DVIEVTGKXXXXXXX
RGWTFHAKGLWLYLA
LILMIFSKLLKHSVI
DTGERPLKKSDKSNK
XMDVKERKPYRSLTR
GEVTEAGKAYVPPRV
QNIAAFLKENHDIDD
FANFSGGKITKDVGY
LFNTRYAKAIGISEP
KDTTFRDKKSSLMEE
EPGVPTTKTPAATKP
EFGRLHCKFVVQTSP
YSFVIDGKKTTCKLK
IISQFTEKIEKMQEL
VTGYPQPKVNWYLNG
ANVSEGEKKTEESSQ
AKRAELEKLKSQVTS
HARDVVAKLSEDRVS
CCELAFCKIINSYCV
DGEGRPAKSQEEGKR
GYEKKRSKLIGAYLP
SAVAPQRKMPLDDTK
YRQNQELKVRVELLH
KSPKADIKSPSLDVT
IDSVECNKLLAAKNI
RIKYIEFKKSTVILQ
KRLQILLKGYGEYPT
DAIVTWYKGPTELTE
LPYMIRSKLKLLLQG
XXXXXMKKEGSSGSF
YSYETIGKTTKTPED
PRTEISDKITSELVS
KKDKKDSKTDNKKSV
NDVQSELKYKAEHVK
VTHEQIRKETEKTFV
QGLNFTSKTAEGHPK

RHHHLVRKTDAVYDP
XXMRLLAKIICLMLW
SLLVAGLKKEVQALI
LKVLGTRKLMHVAYE
HDPIMGNKYAANRKS
VAGSDTTKSKVTIKD
RELRERMKPGFEVKP
LTDLQVIKNETDARW
IACNFESKYQCISKS
SELSDQYKQEKLILL
ERREVTGKWVRVNKT
KTSEDIIKSKNLRSI
TENAIMLKRFNRYPL
PIRFDEIKADSVILS
QENEENSKNISTMES
LPSEDLLKELQKQLE
TILDDPGKKVKKPAP
GESTTDWKQIRSIIM
TTVGLLSKDSGKKLG
QLQTTFTKALQDSNR
DDTYLQLKKDLEYLD
QEKPSRLKQRFLLRR
NYFHEILKTIEEEVK
PTDFEWLKQSRFYFK
VTDYRDMKSDSVAKL
SEFVRFSKTENKITL
SYRTYSEKEGPEKKK
VLYDYVNKYHWEHTG
VIRTTHFKVTTISAG
SLSPQRSKLRIMSPM
PHDIPESKDCVLTIS
KLAAQMQKLNQKRAE
VNCKVLDKPGPPAGP
EESLIYRKKYILSKD
KAHVTDPKADPRALL
VAHIIDPKAISKDHL
KHLASHIKYREEYEK
EFQREELKAKQVSLT
FPNGVERKDAGFYVV
QQISFTEKGSDEKKP
RVAAVNVKGMSEYSE
EKQAELDKVQAKFDA
KEYTIVVKVLDTPGP
RKLALTVKGKELSEE
VTEVTSEKCVLSWFP
PDLDLNLKGPSLKGD
DLGPERRKSTTELTI
GVEIISIKPSAGHLS
PTMRDDPKLVWFEHA
PYWLDEPKNLILAPG
LENAASLKLFAGSLA
ISWLREPKSKPQLID
REQQLVEKSGELLAL
DPVQEPNKSMFEKTK
SVSKSGRKIKGRGTI
SEHMDDSKQESTTSK
FEGNTNTKGHVKNFF
QGVLDTGKKLSDDNT
LFSKPASKEDLKQTS
WFPASHVKLLGPSSE

MHISSQIKKTTDLTT
HIMDQHWKFCVECEE
EEVPPPPKVPALPKK
RQLENSEKYEITYED
HEENISSKMKGLNGK
EEVQLTSKQKEMLQA
GKVLVREKRVDLIQD
SPGRCTPKSSEPAPA
FEEPPFEKEANIIVD
KEVWNREKLTLQKSL
GLALIQNKKEDVSSI
GILEHQYKESTTYIS
AQSQQLQKHFSSLEA
LQVANFLKDSYTALT
SLPPLPFKRGGGKDG
KAENDRLKVAPGPSS
SRATPPTKAVDPIDA
HDAHLNGKVIGTLKN
DKTSDGPKLFLTEED
RGTSLVVKGLKENVE
VNWYFENKLVPSDEK
EGELESLKENLPFTM
ETISTANKANAERLK
ISLPNIRKPDGYDYY
DSAHGFLKVPPRMDA
QAWSSLGKRADQRKA
NPDDGARKPIPRDKV
LEELSGAKISDLHRE
EEHMEQQKENMEHLK
SLLCNFTKSMEEDPQ
LTENQFLKQLQLRHL
IDYYIVEKRETSHLA
HKRRPKVKRSKKRRK
LSPAVRQKETVKNDL
GHQNLHQKIQYVVRL
SAVSQERKLQNSMLK
KRLEQQRKDYAAPIP
NDWFSNIKVNLKECF
TAFYDSLKLVRAEQI
RQPPDRFKFTSVTDS
KMRVLQEKKQKMDKL
LEAIGAPKPAYPYLE
QENLNSMKKRNVNFS
HPVEGHQKLDSSPDP
IENQLAIKSKALDEL
HYLQENLKSMEEIQG
RAPEHSNKSKDRDNE
VVLRQMSKHKTELKN
MDAQTIAKLEKAFSE
YSHGENHKLVSEHVQ
EDDVTDPKRPLSFGV
SNMPQSLKVGSFIIE
TKGGWQQKSKGPKKT
YTITLENKLGSATAS
VKKENRGKGGAVSKL
YRTYRAAKLQRENYI
YDYESYEKTTRTSDV
NLRKQYEKSSRFMKV
KPGEMEKKMKILRES
GPFTVVVKESCDGMG

FEELVEMKSNYYSFI
VATSRSEKEQLQALN
LSFGVPRKAILYPHE
EWLRNIEKVEVEAVN
AIAAQVWKLSTCDLC
DIHGPDAKLKGPKLK
ELHRTLSKVNAEMVQ
KGDSPELKPRAVHKQ
NFSAAETKSDKKDCA
VPMTEETKSITLFPD
NTARLEIKSTDFATS
DFQAANVKSSAHLRV
YDEITDLKQLTVVME
LNTLSEAKVETICHR
FRTWQTRKQFLLYRK
NRLAALPKGRPDRPR
EIVATASKVDIAPHV
ALTILRRKDESETEW
LPLHDSLKLLQEQLT
TNRGHTVKAVCESFH
VVLPPIMKQPPMFSP
CGAQAGIKISSVHKR
DPPPFMFKPVKEEDD
IIHDTQFKAQNLEEG
PTLLSLFKHECSRVI
LKALRVRKKTGGEKM
QQKELLNKRNMEVAM
IFPSNYVKPKDQESF
EASKEIAKLTCVVES
PTVTWLRKGQVLSTS
RVAELQRKPTRESEY
CTAQLNIKERLIPPS
DSSLLKTKRKHKKKH
VSRAPITKVGLKGVE
LTRSPRVKIDTRADS
PFEVTWLKNNREIRS
XXXXXMSKLSFRARA
LAMETIDKAKEVGAT
LSMSTHFKMLKKLVE
EIYRWPIKESLKAMI
ECSSSQLKASVCKPF
EGILMHIKPENHCSE
HPDNQQLKNEQEESI
LPARQLWKWFGKPTQ
INFLENLKILEISED
TVLLSMSKGGKRKDS
PPATPPPKTRHSPTP
QMYEFRVKAVNEIGE
LAIESCLKGLLQAYC
QPEDDDVKGQWDRLV
QYRLLSLKYHPDKGG
PVRPSVWKKHGVVNT
TPTYITAKESQQMQS
QGLASLRKSANKLKP
WAGYLITKVRHDLAS
KRMAQDLKDIIEHLN
SLSLSNVKGHGRADE
PTPGITRKEEAPENV
KSGRSKEKRKSKHKL
KAREFGKKVQEEXXX

VIQCLRSKEVFGFYN
AKSPAEAKSPEKEEA
EVTIMEEKERAYTLE
NAKGMVEKWLQQVEQ
LQEQIVQKNQEIKNM
KKEEVEQKKNEYNFK
KVKSEVNKLYKLLEI
KLQRKKKKRKGSDEE
SEEDLSGKHDTVTVS
DITMWLSKRLPQFLQ
RKSKHKLKKRRGHLS
DHLRNGSKPKRKNYA
QCDCWLSKSEGDGQT
ETYPRPTKEDVYVVG
SIRENGAKSQPDPFC
QVQITQEKMRKEAEK
IGVLAQSKFQAVRKK
ELHTEPMKLALSIEA
RRAPSPVKPASLERV
QVIDMSVKSLTLKVS
PDRTVAEKRGRKITL
KKEPDDAKAKEPSKP
KASLFINKEDVYEYY
IDIHYGVKSNSLAFI
MAGLGVEKLEKLMPE
LSCSQYHKMYRTVKA
PGFQGRLKYNAFCEL
LEVLSGQKLPCEQGR
NWLSNAIKEFGFSGL
SAMQSLIKYSGSFAR
QMFLNDYKEVPFDAL
RKESITEKKPKKGLV
EKQAQLEKELSAALQ
HALAIAEKSQEFLEA
SNDSTHFKALHSNQA
LFEVALPKTAHHFVI
SPREESNKRQRTYXX
LLPTPEEKKPPPKRI
KLKEQLTKVKECYSP
DMNIRGPKVDVNAPD
HDKTAVEKDEITLKC
KGYVVEVKEAAADEW
EEEPAQGKGDSIEEI
SLHGTVMKINQERAE
EPWTTDAKIQNKNCP
PGIDATTKLNVGAPD
RARGHAVKNSSHVDR
QVFFNMVKETTSNCF
SEECTSRKPGQTVNN
MFCLEQEKRLPFSKR
GSEQDGWKAEPGEGT
RCICGLYKDEGLMIQ
SLQFEEDKENSSKEI
QSILEQDKLTPESTR
VFVGRAQKNQGDRSN
LTNVSRAKAEDALSE
FRVRAVNKAGESEPS
PPVTSPLKPNTPPSA
KELDLCEKVSLQYTG
VTPPAGLKATSYTIT

TETQNALKEEEERRK
SLEEIMDKAYDKFDV
LYCSMNSKHLNGTIT
CNNVSSDKIGDKGLS
PKTAKPKKAAPKKKX
NIAIEFNKPQYFSIM
LINGAEEKILEDFRK
ELEALNGKHQQISGR
SASPAVTKDQYSATS
STETIICKDEYEAPT
PDVNVNLKGPKIKGD
MHIDTVLKEIAKTVL
SFQAANAKSAANLKV
DLASLPEKAAANEEG
FTLPLYNKTAYVGEN
AHREIEDKPVATSNI
AYFVPMSKIADFLKA
SQYTYALKEKAKYQC
ARLDQMRKFRRQHEQ
DLEYLDLKMTGRDLL
HMVALLDKDQAVAVQ
KEIDMLLKEYLLSGD
QAMGHRHKAGGAGRS
SMLEERIKRSAKRPS
KKSLLEEKERFQREV
ASSMTEMKFASMSAQ
ETSKVTLKEQQHQLE
DRLITLSKKKAGHDQ
CYYHVISKVPRIRTP
PQKTAFLKDRLNAIQ
KPSVGEEKDIIIFVG
ESLMQSLKMNIFRLQ
STAELLVKAETAPPN
EKLLRESKELADLAR
DTVVIASKPYAFDRV
LPTKASSKAELEIAN
LYICDYHKNLIQSVR
VEHPQRSKKAVWHKL
EACIELEKIVGELKK
TRHLDEMKSAMRKGH
FLLNEIEKEEKEKLW
CEVTGTIKAVDRLTA
STQTESNKKMEENEK
LPESGATKVEDMLTL
ALCSWTAKKDNHLNF
SGLDRMAKHLSQRKT
RLQICDIKPRDQGEY
NNCDYEQKERFLLFC
KQEIMTLKEEQNKMQ
KIQFLKMKIAEKQRQ
TVAQYPFKVPGPPGT
ERSDTNEKRSVDSSA
KNLWVSQKRQQASFP
NRDSQSEKDDGSDRD
SSRESVSKAGMPVSA
EDLSEELKKENPFLR
LAVEIRQKSFGPKHP
RQENMVLKLANQKNK
KSEAGGLKQEIMTLK
EATENTLKEASVESE

EGLKDDEKLEVSELA
THRDSGFKNVGDITN
NCERELHKLEGNLKL
LSNLEEAKKKKEDAL
NAKCIHTKFQVFKHQ
KEEAQLQKCLGQVEH
EITVSWYKGDTKLRP
TPEIVLAKNNALTMS
ANVEGDLKGPALDIK
LLLPVLIKEQLEPPE
NSAGLLIKAYNRAGS
DLVVDVGKPLTMVVP
LIEPSVFKEAATPEE
GNPFQANKHGERPVD
AELRIELKKANNKVC
EIIHDRIKREYGLET
HVYYSRSKTTHIIAT
DSEVDNQKRGKRHYE
DSGNYILKAKNVAGE
GTRNYYRKIGYRLQG
SLLESVQKLDLEATI
NENSNRQKLEHVKVN
VGASQECKKEADEQL
LQEERTCKVCMDKEV
PSLDLSCKQLASGLL
MEVHEGDKYRMHSDR
RQYCFYQKLQWATKI
YVEPHELKEVSQTGD
IQNERLYKEDYHKTK
TVHDFPGKKLVLSSE
GPPEGPLKVTGVTAE
AELQEMWKRLGHELE
YFHISPIKLHLSVSL
DAELRNTKWEMAAQL
PQVSGELKGPGVDVN
RRTTQSTKHGEVDAW
SDNSEYSKSEKGKNK
PKVEGEMKVPDVEIK
ENGVGEPKSSTVSVK
VRQDSPTKRPALCIQ
STTKRSPKPPNKKKT
DFLAEIFKKIGSKEN
SVAWFHEKTKIVSGA
LDYELCQKHEMTISA
TPHSHREKPGIFYQQ
ENLVLQAKIHMPEKT
VVERRDIKRKTWVLA
TTASAHLKAGCPMLA
NEARINAKAKRRLRK
CCLTFLVKVKSKALL
LRQCMLPKSAALSEK
AYEEQKKKVADHPNR
MLRDLQEKINQQENS
QVFNQIVKSRPHDNI
PQCVAIDKLKKCKSS
SDDYDRIKCHVDEQS
CVGSIALKAPPRFVK
QFMGQAFKSIHEKVS
LPTRALQKIQELEGQ
GHLNPSEKLQKLNEV

LEQARQEKDKLPLEK
PAYDGGSKVVGYIIE
ARHSGHHKKRRKEKK
FDTDTGLKQALETVN
IPQIILAKANAINMS
FEDSGNPKSPDKAQA
SNINVEAKFNTVVSQ
QQQIRPIKSVIPSPT
QLKASLGKVGVKEEL
AVYAVLDKVKTKKST
IQVASNAKVAASRKP
SIEHQEIKHTEFLKE
LYCLSDEKLIQKISX
IPATIQDKLIIRFTV
PQQGTQMKTKEFTSP
LKEEYSRKKETISTA
LSVSESQKGEERDRK
SVLRECDKLRHDGYR
WEESQTQKIQNLEKL
KSTRSSVKLQGTSKI
GTDCLEAKEKVHVIG
VFQREQTKQVEELSK
SSEPWEYKSSGNGRA
PCGRWLAKNEDDGSI
AGAKSRPKKREGVHL
SEPSEFYKAADPIDP
GNQTTALKHAKDVKD
RGPGRPRKRTHAPSA
VTDEPLLKMLLTKAD
GMDNQGHKNLKTSQE
LPSPCKSKSLGDLTS
KPLDLRPKKTRAMRR
STFVDQCKTVLKLAS
FRICAINKAGVGEHA
NEIHTNHKTLYDLKT
NLQDDLEKQERSLQK
KEMTNHQKKRAAEMM
IRTEQESKGPMTRRL
KVEAPPAKVSKKIPE
EKARDREKAQELLKF
SGGLIPGKSLVFATM
MVVLVQEKENDYYCS
LRSAPAYKMQFKNNV
KKRLERIKSSRKXXX
IVCPFFIKTGMFEGC
SNCNGEEKKLDDSCS
WLQETDHKSIEDQLQ
CLHVSAQKMLDGASF
RKYDKRRKKRAPKAD
EIATSQLKSDLTSRD
LVQWVVQKVAWFLQI
TFAFNQMKPKSIPYS
AFQDHPEKSEQFEEL
CASSLPIKKIAGSNY
ETRRYALKNVTILVT
LDENIFPKQISLRQA
RKVEEIVKDHLMYLD
LGPEEVRKSALTLVM
IISEMESKLVEQQLA
PPLVPAIKEDAGGMI

DGYEFFAKRQLVTLF
PALQMAFKLGLSYTP
MHSNSQDKEVTILAE
IDGLSGEKIRWTQQS
GIIVRWCKVDDDFTA
ENLEFHIKVSYFEIY
EKQIEQQKKIQMSNL
KRRGPKKKMTKARL
MYLNILRKKITVNSS
SDTVHQMKPYLKKSV
QKPTKAPKKPTSTKK
KDKPLIYKGEGKWLR
QDLRATAKVPSPPDE
FDSRGDVKVIMATNR
LLGQAEDKAEVDMDT
PELVQAVKNTAMFSK
TSTLRHCKYSVTKLI
LDERAFEKTLTPIIQ
DFLDFMTKINGLEVQ
LRWVRAIKTPVSDLR
PEMTTTAKDKTTERD
AASPLEPKELEELRD
HTISVRVKAAPYWLD
VENPAGSKTVSVKVL
EKKAQLSKYKSLQQT
ILDITDVKVEDSGSY
EKSPLPQKRITNIIE
QAKIQEAKEKVQINV
KLKVEAVKIKKTLKN
KSALGFIKVAVTVMD
IKVKSEEKIKVVEKS
RTWAEAAKTVLEKYP
TVLHGNRKDNELIRV
VKQAEKAKDSVYKRS
TNGEHTSKATLELSP
DFACANNKICDELEE
LHSNPEPKTFTFDHV
TVARTMMKVMKLTTG
RGPKVDIKAPDVEGQ
ISLGEACKKVFLDCC
LGYHVERKERNGILW
PGPCPSVKVKEVSRD
KVTNQVSKQKYKEDY
FTCPHCAKVCKTAAA
SRIDALEKAGQKLID
NVDISAPKIEGEMQV
NALNVSNKLYREGWD
ALIRTLGKQLNVNRR
VTLVEAIKQVVKHLP
ISVEQDGKVKTLMEF
HQNTMEDKFIVCLNK
NLNSLCRKYHSAELE
KAAALACKKLKSLGL
PSVSTHIKVKSEEKI
DEEEVSDKGSDSEEE
VGPDGLMKLNGSALN
SSEHDVLKEAVLKKL
STYHIPTKAEASTSY
VTMDSAPKPFTDVSI
LVSDNNLKDYFERGR

GLRPILTKLYKEKGV
LQLWQRYKDYSKQCA
LGIENQHKISVPRAP
DCQRIELKQGYTFVI
TDTVSDVKYKEDLTW
EPPSFVTKPGSKDVL
EQWLDDCKRTFGAKE
QEVIGKLKGLCPSVA
ENGSLEIKMIRKEDQ
MEEIENLKKQHDLLK
LQYMTMAKLQKEFGP
FRRLQFNKHGVLRVE
NAFLEFSKEVDAQSS
RDHLAEAKEKLSILE
QLPLSRIKFTGKECE
WRESSLLKNLWVSQK
DLKTQAGKDDKGKRK
KNNGQFVKASASLKG
LQGPSDTKDLHSTKW
KKEPVTRKAGLLFGM
DSAEALLKKHEALMS
QTYGSTLKVTXXXXX
SFDFLAEKLQFYQRQ
SAPAPQCKALCRKPE
ATGLDEEKRARCDGK
KPENLSSKTRGSIPT
SCASNSVKFGVEMLG
AGPPTPCKPSSPGAD
DSRVSSAKLNNFSRL
ISPTNPTKLQITENG
QLLLEEIKSSKDQLK
LKNVSEEKMAAIVNQ
QKNSTLPKAIVSSRD
KQLEKMRKFLDEQAI
LFNWYNRKNCILADE
NDDISGYKENYTRWL
ITNYIIEKKEVGKDV
FKIFRETKENEIQDL
QRVRENQKNFSLLQY
SRLSTIWKGFINMQS
DPLVTVMKVEKAPQE
PELPLQTKDAADGEA
KRCKLLGKSSRKRQD
VSFSAGVKVRFLLGG
LENMQKLKLIRMCPY
LKKRKGRKPRWTKVV
VETICHRKEGGSSLI
KKETTVKKAVVVPAR
VFEYFNAKSKHSWRK
PSVENRPKIAAFLPA
CLYQTNSKLYESLTY
QKLVYMGKLKEKESF
AALRPLVKPKIVKKR
ALRAEVSKLEQQCQK
KQKLYHKKIFRTAML
IISDSQIKASEFLGY
FDTSAAYKSILRKKV
DELTTEIKELKETLE
EILMSLQKVLSGLGG
KHGFSVLKGQAELQM

NRVNHIVKKIETENE
TYVAQFLKHYPDIHN
YGVNGVGKILKKINK
IFRLQTEKDLNPQKT
SESELESKESQKDEK
SPSPAPEKKEKTPEL
EAPLPSPKLEGELQA
ASPDEGWKRCNAAAQ
KLKDDTEKREQKDEE
DEIRTSLKNLNQFLP
YGAVTYRKSKRGPKQ
KVLASYQKTRSAVIV
NTMMELRKCCNHPYL
KFRLEEQKVRRQEQE
YGVFMLHKKTVNQKT
FAPDPENKGTEVCMV
XXXXXXMKKGSQQKI
VPTQPLLKAHPKFSK
RISDAENKHILELSN
KQVTQESKEKKVSEN
TFYSQREKPGIFYQQ
ENYQTLPKSTRHPSG
DCEEAFLKNPEGKPR
RVFCSYQKKGFDIKW
TLTETRFKTGTTLKY
KQGDELAKLSSSFKA
GFNTSQGKLLRTILF
PIETERSKCDITGLL
HPTEEALKISVASEP
EIVECILKRGQKATL
PEDKKEEKEKPKKEV
GSDQERTKKKRRGDR
PENHNNAKEPDSEQQ
SELSQLYKEAQAELE
VANVVGQKTASIEIV
LSITVRTKKQELSEA
EFEQIMLKAMQEFTL
QEAMLFFKKSEQEKK
FTLQYLNKLSMKPEP
PRKAKEPKKAKEHKE
APECMALKADVVAKK
TSTPLSGKESVFFAE
EIMEHRFKTYQQFRR
YLFIQTDKTIYTPGS
LEAVRQEKQKETAKC
SEFSSFDKNDSRGQE
SLLTFIDKAMHGELQ
FREGQILKVFGDKDA
KERISAAKVAEPPQR
IKASDITKSSCKLTW
EVQVLDDKNVRRRFR
FHKLNIPKLDFSSQA
DAGLYTCKVSNDAGS
QTEETLTKVSATPGP
EDSEQELKGYACRHC
LNINPDRKEHNCREE
NFILLFYKVSTSSVV
LLDVNLAKFLAQDVP
FSTALYGKLLKLPTC
LDPESQRKRTVQNVL

KNSNGIPKLKYIPLK
SSAPSSPKLQHGSTE
KLEYIYAKQQALEAQ
DQLKLQEKDIQMWKV
RRVAQAQKALSDVAY
KESARDPKPEASRAS
DRMTEESKVEAELHA
FIRTRLEKEGIVFKS
YSSVSQRKYYSTKHF
KALLLEAKQKVPPVL
EANPLTWKQLLCDLV
IAEELSYKTAHEDIS
LQNTDAHKRAFHEIY
CKATLSEKLGGAEVA
KEETNIVKLLEKQYQ
CNSVHLCKQLVESGA
LITPEDRKYGTTNLH
LLDYQDHKGADSHAG
TECHARFKTPEDAQA
ATVNIQLKGTNEYVP
KNLDHVNKILKAKKL
ANQGCVRKVYLKDTA
LQQATPPKNYQPAAH
LCSQLLCKDPAERLG
QEEVIEVKVPAVHTK
KTKRISAKKRDVQQQ
CLTENVLKEIAKAGR
EDEETDQKCSLSSSE
LISLQEAKDICEEQV
LRNLVHSKHKMIAMG
IVCSSEIKRQVVETM
EPPRFVSKLNSLTVV
NCRCEISKSLCCRNL
STGGEHKKSDRKEEP
AETLNQSKERNRFSL
SEPSELIKVLGEKPE
VAGGAISKPLTDQTV
AFKNIASKIANELKL
PVLQEEIKESQDPQH
RSLVEQHKRERKELE
VPGPDAQKTETPSVS
QSISENEKLQKLKER
DLKGTLGKLLDERLV
GKTGNTEKFGRFRQC
KWIQLLEKIEEALKV
VTAEPEPKIPDVAPI
VVRGWLHKQDSSGMR
TEVELFHKDIEAFKE
LTKQIQEKQGQLSEL
VPTFTIPKLYQLQVP
MGLKGPLKTPIAAGH
VVYCEELKCWCRAIV
SSEEPTEKEPPGQLQ
HLRTRVQKVQVKEEP
GRIETDTKVLEILYE
LACQENCKKTLTLIE
IGTQTNLKCRRGIEN
NSKVKHKKKHYKPSS
EPIRNSVKERESERN
SVTRSTFKFTRLTEG

TEDLEDAKKELAIRL
PDSSPSRKPPPISKK
IPMAPSCKLLFEVHN
VIIENVSKSTSTSEP
GVNLIRSKVEDPAVI
PICRGIIKGTVRTFL
CTTHLYIKAPAKFVK
LVPGNIFKSSGLTDG
RAENLRFKEEGVRDV
EIEVSQLKEQSVLEF
IENIDFNKSGSSTAS
LEDEAARKAKQGKEN
LVAEFPEKEAQLSLV
EISLNHNKIEQIIAQ
QSLKDLEKEFVDFWE
PLGLIMLKTTSEELA
GTAPTTLKEPAPTTP
VSVNVSSKEFLQTES
LSMGTRRKVLVLGSG
LKEEIKKKKGIKEEV
EFWDVIQKHCLEAHQ
NVLLTCCKDNVCRLW
TTASNYNKSESQESL
AKRELESKLQRLQAQ
TIAEQESKLNEAHKS
LKQQAKEKQAQLAQT
QLSLLPNKDLFIWDL
ATQLEQMKEDYSYIM
HQPVSFYKLKLTNNT
VRQVEALKAENSHLR
WLQDVDDKYDRMKTC
ALGMHESKYLILPVH
GAVVEFVKELQDIEV
CEIPVEVKPKARLLR
RLGSPSGKTGTCRIS
QDLLHTTKHQDVLLS
YVHTNGPKKKKVTLH
LHEFDSLKDPEVNEF
LEAENQIKKCDIRNK
KQLTDNLKEKALSFM
KEIKKLPKDAKKSST
TKMDANDKKIQSLEK
LAHWSPAKLLLQMDS
LEELQDLKGVWSELS
LEEHMFHKLIEAGET
AVAVARQKDSGGSGR
EHLCGCWKQCPSCGA
SELHESWKYNMSFIN
GFYVVCAKNRFGIDQ
SHNVQQLKSRILMTA
KEAYEKSKGKHVGFR
KLRLMEQKFQQVDEW
IFHGVMGKDEREGNS
NMHSARFKNGKQIRK
DKVDGLQKQTESSSD
HLKQHCNKVLLRVRM
NSDQHYSKESNYFYS
TGFALNLKPLAPAGI
DGNHLYLKIKNAMPE
QGLPGPEKLPGSLRK

PDFERVNKRLCPTTS
NKLPLPSKSVDWTHF
KFTMVKNKALRKGFP
RQQSSSSKGDSPELK
KEFSDLEKKFKDKSQ
IGSLDVEKCKRATEI
ISLNDVSKAEGILLL
VNLHRIEKDVQRCDR
AQTADPIKVAEVPQP
FPLWESTKVVPLSKF
AGFEDEAKTSARDAA
CVSIEGYKTPYTDVN
AFEHFAQKGPTWKEP
LSPDSFIKDNYGLNQ
SGEQVDGKGQAGSVS
LEEIRQAKAQRKQLH
VVEVFLEKFTTEWDN
RANKKKQKVFQHNEL
LETGDRVKMKTLSAY
AEREQVIKLGTTNYD
DYCLDIIKKFEVSEE
DMDEAIEKGEAEQSE
SSLSDDRKWIIFDGP
YCTSPDFKMGGASDV
FGVPSEPKNARVTKV
AMEESPRKKKKRKQE
WELYIPPKQNKSVLV
DSTADDSKDSVAQGT
VIQFELGKQKNIMPL
GGLRRAVKVQCTWSE
ENYIEELKKQGFLLR
SFLLIASKSVNMKKE
TFRWTSLKHSRSEPQ
IQESSPTKNNIAVSS
KEAVYEKKQAVHKEK
FLAPVFTKDEQKHRR
NQKRAELKLVVDRLQ
HFQTHLAKLLETQEQ
QQRRYLEKRSKHSKK
GRTTDLSKGLQSPRG
RWIREIQKVTKLDRD
KDKEEHGKIKREELD
CSGILTVKEPPSFLV
QKQFRLFKTAALVIQ
SNANMLRKGPAEDMS
KSEWMDLKKEYLALQ
TICSAYLKINSKGEG
DFPKVRAKEQTFMFP
IYTYFVYKTRNMDMK
KFAECLEKKVDMSKV
SIKAFAEKLSVKLKE
IANITALKDQLLAAK
VMVKAYLKKYDPEVQ
EKSLKRIKDTNKDIS
NDLSTENKLKVLKHD
KMVAWFEKSKDIIQS
KYRAALGKLPQQAND
FHCNILMKDDKDPQC
PDHMVLGKRLKGAFK
NLSVTSFKKIVYREY

VVENSEGKQEAEFTL
DTCVCTVKLKEPPRF
VQAENLVKEASEIPL
GVNSLEVKFRTRSEN
ADLGVIYKIKLRHDN
IQEVEYVKEEAKMVY
FGNAIEGKSNTVASL
SICQARAKELEDSLQ
ISQLTEEKNDLRNMV
IPTIQWIKGDQELSN
GLSLEDSKKLTASPS
LLDSPLNKEVVKVSA
GSTFPDVKPDLEDVD
RESVNLGKSEGFKAG
VVDSGLHKEERYDLK
DTVRSSFKPGLEARL
PWMRLFFKIKPLVKS
SSHEAEEKLSEHKAL
KLKVKDNKKNRTKKK
GGGGGMAKASAAELK
TQTPVSNKKPVFDLK
DFLIQTFKVFQKNQV
NGCMNRFKNALNSCD
NIELVDLKPLPTARR
RVQEAEDKWRGAQAL
SDLHVNAKGPQVSGE
LYTQLEAKKAAIKPL
FLENEDTKFSLSDDQ
CLIHPRIKVGNEYVT
VEVPTVTKRERKIPE
SSSEDSEKVIAGLYQ
PVSMDICKQEEKLGF
MACTWSVKEVASYQP
AQNRARAKEFHKKTL
GQPDEELKPKKKVFE
GEVIALDKIFNKVEK
TLEERLDKACEPGVD
CGRWLSLKKEDGRLE
RDAEELEKWIQEKLQ
INLAGVGKPSLPSEP
GLGKVGSKGREAPLM
RVTYSPRKEKALKIY
ERFDVGPKEKPLEEP
NEVINSPKGKKRKVE
RYLNEEYKKKMSYMI
AVYTCEAKNDYGVAT
QSSGIGSKAGVPSKS
QMIEKRSKLGESQTL
SDPTPDGKLFATQYC
RFTMEVPKKLTEWFK
AGAGETLKQECDSLG
DAHEDSFKSADESGQ
NVFNNLAKGFSEGTA
EPHIQPTKSSSRQNI
EPQLNRLKEKAQQLW
MPIVDKLKEALKPGR
LDVEIQDKPDKPTGP
IVSALWEKWLSLLEA
PGLELMEKEVEPEGS
EILINNFKYIFSHLV

NIQNWVYKLSTSSDI
TTMDTNGKPLYILTY
SDAGIAGKVERNIPE
EEAGGSVKEEAGPEV
RLGVSLSKGLHHKAV
ELALARMKDLGIQCR
ELLLGHEKAPAFRDY
EVQISTEKQQKNSTL
KHRLRELKLQVKAFA
RMIVRCRKQDFPLVK
LGSGTYFKKQFSDPV
EWIVESIKAGRLLSY
EHHPRVAKYAMENHS
EEEDEEEKEEDEEET
KTVPLTAKEERGMGA
GDPIPNVKWTKGKWR
HLTEETLKVTAIPEP
LEAMLRNKELKLQES
ELTQEALKVSAVPQP
RHLIEDLKFRQKVNL
EIAPEEEKPVPVAEE
LPEPPVLKVLSSLKP
SKGIAFVKFARSSQA
NTMDVFVKEPSFDWK
VTVIGIPKPKIQWFF
YIAETDEKKINRIRQ
EQAEIQIKEESKTAV
LQTGACYKSLPSRNG
WRADINSKKVLREAG
EKLSILEKEDETEVQ
TKIIEEEKKTLELKD
LLAPHRQKWVERIRY
RWKPMSVKHTEKLER
KNTGELKKSKVLSHH
ARCLRVGKLNIDRLY
VRSSGYGKPSSPLKM
SAIIIQRKWRAILPA
TVIQSAYKSLIQHLE
RLAEETEKLRAENVF
ETREDRTKRLFRHYT
PMIPQSGKQHLLDFF
HERSRRRKHKRRPKV
EFPLPDEKTKKRIFQ
TCASAVQKTTFRVTR
ELDQDLAKAYLTVLA
EQNHLYLKTKAAVVT
GLLGASFKSTGSSVP
RLSSWCLKKPGHVAE
IISSLTPKHLQAGMQ
EFSDDIVKIIQAAIN
YLDSTFTKRDPRGEY
IVSNEAGKASCTTHL
NDNAPIFKEDPFISE
ENIQTLLKSKEQEVN
PVLIPEPKIRAVSPH
DRVQAIVKGTKQRLV
STGPITAKDEVELPR
HRDADETKEWIEEKN
LEPDLAEKNPPLFAQ
ENIHSRMKTVKLPKK

VPGFCLPKKGVTSIL
VKAGEVLKINADIAG
PGVQGDVKGPQVALK
TKHLETSKQLAAAFQ
PTSYQAPKHFHIPAG
SVHSREAKESSAAQA
ERNIEAGKDDGLTDA
CSISQELKLKSFTYE
SPQRLCSKHMPQLHS
VPVPVQKKEAPPAKV
ALKMSPYKDILETHL
VSRYGELKSVPTTQC
QPSLRAAKEHAMPKD
AKDLEQHKIKQEEIL
RTLTYTAKPVGGIGG
LKKLEEEKAEAAEAA
MDAENMRKELAELES
SPLKEDAKAPEKEIP
NVSSRGGKKFLPVLK
EEEVTVVKGQPLYLS
QEERAFLKVDPELVV
DSLAAHVKNLLQCES
KEVMAAYKGKRRSEA
GLVAQLQKTVKELQT
RLHTRQMKRTKCADI
NGFSAMAKTVGLPTA
QRLLEAQKRLEKLTT
KAKAQLCKIEKSKSL
LPCLSLRKLPPRSEI
QKLAELEKILESNKE
ADSLYVEKIDVGEAE
XXXXXXMKWVTFISL
VFRRDAHKSEVAHRF
DLGEENFKALVLIAF
CPFEDHVKLVNEVTE
ESAENCDKSLHTLFG
EMADCCAKQEPERNE
DNEETFLKKYLYEIA
PELLFFAKRYKAAFT
ECCQAADKAACLLPK
DELRDEGKASSAKQR
LKCASLQKFGERAFK
RLSQRFPKAEFAEVS
KLVTDLTKVHTECCH
DDRADLAKYICENQD
NQDSISSKLKECCEK
CEKPLLEKSHCIAEV
AADFVESKDVCKNYA
VLLLRLAKTYETTLE
DPHECYAKVFDEFKP
EEPQNLIKQNCELFE
FEQLGEYKFQNALLV
ALLVRYTKKVPQVST
EVSRNLGKVGSKCCK
QLCVLHEKTPVSDRV
VDETYVPKEFNAETF
DICTLSEKERQIKKQ
TALVELVKHKPATK
KATKEQLKAVMDDFA
DFAAFVEKCCKADDK

TCFAEEGKKLVAASQ
NECFLQHKDDNPNLP
XXXXXMGKGDPKKPR
LDSEAFQKRKQMQEI
PQLSEYCKMPLKMWH
IILDTLEKCLAGQPK
KAQAAEMKAVHEAEG
KVSTEHNKECLINIS
MHLIQTGKGEAIRIR
QELISLAKSRYSCRD
VVFVQTDKPVYKPGQ
ALKLQKGKQYLMWGL
TVPETGRKDTVVKVL
ALNDHFVKLISWYDN
AEFAEITKLATDLTK
SIGLGYHKDLQTRAT
NCLAPLAKVIHDHFG
ALVGHLLKGYRHPSP
GRVHESVKRSNCYMV
EIIQDISKDVVDESN
VTLGCLVKGYFPEPV
VFANMQMKLSQSRPI
ADGNFDPKPINTMNF
VVGYYCLKVPLVVFK
NRTWQDYKRGFGSLN
AEGSPQYKKWYFELI
GIISIPLKLPSLNKE
KQLRKIVKHEIMSVK
DGFAESTKRKAAVWP
LYPMLFNKLKNTISK
TRHMEAVKTQFLEQA
VVAASEAKEDSPAQT
ATAPEDDKAPAEPPE
NNLHLQEKSDDPAVK
EENGMAWKEILNLLY
QHSPGLDKENVELSP
TALAELVKHKPKATE
TVRKQVTKSYKMADE
EFWANLEKETDWLRN
DVAVWFSKRLPTFVN
RFAGPFSKQTQIPDY
CAIYTYGKPVPGHVK
DLVPGSFKSQLQEGP
AAVRQRMKCSSMQRF
GELLITNKGSELAFA
WLTAYVVKVFSLAAN
ARYEREMKTYIPPKG
LSGFIPLKPTVKKLE
SVSRVELKPGDNLNV
RVTAILDKLITMTIN
NMVIADVKMLSGFIP
KCCAADDKEACFAVE
ISCADAEKEIMRLGT
ANHVLFTKEEHMRPF
SVRFVCQKGFVLRGS
VSREINLKDYEGQQK
QMLFYICKKLTSHQM
FPNNYWDKFVKRKVI
GGLVEGAKNIRVTKI
SSPCSSDKQNVXXXX

LEEPDLEKVVTYLAG
RYNMPLEKQQPAFAL
QYDSTHGKFHGTVKA
KEYDPDGKGIISRKE
NLLQMTEKFFHAIIS
DLLVHCGKLCALVYK
PSFSSETKSACKEED
LMVCNHEKVGLQIRT
VERSYAVKSGKWYFE
YAVYSLSKSYIYLDT
MVLSAADKTNIKNCW
LLKETAFKFKALKKV
GMRDIPMKYSCQRRA
AVINSLEKAFWNWVE
GMGVPEIKYGDSVCF
FAYKFLRKILKYVDS
NSLFDFQKNNKDSNS
RGNFLEIKNKFLARR
SLSHSPGKXXXXXXX
NAFERQNKAEGLGMV
AADFVEDKEVCKNYA
SVLGMPDKVEDMCPD
TVKAENGKLVINGKA
LFIVSISKTLAANEP
LQMISASKGEMSPMV
PPVEPNTKVFPAVFL
DQTEQWEKFGLEKRQ
EIPLQILKTKALSML
ERAATVTKKTYEIWH
FQVELPRKAFITNFS
EISCWTVKIXXXXXX
TDQHGLAKFSIDTSS
QAELLPSKSVSLIIL
NTLCDEFKADEKKFW
PTLSQQRKSQNEQDT
YINVDCAKLKRLLKE
SLLNGNSKYMVLVPS
EAMQAYPKQDRPLPS
TNVEMILKFFDMFLK
LLALLLLKDFDSVPP
VTVQDFLKKQVLTSE
MSFSAGVKNWVDHMM
GVELMEAKSQLFLKY
ALLDERTKKSNRDSL
ESHQHLSKESMAGNK
SPEALVGKSLYVSVT
MAPSLTFKEKVTSLK
PMTVICQKDLSWSML
KVTSLKFKEKPTDLE
EILKSLDKEAIREDN
VTYPGLSKRRLKPEA
LQLSGPDKVTISLLS
VAFNFFRKFYNKSED
VIPELNGKLTGMAFR
VDESNINKKLFLDSL
TVYVDAVKDSGRDYV
RWPSHFQKEAFVKPG
VREDMETKCFICGIG

Supporting Information S2: The benchmark dataset $\mathbb{S}_\xi(\mathrm{P})$ used to train and test the model for predicting the possibility of carbonylation at Pro site. It contains 126 positive samples and 792 negative samples. None of the sequences included has ≥ 30% pairwise sequence identity with any other in a same subset. See the main text for further explanation.

(1) List of the 126 peptide samples in the positive subset $\mathbb{S}_\xi^+(\mathrm{P})$

GKWELYIPPKQNKSV
PLPRKRPPVTQAAGA
QRPARRGPPPLARAV
ASKLQVLPQKASERL
GYKVLIQPSNRRAIH
KVSAAPGPADQKTET
PASKDTRPKEKRLVN
GRKSTEFPRKIREQE
KQRAKKKPNPVEEEP
YYYWKKTPEAASSRA
QRNRETAPRTIFQRV
KSLQAKFPSNLKVSI
NSRTMLLPLLRRFPE
EPQKQVQPQVQPQAH
IAKQSLRPFCTVCNR
ERKQNLRPMPKKYHS
SPVQKANPGTLAAEI
PTLPKRVPRAGEARN
VVRLSCLPAFKDLIA
KERKMAVPMPSKRRS
LKKAGRIPEQILGKV
ASLKRLKPHVPLGRN
STKEKAGPKGSKVSE
VGIPDKIPFKRPCTY
YQDLDGLPCIVILTG
KTVMGAAPELKVRLE
AGRLFLIPATIQDKL
AKARVTKPKTAKPKK
PNSRFSAPSCGSSEK
VFKLLECPHLNVRKA
CSGKKVEPSALACRS
KEKQPTMPILKNEIK
RLKNEKEPMVLKLKD
STIPLSHPKIPRCQE
VAKAVECPPPRTTAR
GSETPDGPLSPGKME
KQATDVKPKAAKEKT
KKEEKEKPKKEVAKK
EVKKETPPKEVKKEV
KKPAALKPKVPKKEE
KESLKVGPLDSVTYL
LFNRIGDPSVDEDAQ
LGGKLKGPDVKLPDM
KIPKMKMPKFSMPSL
VDLHLKGPKVKGDAD
NMPKISMPEIDLNLK
KKSKIKMPKFNFSKP
PKGRPDRPRDLELTD
DSKKEEAPKKEAPKP
KEEKAKKPEEKPKTE
ADGRGMLPKRAKAPG

VVRPEEIPPVPENRF
RKRVSLEPHQGPGTP
KKATSCFPRPMTPRD
FPENKKQPYYYPPFD
STLERVLPRVRFTRH
VVERRRRPRDSPTLS
YKPKRGRPKSKEMPQ
IPREKKPPRPPKKKY
KIHRNRYPVCTKPYA
ERSPGGRPIHKRKRQ
KGSDEKKPFKEKGKT
RALGVQTPSIRKSLV
ELIEELHPIIKEALE
NPKEEKKPKEKKKKG
MDPSAPQPRAETSGK
ILDLCAAPGGKTTHI
KHTFSVLPGDCQQRL
RVPRRASPLRTSRSR
KKKGRPDPYAYIPLN
LLKTHFLPLMEKLKK
KIKKAASPSPQSVRR
KKAKSPTPSPSPPRN
KKLLVLNPIKRGSLE
RRRAPPPPKRAPSTT
LPGPEKLPGSLRKGI
PDMLKNAPMLKSMKN
LMEKEVEPEGSKRTD
KLTRCLKPFENSRLR
SYRTVEKPPKFTEKG
EEIRSHRPTLDALRE
KVSRTVSPKSFAKAF
KSTLAIDPATSKEIP
KEPFKAAPHTIHPPC
KVPIKQVPGGVKQLE
PAKVPEAPKKIVPEK
PEEKVPVPIPKKLKP
PEVKPKVPVPAPVPE
RPIKPPGPPINPKLK
PFVVPDAPKAPEVTT
TQEGRQQPVPAPQQK
ARKALCDPLEEVREA
LRLLKPQPNIIPTVK
KKPLKKKPTPVLLPQ
VIRSLKRPPPADAPT
PRKRTHAPSALSPPR
KQDKLKSPFKFSDSA
GNGIISSPLTGKRQD
IREATAAPHGKRKRK
LLVNEQEPCKFLLDA
KTLGIKLPFLVMIIK
ESKKNELPDGLNKKR
LKLYRLLPMRAAKRP
ASQRCRDPPSNPVAA
RRAHTANPLHRCRCG
AVETEPEPELRQQTE
VYDAYLAPNNLKPVV
GLLYCDLPEPRKPLE
CLDNTRKPVDEYKDC
EFQLFSSPHGKDLLF
FFSEGCAPGSKKDSS

QNTGGKNPDPWAKNL
KTPKYFKPGMPFDLM
RNGQWSEPPKCLHPC
EFLRACTPGASLRKG
SHFRHGIPFFVKVRL
ENVGTPCPVSAFPSS
LSHKDDSPDLPKLKP
DKGACLLPKIETMRE
ARLSQKFPKAEFVEV
KVPQVSTPTLVEVSR
GTRCCTKPESERMPC
CVLHEKTPVSEKVTK
ESLVNRRPCFSALTP
VELLKHKPKATEEQL
KRVIISAPSADAPMF

(2) List of the 792 peptide samples in the negative subset $\mathbb{S}_{\xi}^{-}(\mathrm{P})$

LCGMLSLPWIYSHSD
SGGSYVYPPVQEFPL
YIVPDYMPVVYDKLP
TTAGATQPAAPTPTA
QMANNFTPPSATPQG
KGSETYSPNTAYGVD
STNMGILPSVTMYNF
GKSTDFAPIKEDFGQ
HSWQEQAPNQPPGPT
REKLKAKPLLLQLPI
SSFIKRQPLVIARLA
VLGEYYTPALKCEVN
LRRVAGTPCGVFGQR
ALEHEYEPYSRLKSD
VIINQLQPFAEICND
KSDDPVVPSLFKQKT
SDFDFSAPKINLEIE
VGGTIKSPKNNEKNF
TGRQIFQPLHALRNA
YLSTEDVPLARMLVP
PEKEAKSPVKEEAKS
SHGEKELPQWLREDE
SPTASQAPNQPQAPT
LEKQFVKPDGENRAR
RFMEIESPHINENYI
RSLWRLEPLRISWSG
EEYIALYPYSSVEPG
DALKSREPQAKPQLD
AIIALFTPTTDPEAE
SSALGEKPITFYRQA
PIPKKLKPPPPKVPE
SLSSAGRPGPSEGGD
RLCNDSLPPGTVKLX
AELEIANPPELQKHL
NIVGIGKPSKVSECY
RDIPAMLPAARLPTT
VMTLKWTPNQLMNGS
KPSKSTEPILIKDPI
AELLANMPDPTQDEV
IKLNYALPPPLHQTE
VQGLGVMPPKAGQTI
ISQNESRPHFLIELF

PLVDHTSPSSDSLLF
SSTVNGVPSRSPRLV
TPEDQDLPPCPEDIA
RCISSALPRRRPPVS
SSISDHTPWLMPPTI
SLSHSACPTPNPLSR
MVVCWGHPDSDGGSE
SESSFGKPXXXXXXX
RQFDNLKPSGGYITG
TVLDANDPPIFTLNI
SWSRISNPSAFSIVP
SSIALGDPHIPTSPE
FNQSLKSPSRLGCPG
PPLLASMPAQLPPRD
IAQDPKQPPDPPVDV
ECHISGYPSPTVTWY
KASVVTLPVYLNFTR
NTSSDCRPSEESELL
WVKENKVPCLECNYK
SWLKDGLPLKESEFV
EEVDCMDPTCSGRGV
AESPEVLPHIEKELS
SCRSEGTPAWYMHGE
VAFQLHLPLSQRACS
DRFCLSSPTEALKMG
QVRVVAQPSLPAVPQ
WIRDALRPPLQNINS
HKESFLAPVFTKDEQ
AAVQKAIPMYKIATK
WGMESKIPVEDNKRF
AQNPVDAPGRPEVTD
SEDSVQVPRNLVGKV
YPDIGWNPCGGERYL
IGSKAGVPSKSSGSA
SLEPHQGPGTPESKK
LWGCHSAPHGPGLIY
EIGDGFLPVCSLGPG
GAETLTFPCDRWLAT
LKASVCKPFLFLLKK
ESGKIQEPFSAMSKS
GTQVSQRPGAQALKV
KEQQKDSPVFCRFFH
QSRPTGVPTPTSVLS
EMFGVWEPLLEPLEI
YTEVANIPVARPGRR
SRLDLDLPGCQAEPP
YLNKKGNPKKFAGLA
AIESLVKPQAKKKAG
ENIDGILPLHDAVAN
GKLYIEAPTFDLQGS
VQIKKMEPDEQLRND
KRKKKKRPEDTAASA
SERLQRAPLKSVGPD
DPRQLGVPVIARDIE
ELLSTTHPANKASLT
SVTVTTIPASQAMNP
LSAHTILPGSKTREP
ALKISVAPGLADQKT
DRGEIIDPFVEVEII
HKDLIKKPTISTAVG

DRETILDPNLQATLX
DGAVPAAPASADAAR
VLAERKSPEISERIV
SPHPPLQPLTGSAGQ
FLPASTAPMQGKRKS
LNNSSSSPQRSVDQR
KNALENYPNFRSVVD
ERLALAGPQLRPEMS
LKSQTQHPWEKLLNL
NANQNASPNVPGKRG
SLEMGILPREIRKLV
EATPTAAPPTLPPTT
EKHTHHAPLSPRTFH
QYRKVLMPKLKAKPI
ALSELDVPFKVKAGQ
DAEVYNVPLDSQSDD
DKLTLKIPWKNLYGE
LSPTLNTPAPVAMPA
TRSLGEEPVGGLGSL
EEQVNSLPGSITKAG
KVVIGFVPLAEIMGY
TYIELMRPVSELIRS
PDVEAHGPEWNLKMP
YFPNRKFPSSSVAFK
EGSRLSLPRLIDMSA
PRLTPVRPAAASPIV
PQPGLVVPVPTVRPL
LLVTAMGPPGGGRND
KIEKELKPYGSSAIN
ENLELTNPQEFGSSW
AVDKRFLPDDHGKNA
PSAFSIVPRRAAKSS
RLLSLSKPILPQQDN
IKRQCVWPFIVMMDD
ANLHPAKPKDFSAFI
TQITTESPEKTLFSS
SDCCGQKPTGPGGPL
ACVTACEPPKTCQDG
TMATRTSPRLAAQKL
ALTALTTPIQTAALQ
KLQSILKPMMLRRLK
RKKMNLKPIMRMNGN
EKAFEGSPARELDVP
GSSGAGVPGGAAAAS
SKQKKVAPRPSIPVK
LPGSVLSPPPPPPLP
GDSGGLIPGKSLVFA
NKEEGARPGTLLGTF
RRTEGQYPSICPRLE
HEAIILKPEAIEMYI
HPFASFHPGLNPLER
PDGLKLYPTLVIRGT
PQRPASEPHVVPKAV
EIPKKKVPEERKPVP
RLFNDSSPVVLEESW
GDRSGIFPSNYVKPK
QAAIIQIPKPASNRT
EKRDYREPFFRKKDR
FTNTSLHPQRMKALA
PQFSSLQPPCFPPVQ

ACRCSIFPDLSFVTF
LMDYADLPYQIEDIF
FIDDINMPVINEWGD
GTLVLCLPQIKIISA
ISTSEAAPYAPPSGL
TPSWLGLPNNAERVL
DLEDESTPIVKLGDA
EDVVASLPLCHAALR
RTHTGERPFQCHSCG
GNLHLLSPGNSARLT
EAIIDVRPASTRFLP
EMSDSNSPPIPYSQK
DSVGVMSPPLSRSSV
PRSVSRSPIRMSPAR
NMVNGMGPMGTEGLF
TNTSVLGPMGGGLTL
TQGSEKCPQKTTRRD
EESQHQQPDDSNNIA
FAGTEIDPENEELML
KVTIKDKPAVAPATK
NHDESLLPESLESMM
QHLPSVSPSVSDAFL
LNRDLSTPGLEKDSG
CEELRAAPRQGPAPA
PSKEEPSPVKAEVAE
NSSRQERPPVKPFIL
GTEEKKKPSDFKKKV
GGESSASPGEPQRTL
QADRKILPFTSMRHQ
RARTDEVPAGGSRSE
RFEMEGLPVDESDSC
LLSQLGQPSIFDTQK
FDAEDGEPQTCKSMK
AASLAELPLPTPPPA
ISDTTLLPDLSQISP
NRFTMEVPKKLTEWF
TPESSSLPVALQTPT
ITESQSSPPPVIDLI
PLLPSSEPPPAPPLD
EMVSGFGPIYNYKDT
VQVLKYEPFLDCALS
LGRASVVPLPYERLL
KKTKGDNPNSHPEPA
ARNLFSVPGAPDKPT
AKVMELLPTHAFSTL
HAVAWGTPGFLTVML
TTSQDEAPREELAVQ
SYQEAQLPALPESVP
IVEREMAPDFELDAE
ATLEEGNPTDEVPST
LLKPEADPRPCERAP
PKKVAKSPAKAKAVK
SRRRPRAPKEKAQPL
IPMLYVVPRPGKAAF
PMNPIPAPLPPDIPS
GNDSDEEPPAAAPTP
DKVVIQDPYRLPGPP
FAKSDNQPSTEKAME
IAKEKRTPAPEPEPC
SAKITSLPPPPPTLF

LHLQRINPTTVKMKS
LDIKAKAPKVKMPDV
ESGDAPRPPPAATPP
VDLHRFHPEPYGLED
RRMQQAAPTDPDLPP
KAVSSADPRAPGESP
TIELEWEPPAFNGGG
XXMNTSIPYQQNPYN
GALMTLGPSVVRYHL
VTFKANRPFLVFIRE
VKVAQRSPVDSGTIL
PMKPLLLPQPEVLSP
NLVLKRVPSPVLIKK
MDDLISSPGKNKSGR
PRESACLPEKLKEKE
RAWGCAGPCGRAVFL
NWRQPELPEVIAMLG
MDRDCRMPMGLSTGI
TVENASKPDFTKNSQ
RTDLYFMPLAGSKLA
DRGLQLVPVWDIILS
NALNSCDPWVQAKCY
TNMGIIIPDFARSGV
SKTIYTAPLDMLQVT
RTPPRRMPPPPRHRR
XXXXXMHPPKTPSGA
YYRQSEVPDSVYQHL
EKDDSVRPNMTLKAM
ECLGAAVPARLRKVA
RSLQKDFPIQILSID
VDSDVAKPNQACIST
SLKKAKKPPLPPSRT
EKFRIELPDLGRFYK
AARISTSPIRSVRSP
AFPDGHLPEEALKVS
ETYTEEDPEGAMSVV
ALDDAEEPESPPPPP
ECTTIEHPFCMYDAD
GAAGSMSPSRVEANV
EDVQVLIPFKGRPPP
EKVSEVLPIQKNDEE
LKFSSMAPDLDRLNE
HPQRPPSPHPPPHPS
NRLCFLEPTSNAQNV
TDEILLNPNNKWFKP
GKLSNWEPKDDAMSE
GSGTDMEPSLYRCSN
FKSDGIIPFAHAIWH
DMLKRVEPLRNELQK
FGYIKIIPFYILNAT
GESKQHIPEKKNEVH
ATLSVLEPATIVEKP
LQGGNKSPVTLTAYI
DIIQVTVPDPSPTSE
ALGNQSTPAPPTGEV
FTNFMRSPACDIFNP
PDVSMVDPEALAIEQ
TSLFSNKPFLKLGAV
LLCGSDLPLHKMAIQ
CTVLTLEPNSQVQQR

YNGKLTGPAAVELKR
GDASIAAPFTSKLSS
IRDWLVKPIRDQHVK
SQVFIDHPVHLKFNV
AEDVSGMPALCSPIS
NHARLRTPPPPLSHA
LSHTLGDPIKIRAWQ
FQELSQTPGHTEELA
LGRPPSAPADKDGSK
GFLSYLGPFNQIFRN
ESQGEVQPNFSTSSE
LMLTAGLPELTSVKD
RPGPHSVPSQAPRLE
ILDEFYGPEKSLQPI
KIINFKTPEDARWIL
DREDQEAPDSDAEVD
KQNGIPHPRGSTAIF
AGQVAVLPEVQVTQN
LAELLRGPCGSFDVR
KAKKRRLPRGFPPSA
LQRAFPAPAACQCHC
VTRQLYEPLVMQLIH
EWFLRTFPDPTSWYS
NPKAITAPQMFGRLD
KLNQHDSPRIKALEK
MMLCSECPTVFVDAE
ILKKYGIPFSRITQE
EKTAAPSPSLLYKST
AECWRSRPEERPTFE
TLTYTAKPVGGIGGG
VTSSQVQPVTIQQQV
CQTSTVFPLKKKVSI
RTVKDSTPSSLDSDP
QVTDANDPPAFHPQS
LADTDLSPMDGISTC
EADASGLPHKLGEED
KKIIKEQPQPSGKQE
DCFSEEAPDATKHKL
SGQRSASPSVPGPTK
KTVKLKSPVLSNTTT
QKSLSLSPGKXXXXX
FVASGMGPSASSHGS
LGCTAATPPSKDPRQ
QWIRNLVPEFGVSSS
MRKFLSDPQVHTVLV
DRSLESNPEQLQAMR
VASSEAKPAATIRIV
DLQVRACPFLPVKRK
ESVHGTAPDIAGKDM
XXXXXMVPGEENQLV
FLRMSKVPTDLAVEE
DFLKRRLPKLSKSTA
QECKTKHPHHLSLKE
VLKFSCKPGFTIVGP
RLEVELYPSEVVEIQ
TTKSSAVPPGLPVYL
QLYDDSFPMEIRQYL
KKDTKKYPESTDTES
SHGDVIRPLRKQVEL
ESGLMGEPQPQGPPS

GHETEEFPDEEQLRR
GDSRGPSPSYLGGPR
ESRYQTLPGRGLSGS
DNANSRLPEDTTSVL
KAAYLSDPRAPPCEY
IGFLDNWPLLEQWFS
LFQKKIDPVTMDPEK
SVAQTGGPPEADGLV
EMEVCKLPRLSLNGV
SVQSSHFPSGSYSVR
ITEVWGIPSPIDTVF
SAQDMHVPVPKQLAH
SKDIPLSPPAQKNDP
YRGVKVMPFSTACNT
YQEKLYRPPPVLDAL
PAGPTESPASKGVTA
WVRCNKMPVKDTTYR
EKECAPTPAPVTRAK
RHAVKGDPQSPGRHW
TASLLAIPHTPESSS
VEAFFYEPYQGAIQG
FMAVKNWPWMRLFFK
ENYVQETPLVLSRCS
MTISWHEPLSDGGSP
SAEKNQRPRKQTSAP
FAASSTVPHCEQSCR
VATPQVSPNTVKRAG
EEDLLDDPSLEGMCG
ASYESESPENQTEKE
WQMWTLLPGFCTRPT
RQLDRLLPKLARGSL
GKKVRKMPVSYLGSK
ICHEISEPRLQNYDE
IVDVLDVPGPVGTPF
KKSRFWEPNKVSSNS
QRFCIGHPTQEMTFG
RARSLKFPGLISGCT
LIKEQLEPPEIDMKN
HSDSGYEPSFGKSSR
GLAWARAPQPKAWVS
ASSVETKPGASKVAT
LIRICSKPVVLPKGP
AKKEDKTPIKKEEKP
GKASIKDPHFLNFNK
MTMMSHIPSVMKAHG
ISELTGTPFDVESDS
DYALLTLPAEFSSQL
GATAPPAPAEPTPPP
EIYGKWSPLILNPSY
EGKSLRFPLALEEKQ
NRFGVSKPLESAPII
PQLSVCNPHSGKEYF
SNRKGETPLKVANSP
YGIWKLIPNGQYEFR
GGAEEERPGTPELAP
APLNVVPPEVPSEEL
ELVQAMFPKLNNQER
GTELSLNPTCAVFIT
KVFEEAKPNSELCCK
VTSAISLPNIRKPDG

CDEDHQTPRDGETSH
VNYCECNPCFNGGSC
AFYQQTLPNSHLTEE
VYIESRRPNTPYFIC
YLAQQIHPVVARICE
VEDKSDPPEGSEEAA
XXXMTAEPMSESKLN
KSVHLQKPKPDKKKF
ADSVSLLPAAPEGSR
GCEGSSKPFFYNRQD
RRREQRDPTVHDDVL
LDGEEEEPEEETTLG
SGIRAGYPLSERQQV
MKRESQNPDQKDETN
NKVGASDPSDSSDPQ
MKVVTLKPTVLEHTV
ITPKSDVPIQAPHFK
ENLKIDRPEDAGEKE
ISDHSETPNMELSCR
SCSENQEPGYCTVSN
SFIKQNNPKFSAVQD
PEDDGGSPITNYVIE
TLVSGSLPFDGQNLK
LARYSYNPFEGPNEN
PASTRFLPQGTRIAA
LHEIYFLPDHPELKK
LNDRMTFPEELDMST
GRIFVFEPPPGVKAN
QSKLYHLPPPTVGPH
IKNSNGIPKLKYIPL
FLEVDEYPEHIKNLV
QETAEGIPPGSQDSS
XXXXMSEPGGGGGED
VYCLALVPANTLPKT
RCFLEADPYIDIDQN
ASLEVEVPAKIHLPK
KKVPAPVPKKEKVPP
QVFKPFLPSIIALCM
YSVYGTEPYIRLGVL
MSPARMSPARMSPGR
PVPEEKEPASSPWAS
KKTFVGEPQRLGSET
KDMVKMFPLLVEKLR
LGAMSAAPSQPNSQI
TDRLVITPLTDRCYI
SLRQNETPQPAAQRS
LSKDLLKPIQDVNSL
TASPSSRPVASPGAI
KSQVRDYPKHNGQIS
GKDVLRLPPSSITTD
GLETYLGPLQVAYRE
REYQEYNPYEVLNLD
RVELLHNPAFCSLAT
SVVSITGPLIRILGD
PEQNQDMPPHLPTAS
RLTPIGDPTMVVEWL
TCRDEYAPPKAELDA
GDEDIKIPENPLEPL
GEIRSVRPLDREKVS
LSPPPTEPTDGEQAG

KKELKVGPYANTTRY
GKDELSKPSSDAESR
ATLVDQSPESLKRKS
KLKVLDKPGPPASVK
CTDSMGVPRALGETW
ANGDSDGPSYMSSLS
LGRVFIHPESPSTGH
SLKTAHSPNVFLQQG
DKIQQMNPPEFEMIE
ALLKDQQPGTFLLRF
AVGTSAAPVPSDNHX
EVKISHFPAADLGFS
CVDGCYCPNGLIFED
EGVLLTLPRQSGGSG
ESMENSCPQVTEVTA
SSSGAVDPSVQRSFT
XXMMAQFPTAMNGGP
EEVRVRSPTRSPSVK
CLEENERPLEDQLEN
SSSSGISPDNRDFYQ
ATSTSSEPLSSNQPA
TTCNCKKPDDDTRKG
MLTGESVPQMKEPIE
VPGGSVKPKIMSPEK
ILTTLQEPHDKHLLD
TNVEDVCPNIPSLEK
RLEYLEGPTVTSSYR
QSPTKAKPKVEDEAP
PPPSTQAPSVNGVCT
HGDACFLPEECPCTW
EVSIGSAPLAKQQSY
NILVELDPDGCPWLV
DDTERRSPTPERTRP
GSFGYKKPPPATGTA
XXXXXMAPVHGDDSL
APQQEELPLSSDMVE
KKLRSDEPLIDPKPV
AFTRTVSPGSVSPIH
QLSELADPLKDELNL
TPNPLERPIKMGWLK
FKELFQTPGHTEELV
EASKEYSPCVAPCGR
KSFATGRPKKTKKRS
HPYVTHIPSPTLPGS
DAWLQTDPEIQPSPF
SEIKISNPTEFQNHE
HNIENASPATVSRMG
SLESSSFPLPKQYLD
TGQLGVKPQTGNSIP
CSPLMADPLLAGDAF
FVYKDIKPGRKRKKH
TSANEVSPVSSSGVT
FQRSRSEPSPDAPES
ADSDGWTPLHCAASC
VEENNASPHFEPDLH
NQIGCLTPELSELKK
ETQAEDHPPRLYGCS
FKISFGTPAPGFSSM
GVCTDGEPSQPLLGQ
LSPDSHYPLEEEKTD

SPISQRLPPKVESLE
AAEFISKPQNLEILE
NKCLIKNPAERADLK
ELVAAVWPYRRLALL
RQTVAQMPPQLVELP
NPALTLWPMFLQGKD
QDIAKDFPRGEESLE
VTLKWAKPEYTGGFK
QHVFSAGPVSDEVLP
LYPPGRSPLHHAQQL
HLSSKWLPSSPSGVA
QLAPDPGPAGHTLFQ
GLCAALEPILSAKTK
PADVTGLPMPKIEWS
QKFRFNHPAEAAVLR
SAMEMNEPQAPAPSL
FHKVKALPLVSWSRR
AFKRANDPVNYLESK
VRATRASPDPRAYVP
KVIKMEEPLPAKVTE
QQVTAEIPVDLNTRE
GKVIGEFPGVVHCLD
MIKKQDQPTFDNSGN
SASDGYCPREHMLPC
LKHSPEDPEKYSCFA
LIKKHLLPLWNDGCI
SSSLGKKPSLTSESS
GILKRRYPNSLPALI
SKRQLQTPKEKAQAL
VYVDYPGPASLTQIY
AEEDLTDPVRCNSLE
NSAGRSAPRESRPVI
FQEFLKRPDHKQDFV
QAESQVEPSYSEQAD
LTAKQKLPSYILENN
RSTEGIVPQYDGSSS
LSLSLAQPLRSERSG
EMKEIERPFETYKEN
NATSAFGPNLRYIVK
VDQKIEHPLQPQPGL
HRRTSAIPRAFTRER
GDFKVTTPDTSTTQH
VISRTGVPQPTQAQS
RGKKAKNPKEEKKPK
YTLKKDVPDGVKELS
GNNDVKQPEGTGMID
VESEIKVPDVELKSA
KLGVQVRPENWCSQM
SWLKDDEPLYIGNRM
PAYDGGSPIIGYLVE
LAKILQNPITHSLQV
KLSSQALPSFGYIKI
PPARQLGPRSPRVGR
SKVWYNCPXXXXXXX
GKDFERKPLLEMNDP
GQAVTLLPFFTSLTG
KLAHGQEPWTTDAKI
PGGKEEAPEPPDGGD
QMHSTSDPSHRSSSP
LKNKGIQPLLDAVTM

SNIDIRIPTGQLTMI
CDEHFETPSGSYWDH
IDKNTKIPCKSPPPE
NAWERRAPLAPKHIK
TDWEKTKPVTGNLRP
CYHFGLSPDLPICKE
EALVIREPITVPERP
GDMGDTTPAEPPTPE
FVRPDLGPKMYNAYG
DQKYRQHPSNFQFKK
QMSFSQSPFLSIAKS
FSALRALPDMEVVGL
TSPEGVAPTSGHDRR
GSHVNQGPLHLGGIY
GRITDVIPSEAINEL
VAEYEVTPDDKRKAC
LVRDPLSPAVRQKET
GNICVYCPGGPDSDF
RLLERYIPKHQKCLT
PLTTALNPPVTATEE
FLTYHMWPLTFLSPS
GHGIMEEPELTLIST
KLDSEAWPGVLDSER
LRISLTHPTIPFNLM
ENSIEGTPSKCYCRL
NESKKTAPRQEAIPD
INPSTSLPLIGSPPV
QHRTFIKPKELEGLP
RVRVVGKPDPECEWY
LQALDLLPLLIQTVE
WAQQIHSPLTCEQLT
ITVITLGPPSKPKGP
AWDSGQGPRLPSSVA
STIGLNQPSTPTHAA
GEYDVTMPKVESEIK
FKTSIFSPMKKEVKT
DEREFSGPSTPTGTL
GDEGADEPRGAGRKA
STSKNTSPMRNSFAH
LRPHVFMPEVTPDMD
VDLSLGSPKLKGDIK
LLVGAKSPNLPEHIL
ILDGLYGPIAFKDFI
ARHRCQTPHLLAAXX
AAAARTEPYCAICTL
LFHILVTPLFLLSTV
AQILILEPPYFVKQL
PPPGQLDPEDQDSEE
CLVKDYFPEPVTVSW
TPELSAEPTPKALEN
TAALAVAPGPRFLVT
GRPAEPQPEPEEPAE
DLRTDAIPIRAAKAA
AGIAAIGPAKELEAE
EEITDNYPQAIVYPF
APPGLQPPAEDEARA
LVIPPQPPTTGPPRK
SRGTDGQPLLLPYKP
KVDALHCPPAAVVTV
TMKMRILPDQFSPLN

QVTTVKVPMMKRLGM
KKACMFEPLPIKLSK
RQQNTRDPDTMVPLT
EEALKVSPVSIPAEQ
IWWANKDPLPPLDPQ
ELKLSESPEPGRSPE
MMGLDLQPDLCSLLI
TRLFVKEPAAFLKRL
FDMHKRSPLLRAPRF
ALGRTYHPKCFVCSL
QESARAVPEKSTAPL
VVLCNLKPQKMRGVE
DASSSWGPQMSASVH
AGVSSGAPPGRNSFY
PSASERLPDEKVELF
TVSQFTLPKSVSDGI
TNSKATTPKPQKPTK
KESKVEEPRKRETVS
ALSSVGSPKAKEALN
EGQTQRQPGSPMYRR
KKPKTVKPKKVAKSP
AAVRGSEPISVTWMK
QFPFADTPDTYKSEA
LAQADKLPMTDKRVA
XXXXXMDPPRPALLA
PEVRSTEPVAPPEQL
EDEAPPRPPLPELYS
EKPVDPPPFMFKPVK
IQTDVVLPSCKKKAP
ALSLFTVPEDCPIGQ
SQPRDGWPMMLRIPE
EGGGRSLPRSSLEHG
TLSISVQPGEGNKAA
TKSNDTLPSSHREIP
ASAEGREPSPQCGGS
SGGEWKGPQVSSALN
YTIIQQMPRGNHFTI
PGQHLLAPEDVISCC
SLCVNEVPSFYVPMV
KGVTSILPVLREGVL
EQQAQFTPLADPPDI
IPLLLPTPEEKKPPP
PPTLSRTPEVQSRVP
TDQKDSKPPEKATED
EAMGLGTPSEAIEIT
IKTLSDVPAPRRLAE
TLLIELLPWALLINE
RIEEGERPDRAKGYD
WARVTKDPIHPYTKF
KLLVNFDPKILEVVR
ESKQATTPASRKSSK
LVKMKFLPVDDNFSL
NIETSNFPKLLLALF
HLDFLKQPLATQKDL
LNKLSMKPEPLFRSV
KDKIHTTPDTPEIRQ
DAAHGYSPRAIDMSN
KTKEKAQPLEDLAGL
ECQITGTPKIRVSWY
FGTLEVMPQNESETQ

ESEKSDFPPTPVAEV
FVNKADFPKVRAKEQ
SGQLVVIPSDHLPHF
ATGRPWLPLHTLSVS
ADLGVSGPKVDIDVP
WKQVHNYPMFNLLMD
PPCLQQNPGTMQGVY
GSAGPVHPLVDPLTA
AHLFSWIPLSASITP
LKWNFTTPRDEYIEQ
VKKTDSQPTSVMLLD
EGCANRLPNGHVNFE
RGGKKFLPVLKEILD
STCSSTFPEELSPPS
RSKLEDDPLYTSYSS
SVVSQRFPQNSIGAV
SLLALGSPMYSIITP
ASKGEMSPMVVETLK
HCLSFTIPRLPSSEE
IDKTCLSPTPTLEQH
TIFQERDPANIKWGD
NTDGLFFPVMSFSAG
KADIGCTPGSGKNYA
MTSTSLSPQGISTLE
KTVGVWSPSPPTCER
PRERSMWPLRKCPWK
VDYASKIPAQGGTNI
NRLCFLEPTSEAKKI
ETERCLQPLQMMALH
AVRMKIRPLVGQXXX
TLVVKGDPRDNRQPA
LLRRIEHPTAGNIEA
HVLLTLKPYYAKPYE
DEEVLTDPKIQALLL
LISDTKAPKRQEMES
SSVTFEYPSNAVEDV
TSSWLVTPKSLGNVN
KVYDRYIPPDLCVCN
CWYSQWTPAAPQCKA
SNGYKPAPLDLSDVK
LGVAEGSPQYKKWYF
IALTKLQPLLNKDSP
DTWVEHWPEAEECQD
GYLAATYPAVGQTSP
GGEFEMMPLGVNKSP
LSAEAACPMFNFSIR
YLCSAVLPLLTRCAP
MDVSLHLPSRSSPTV
TIEELKEPEKNGIST
AIDRFNAPGAQQFCF
GGFQVLLPLVDQYFT
ILLTDGEPTVGETNP
KMLSGFIPLKPTVKK
EELTYSVPYGREKDV
SLKFKEKPTDLETRS
DLGQGLMPVLEAWKA
SRLANRAPEPPPQQV
RTKKEGIPDARQATR
GCKEICCPVPDPKSV
FSHIDVSPGSAQVKA

IILLILCPEIIQDIS
KAHIQFKPTLSQQRK
MGNCGHKPESPTEVE
EKASKEAPVKGLLQT
LKNLLFNPSKPFSRG
RGGKGISPPSFSSYL
SRTQWEKPQVKESKR
NSALDWWPKIDAVYC
GVQREDVPAADLSDQ
YRDRSFSPGSYERET
KVNSGAFPAPIEKSI
AIMRYGMPPQDAFTT
KEVKSTAPEATVECA
SEGNIDSPVSRFMDR
TLGEKAKPALDDLGQ
GAAFSQKPQSNALIG
SNQAELLPSKSVSLI
LRNDKDKPLPPLLAR
EKSLSHSPGKXXXXX
GYYCLKVPLVVFKRE
FYQEVANPLLSSVTF
ALLESSLPDTDPVVS
EKCLAGQPKDTMRLD
GTSKAGNPIFYYVAR
YSRIKYDPDLFRMAL
TQKTVDGPSGKLWRD
EAERSGIPIVTSPYQ
TNLKIKHPTYCYEMN
YAAFNNRPPSTWLTA
ICLAQNSPSTFHYVL

Supporting Information S3: The benchmark dataset used $\mathbb{S}_{\xi}(\mathrm{R})$ to train and test the model for predicting the possibility of carbonylation at Arg site. It contains 136 positive samples and 847 negative samples. None of the sequences included has pairwise ≥ 30% sequence identity with any other in a same subset. See the main text for further explanation.

(1) List of the 136 peptide samples in the positive subset $\mathbb{S}_{\xi}^{+}(\mathrm{R})$

ASKLSKIRVVRKSIA
IIIQSYYRMHVQQKK
HCKAFKIRKHYLHLR
IHLELRNRTPAAVRE
LEELDRERCFLLNEI
YEMHKMTRDPLNLAL
RPPVSGLRRRKPRAT
KTQRSPVRIPFMQRP
KQFFKDWRDKDQSDG
KSEYKSYRLRAQLYK
KSRDEGPRLGDAKLK
SLRKLPPRSEISSEK
RKSTEFPRKIREQEP
GSVTGTKRLRCMPAP
ELQDMAARQQQIEN
RKLANAPRPLKKRSS
ASSRAHRRHRRQAVF
RALLTAPRSLRRSSC
TMLLPLLRRFPEQVR
SIDSLKQRLNVAVKE

EADEEKERILAQLRE
VADLEKQRDCSQDLL
EEEEEILRLHQQKEQ
IELLRKVREQVLKCP
LLAEVERRIRRGHAR
PDGENRARFLPGKDL
WERKQNLRPMPKKYH
RVLSGRWRLPLRALP
VSWGKLKRRVKGWAP
TLPKRVPRAGEARNL
EGGHFKLRVTISSNN
TLVEKEMRVTLAKLL
GDSRLKMRAELGEYI
REQEPTERPLKGIQS
ALAKHKERKMAVPMP
KDLGLHPRAVSTSFG
CLEVVLCRVEGMTEL
IKGLTYLREKHKIMH
MQKLNQKRAELKLVV
RIPQLWKRVSWDSST
GYKRKVMRMLKRQVS
ALVPPWTRVYRVQRD
KCSEETFRFELGGGV
GAGSGLGRLLALQFA
SQDKMEIRSCLPKLL
VDKSGVVRVRVEGDN
KERLQEVRDKLGLQY
YVGKAATRLSILSLL
RSVFTVMRRQLLEQA
QRGLTEKREELLSVP
RKQLIPIRHTKTREK
LLEAEKERNNKLSLQ
STEPLPGRKQVRDTL
RQLAIETRKTLAAQT
LSHPKIPRCQERLLR
RDRPGDPRRKRSSDG
FKDTDMKRLSMEIEK
LKRSLEMRERVLGPD
DRGHEELRKLESTLD
SGILRQLRQTVEATN
TAEDLRTRKSKLQEL
KAAAADGRGMLPKRA
PPSAASSRYSMRNRI
SRSYTRSRSLASSHS
HIAQKLFRDGHFNPE
ETSLNKAREHSLLRS
HVTKTRRRCKTVRVD
RLSSVFLRVRTNVGV
GQDGSGRRGPQKRGV
PRVFPPRRPHTPAER
APYVTLRRGLNAESS
TSQGKLLRTILFGVK
IYKPKRGRPKSKEMP
ENELTFLRSQIAAIV
IANVFQNRDYQCDTV
LMEFITSRKRGPLWN
VVRPKDRRVGTPSQG
RRSVRMKRPSSVKSL
PKTMPRVRKPKTTPT
HFHTVIARRAGMAAS

RIKNPKARVTEVMKA
LFTQELARRLKGSGV
WDATEDLRISRTDSF
ELIRLTQRLRFHKVK
LPLVSWSRRLEFQGE
FLQVETRRAGERLGW
LSYRSLRRRVRRLRR
VGLGLPNRLRFFRQS
GLAARLQRQFVVRAW
SLRKGIPRTKSVGED
ETRQRLLRTVKKEVK
AIVRQRERESHAAAL
ASPWKSARLMVHTVA
LGKRADQRKAKLGDS
IKSMAASRRAKLNES
QRMKALARALPLQPR
SASKASVRSISVEDK
EEELLNVRRELMSVF
AQLNIKERLIPPSFT
SDPIKACRPIKPPGP
HHVVSGLRENSEYFF
VVESSVLRAKEVTWY
LKRTKKPRPPSLKKK
KESLLQDRRQEEDQR
QEERLEQRLKREEVE
PEKENAVRDNKVYCK
EKLHLITRNLQEVLG
HGKQPSLRAAKEHAM
NCILDDKRPHVKKAT
NRRTLLVRPISKQDP
QADNPLGRSVLRKDI
GPPTGAGRGPSQLQA
KVLKNNIRFMNHMKH
SRLSRHRRAVHGPPE
LAPLNDTRVVHAAKA
YCDLPEPRKPLEKAV
CALSHHERLKCDEWS
VKVPMMKRLGMFNIQ
YQLEGNKRITCRNGQ
HKSEVAHRFKDLGEE
LPKLDELRDEGKASS
SLQKFGERAFKAWAV
EKTPVSDRVTKCCTE
CTLSEKERQIKKQTA
PLPKEPPRKDPPPKD
XXXXXXERIDGGITG
RGGEGNARTHGTPDL
VLGNDGHREVVKEVV
RGADYSLRAVRMKIR
QQARLHERGAAEMVL
LPKIETMREKVLTSS
CTKPESERMPCTEDY
YLSLILNRLCVLHEK
TESLVNRRPCFSALT
VGVNGFGRIGRLVTR
AITIFQERDPANIKW

(2) List of the 847 peptide samples in the negative subset $\mathbb{S}_{\xi}^{-}(\mathrm{R})$

VTPHTQCRLKLLKLE

VLSFSETRAHQVVQQ
ELSKANSRFATTFYQ
EEAAGSDRASDLDSV
GSNGVDHRSYRIIRG
HSGASQHRIARPSRQ
DSLLGKNRKALAKGL
IVDEVHERDVNTDFL
SDVEAHFRAAHENTK
ELFVKGVREVYDYYC
HASCYGIRPELVNEG
ASPATVSRMGMVYIS
RVQRLGERVVDSGRS
RSLSRFERSARFDIF
PNTYDIHRLEKILKA
EKPGGKERGSSASHP
VTEASPWRATNPFLN
KKKKKKGRMKKEDNI
GEPKDVIRKDVRAIL
PHQLLLQRLQEEKDR
LSLSHFCRSPFLCFG
TTEAPVKRASLLGDM
QEECGYLRRHHQEEV
VSNTLESRLDLIAQQ
KKKMTKARLERFKLR
VYTTKSPRERAILGL
PASPISQRLPPKVES
GAQMEAARPLVQENP
TKDNEHKRSLTKTPA
LHVTDAGRKHIAIAW
INYVLESRLIGTEKF
SSASLIWRSEAEASV
KFTTEEKRFVESRDV
EGEQYLFRIRAQNEK
PPIYTAAREQTPFRH
IRGNEAGRFRLDITL
EWDKDVARKLGSTTV
TDDSGTYRAVCTNYK
ESKGPMTRRLLLHEV
LDLKETLRLRILSED
DELLRHHRDEQKPAT
ALNMLTWRAEQEGGM
PSSGLKKRPISRLQT
CVIQSYWRMRQDRVR
DVWIDVQRRWVYLEG
WLLAHCGRPQTECRH
AACVMPSRLKALGTL
HKQTKKRRILIPSDI
SIQDWAKRMKALVEQ
QENCILLRLDNELGG
SQFNLSGRNPQKQAR
KSMSIVLRENHQKPE
LPEEIALRASDGPQL
EIEKLEFRVRELEQA
EKQVELYRNGQRLLE
AKKPAATRKSSKNPK
EGQEYSFRVRAVNKA
SISGCKMRLSYLSSR
RFRTLMMRRRFLSLK
TYDSYYSRSRSRSRS
EKETLLQRLTEVEQE

HWGQQQIRPIKSVIP
AVPPAKGRTVLEEKV
DLSTIQERMEELKGQ
SECGFCLRQFPRSLL
TPSEAHSRIFESVTL
RIQVFLARYSYNPFE
KREKEVARKLEFDGL
PLKTDIPRHPMPFAA
CYIFPGGRGDSALFA
RDMEKVERQAVPQAN
LDLDSELRKGIVVRA
SNQSVLHRWERKQNL
ECSKTSFRVANLEEG
MSTIEPHRQVAWKRA
YKDKRELRSGKKYKI
LEAQVLPRFQPRVLQ
SPQPPLSRAEAIKQV
ELEAHLCRMMKHSMD
EKKLEDARARCEGQE
TYSSAEERMQSEQIR
GLELSIDRFRLRKKT
SEIRITHRSHHFIPR
CSKDELERALHYLKN
HFIPTIGRLRKRAGK
KQLPQVPRPLQLFSA
CEPLSNKRNSNSVTN
HVHEASSRSHAIFTI
KNPASSKRRLKTSLG
GPKLEALRAEVSKLE
WICTKCVRCKSCGST
NCVSGQERGRLGVLA
KLNETMERQRTEIAR
NYSESLYRQAMEEAK
LAMLAKERLQEVRDK
EEKQEEERSKVDDLR
SEPGANMRHIRKPVI
HVEAVYSRRCVSFIL
QNQVGIGRPAELKEA
IHYLMLPRVREELID
YGLITHQRIHTGEKP
TGLETPDRKGKGTTE
KVVPPSMRRIRAQKG
RRELLELRRKGREEK
PLGLILNRFSADTNI
GNLSILSRQESSFFT
IQGKDVLRLPPSSIT
LNTSIDLRTNVLNDA
EKTINQQRIHARIGQ
KAKEQETRISEEITK
VLIPFKGRPPPTVTW
MALEETLRQYQAAKS
PGFKGEGREVDVNLP
RDLLLQTRLVNHVSS
DNKKKKQRTSSKKKA
WDDIITNRCFFLSKI
RKTRSARRRTTQIIN
YIRISPTRAYNRPTL
LEEELPKRVRSRLSL
QLKSFPARLRQYASY
STGLLPGRGPGTSAP

VKAGDSSRLECKIAG
ATEISTERDLGQCDR
CREVGLQRRSVQLFC
EFKQKLCRAEQAFQA
KVNGCLGRNCKLPIT
HQAAAATREASSESD
GAQMLAMRGEQLGVV
TEGMDDFRYACQSPE
IKKANMKRENKAYSF
FSCQRRTRFISLGEA
NSIEASLRCSSVVPR
ALTDEESRKNWEEFG
EEKPEEERSAEESKP
SQELDVKRREAIYND
EKKSKKIRDKTSKKK
DVFLDSGRKTRSARR
KIDHELHRLQALLKH
LCPEGVHRFQWIRNL
QDQKVNIRVAVLPCS
GATTSTIRSSCLVGC
APMTPAARPEDYEAA
KNLVQKERELEEQEK
QFGGIDQRKIFTFAE
QERDRKFRLEEQKVR
VVLPLDERAFEKTLT
ALAVEGKRIGKVLDH
EFLELKYRLLSLLVL
AKWFKDGRELSADSK
RVRPLSKRETKEGGR
IDVNVIARPSAPKEL
EAYITATRQYVAQAN
AQSEAAGRTEPTGPK
CYFHSGPRGTHDLWD
VNLVSVGRVLWQWTK
ALHTEITRNKDSTLE
LQVNDHVRKVTDQIS
VTLQSAYRGMKVRKR
IVTEEHLRRAIGNIE
CPGMMLWRYPEPRVL
EDVHIKGRIIDTVAQ
ELVPPSFRGGKQIDS
KLQTAIHRVTLAQTA
VHSPDIYRVVVEGER
DIANVMQRLQDEQEI
KFVGDATRLTDKLLE
GLTNPSLRSPEESTE
KGNEIVLRQDIMPTT
RSRSGKGRGLPKKGG
SGAGCSSRDPGPPEP
LEKIKAKRSDSGKYC
KFSSKYLRTEHGSEM
SKSIEKKRGRPPTFP
RKVFSPIRSEPRSPS
WYNVENWRTAVTSPD
HRLIAKSRELYEKTE
GLLFYSLRTLTTMAP
ESDSFCLRNIMEAPL
VKEVAAWRERYEDSQ
KLGKVNDRWQHLLDL
LGYWLEKREVNSTHW

ERDYLLERRDLAVDF
IIDEGLKRFIIEITF
AEAAHNQRAGAPGIQ
TSVPTTPRATPILQP
PLDQTLPRQGPGQSL
IKALADEREAVQKKT
LINGGSERKSCFWKL
VADLGNIRIRFIRKN
AKISDLHRESVDHLT
EDVTFNKRKMKELED
KKQQQQCRPSISISS
PKVLQDYRKLKNTAE
IKKLEPSRIVKQDEF
GKPKVLARTKGSMLV
AWIPVPARREAEKVP
SLDWLISRLERLEAS
RLKAQEHRAEMEEKG
DEAKTSARDAALAYH
PTTRSSARRSQAGVS
LYYYNFTRTVISSGG
DTQTIWARTGYLGFL
NELAYFKRENQELMI
ETQIDETRMGYKPVA
ALFPKGSRSVVPRDT
EVFTYSVRGLYENHK
PRARPRERDPGRRPH
NAALDNLRKVVPCYS
ESTSQIERPLSQEPA
YDGPKNVRSDISDQE
PFTPVPPRGEPVTVY
MKKFEESRAELEKVL
GTIDGEVRLTGELDR
SETKDPLRVQPHLKK
LMVHVDKRITLAAFK
FFAKLNCRLYRKANK
QKVAEQERTAQQLRA
SEILSNIRSCRLQCF
PLPTVNERDTENHTS
EQEQLYLRSGVVSSA
AVVDVSDRAVPPSFT
LEYCEVQRDPYSNYG
EDISKLNRAAKVVQE
QGETNKNRTKGGWQQ
EFITSRKRGPLWNHE
DMSMLEERIKRSAKR
AKQFRAVRTTEGEEE
VKERESERNQCNFKP
DKINKTYRSQLSSEE
EGVKYQFRAMAINAA
GPPASNLRKQKSLTN
DVLLGASRTLSLALD
EEVMHTPRTPSCSAD
PGLPVEVRGSNGAFY
PGSTVLYRIFTVNHK
AQGALPERDASRGGL
PVWAAKQRVLCALNH
LEKRQMDRAEHKGEL
ITESDAQRTMYPGSC
ETPSYAKRRRLAGPS
EDEVQFLRTDDEVVL

AVVLTSYRSTAERKL
RSEGEKVRVGDDLIL
LHNSLLQRKSKLQSL
GWWVRNTRILRKCIE
LLANAPERVVERRRR
RNGPAQTRVSSSSSH
SVQLTEKRMDKVGKY
EDTWKLGRNWGQSVE
SWIWVQLRTGLARDG
HCFHMCDRMVIYFFI
STPSLTSRKIHGLSH
QSVAKMFRAVEEGLT
LSVKDAVRVDSGNYI
KIYNIEDRTRVPNKF
DADLEALRRKLANAP
NFLASKHRQPPEYNP
CEPNCYSRVINIDGQ
PPEPVDKRAKAPKAR
NKPIPALRVVEEKKK
SKLLKNWRDLDARIT
IRNGVTKRGETLSWL
YTMESTDRNQTFSKE
CNGQNTLRNIVHLSK
PLPIDLYRYDDVSGK
LRGLLRSRGAWEQQI
EDELQLPRLPELFET
FKHIKDTRYMSSYFK
QRAAAMARTKVEIAN
QNSSVLDRLPQPAES
TSKSSDNRETPRNHS
KDTLMISRTPEVTCV
QLKNLKIRTNRIQRF
HQWIQETRTYLLDGS
QALLSKMRAIDLQIK
KKTWAKVRTAKFFIL
MSFKDAERGDDTSCE
VEEIMYLRQIYKQQL
TTAATVQRPGPGQTG
VGLWSMCRTNRGTVG
XXXXMSARTPLPTVN
PHPSPEARAPLASAS
DTPPSALRGSQSVSL
HCSLDALREFFSTIV
STSYMHQRSPGGPTK
LLIQKYYRAYSIGRE
ESESDNHRSSSDFFE
ARFDIFSRYESMKAA
TNYILEKRETMSKRW
KAPSESARGPPGPTD
RKKKSKDRPSKLEKK
KVELSQLRVAKVTGG
VTSQFTTRDDILRDW
GHDEDNTRISSAGCL
SGSFVSSRARREKKS
HLFYKIHRNRYPVCT
GLCIFFVRCRNDVAI
SLEKNLDRMKLDWVN
MTCLSIDRFIAVVHP
AETVPEERARGDPDS
NEHCPSKRDPSKLYK

LQQQEQLRALQGRQA
RLPTLPKRVPRAGEA
PDSMFGDRGEIIDPF
EGQEYYEREEGYDEG
EVNAAWQRLKGLALQ
TFQTLEPRASDAKNQ
FLALILQRTFLQASY
EHQMYEFRVKAVNEI
QVWETWPRAQELCPE
IVNLLKMRQGEEVFQ
QIHMIAQRRDAMAHR
SLSEEIQRRLEATGM
VYTTAEQRPNAYIPE
RRVRETQRHISTVKY
AFYEKMGRMLGSAFP
PGAKVTIRASLGPLQ
TKLLKALRVRKKTGG
ITLMDVTRNSVSLSW
LVAVLLSRTWHVRRQ
YLTVTLQRPTKELHG
AAMFQSERKNPAPQC
QDHFQVARQNHFPQR
SPRRRKSRSPSPRRR
SSEEKTQRMSLMRHH
KHKGTEKRESPSPAP
FLDVFLPRVTELALT
HFFFPKSRIVRSLMP
AVQKTTFRVTRLHEG
GYTEVQFRPYRTDDF
DPVEHGQRHTATKRE
HEYEPYSRLKSDIKD
TKFQKLHRDMEEATL
PSAMPQSRSTESAHA
VWEPLIERVEGKRQW
PNVDYQFRVCARGDG
SWIADCARHHCSSTP
VLESTTGRENSDQHY
ELSLNKQRKLNKSES
AWYMHGERAHYSHTM
SATLKNFRYHISLKT
YIKSELDRELQDRYV
LGLDEHNRVKVYRFX
LDTYNVKREAAEQWL
KAWKKHCRGEGKISK
VFKHGYERYNAMRAD
QLSEEQGRNLELQVL
IKVLEKKRAMFECEV
GTPVFADRLPEKMKT
DPSAPQPRAETSGKD
KSLLDWLRQQADYSL
KAAQQHWRHELASAQ
LNKHTALRGEMSGRL
TCMKFYARVCRSGSG
PKKVEMQRSLPGSLL
AKCQVTPRRNVLQKR
FVELGVTRVGHRMNI
IMEEAVTRKFVHEDS
EPIQFTKRIQNIVVS
LVIQKYWRRVLAQRK
LEAKQKARKKEERQS

LLEEALLRLLPAELP
YQTMLKGRCPSVSAP
LTKEKVSRLDRIVAE
KELADLARLHPTSCA
FNELEALRAESVATK
YLVLQQLRCNGVLEG
QMVTPPPRLVVGTYD
VPYFEQCRRDACRCG
EQEIVQKRTFTKWIN
KNQGLFHRVLREEIS
AACCGSCRNVSCLFT
SLAKFAGRKLKDCGE
CSSLSPQRLCSKHMP
DAAAAGDRRPAPDTW
LDINVVIRQVYHLME
DDQEIQKRLAEQQDL
RYIRSALRQEKALSA
LEKAAAQRKAKLDEN
SGEAPGIRKEMKDVT
REEAEKQRVASENLR
VLQKCSHRLQELEKN
MEFAVNGRPLEPSQA
AREHSLLRSDRPAYV
SNYQSTTRVKPFICT
AVASAASRPDSTHMV
LKYQSLLRMFLSYCI
NSQVTFPRIQVTSLS
DALTDPSRKTQKCLQ
EVLFLSSRSKVFERA
DTIAHLERTRENMEQ
AEQKSLLRSVASRGE
TSAEQDNRFSNKQKK
PANAGNMRYSHSTGK
MVAVLVSRTVGPTQR
NHHMEVLRELCEELP
LVNKDSTRRDSGAYT
AMVFMDVRMPKFKIY
LVSHVTLRLLKPECV
EKFGLEKRQGALELI
EGCEYEYRVYAENAA
YACGVEDRKSRARLF
QPDVTGIRLLSLGAG
EELHKEIRLIGNESK
SFLKLTTRAQLGAKS
LRVIEMEREGKGRIS
QIYGQKGRTDEILLN
IYNTKSSRERAALSL
VELLFNTRYAKAIGI
YGGSYGGRGRGRRTG
EDTQYQFRVYAVNKI
NSQPVAVRGGGGKQV
TPSPPPRRRSPSPRR
LEFDGGSRGKGEHFP
YLMGGTYRCTYTRKR
MAVGMGERPEVLHLT
EGGKGEKRKRETDDE
ITMWLSKRLPQFLQV
NIVWKYQRYHFIMAY
LTASPSSRPVASPGA
XXXXMAVRQALGRGL

QAMPYNCRNNLAFPA
LFGKMIARAGRAGNL
LIVCFIKRSRGGKYP
AHEKRKERLQMLQTN
ASLQPKGRRDPPLLP
LQRQHYLRQRHSAVI
ELKALAQRGIGYHHS
QDEWEIPRQSLRLVR
AALESLNRAVLAAMD
QFLEEEGRTLEDVAR
KDDIKSTRKLKESLI
YVRVKTERKNFLAVQ
VNSTHWSRVNKSLLN
VDTKYQIRIQIQEKL
AGLSTDNRNVCLWDT
PRRASPLRTSRSRPH
MPSKFSCRQLREAGQ
GENQLLERRRLYHCA
TTIDDGSRCFFTKSK
AAKVFGLRSRKLKLF
ERLKEPTRQALQQRL
VIFYDFLRGLEASWI
DEEASYLRFRNSIWK
PKNDGGSRIKGYIVE
LQVITTLRTAAKEME
SGEGDLSRHLGLLGL
NDQLEEQRQEQALQR
QEGSLSARWPVAGQI
RQILRLLRLRCTEND
DPVDSTLRDFCGRCI
PVYKTSARRGTLSTT
KTIGSPKRIQSPLNN
TNSVTAVRIQPLEDI
LIPVIGPRKNIKKQQ
ILVNTNLRALINKHT
WGTATVGRPRGPPKA
WLEAQEERLKTLQKP
GETPEACRQARSYLE
LRQCLAPRLPHSVTC
ADEIYELRVTGRTQD
NAEKYYGRKSPVGRD
LELESQKRLYEKNLT
KSEAKLSRKQVDSEH
SSLSSAGRPGPSEGG
KVFAKVLREEDSKDA
FIAKAFKRIKDSEFE
QHHRLESRYSSSSGG
LAVGKFTRTSGETTH
VKMCVGSRRMVDVMD
QRPSGEDRWQEKDQD
GSKKVTIRSPVQIRN
TSENTLKRVSSLAGF
QPGTFLLRFSESSRE
TDFWKTLRYLSLLYP
AEPGEGTRPATVGDS
PLSPPQHRYSEGERT
GCFLFISRSKPLKTL
VFLHQKPRLPKLFKQ
HSYSVLERFVEECFQ
TNDLTAKRLLHVKGR

KSLKAKFRKSDTNEW
LNELEILRNSAVSQE
EWKFKLFRVRSFEKT
KASAAMVRLRLYDIL
EYNAVKEREFHNQYR
MVTMGFARDEINDAL
INIDQFVRKYRAALG
DISALTLRISELESQ
SCFKDGVRQIPIESI
PWAKYVVREGDNVNY
LSFLDAYRNYAQHKR
SESEDEQRPRTRSAK
IGSDSQGRATAANNK
EEPGSSSRESVSKAG
GPIFTTARQLVHALA
GQSPFQQRKKKIKRE
DALEEQQRCISELKA
YKTLHDTRTHFLKTK
HSSEHAGRNGRNAAD
AAIGATPRAKGKAKH
SKRKVLGRRDSDDDH
INKEAQKRWTRREQA
SFMSLQLRELVIKSL
EHKTMASRAKVMADW
VKQKELSRIEEALQK
QYSFNSQRFAELLST
SLHYALARKGGAGGT
AHTKPLNRRSVLEKN
SLDAANARLMSALTQ
SGLDIFERINTSAFE
AVDSPAGRQQLLQRG
LVFHSITRSHAENLE
AEYTVVARNKYGEDS
NAQILSQRIEKAKCL
EDKGEPKRRGYLQVN
YGQIESVRFRSLIPA
ENQQCLLREETWLAS
HQVESFIRKKLESLL
MKFKQKPREEQLEAD
ASAADTSRLSLWGIQ
LPKVTLSRDGVPLKA
ETLGVLARAVGEPMR
EPRRLSSRRSVLTSP
SVVWLKDRGEVIRES
ILTLAWDRVDIAKNH
VYNENSNRQKLEHVK
FKIAGDMRATCPAFN
PSHGLADRVINCREV
AQQEELERRKRLEQQ
MEDKIYDRQVTKQSL
EEQPTSQRDRLSQVL
TNYVIELRDKTSIRW
QKKSAFTRTVSPGSV
EVLNEVNRRSLKKLA
QTAGVIDRWELLQAQ
RSKVDDLRGTPMSVG
REGITTKREQVQITQ
EASTSALRESCQAEH
PANGFLVRYLRRKLV
NNTDEMARLIRSVMQ

ASERKLNRRGSMSYL
YSASTPVRKPRPGQQ
EMDISADRVKVEGEL
WDLSKKLRIPAISRL
SLALKNIRCRRGIHK
ALQAALARVVPSYMQ
GMYSEPLRQFRDSSV
MDGAAWDRRNGKLME
GSYLQFLRILFRMLI
LKHFIGQREEFETAR
LVSKDLIRKAGVGSV
GFKSHLIRLIGNLCY
AHEDISQRVAAENQD
EIEEDKARRILELSG
SMLQHLLRRLVFDVP
ISPPSPDRPPHSQTS
RSRGKEQRKLARQRS
EEAEAAARALARFAQ
TLTIQKARVTEKAVT
EFTQDLFRFLQLLCE
GAHKATLRIGQDGIS
ANLKMELRDALCAII
ENSLETLRFSISNLS
MFKWLVARINRALDA
HKFLTIPRLEELYNT
RYNERKGRSELIVVE
SVDVKSVRMGSIQRK
KGGSKLLRMKLSDFN
TGTPSDPRRRLRSYE
PEVFKYNRFLNPDGS
ESVKASERPLPPGKI
AYPICEWRYDACASP
PETIDTARLHYRNSW
RSALFALRYNILTLM
EPLKELFRQQEAVRG
GDEEQKLRFAFSIYD
YLMLDNKRKEVVHKI
RLDPVSGRLSTISSL
KPQDYIPRAPTFLMN
NSHHECIRKLLQSKC
TSSLSPQRSKLRIMS
QGLKIHQRFHFASAL
SSKPIKIRGPSHCAG
HANNVTIRESMQNDV
PGHFVGPRGPHPSQF
GLDPTQFRVHHYHKD
PSTNTESRKDVITIS
IDAIGTKRYDSNSGG
AESNSNMRCTCRIIE
VEVAATERTLLGFFL
YDYVHSVRGKVAPTT
SRALSHDRQLISQDA
GLPNNAERVLLTTQG
FDFPDLNRFLTRTLQ
GGKPVQNRELQGYES
LSLQVVNREIREENK
KAFVTLERFDQLYGS
EAVKYSERSLTKCIG
FGKKAANRSWQNVYC
TLRMRSHRVPCGQNT

QVSETLKRFAGKVTT
EAVNVCGRATAVVEV
EKALRSMRKAQVSPQ
EKVLSPLRSPPLIGS
LDLVSHPRATQTHVE
LISQINKRYKTKDDI
SSEKVAERKETEFFS
TAKPINPRPSLPPNS
EAKAELERKLSEKDE
ITKVGEERKGPLPKS
LVEETEERLVERISF
QIYSTGLRKGNLHNV
KHFPNIDREKAMSRP
SMSKGGKRKDSVWGS
WADPTGTRKLTWTYA
AQIADGFRIRVDLAS
LRDWNLIRDAATLIL
AEAADNQREEAADNQ
VYQIKVHRKYTGEDF
ARLETLVRKAGEQQE
DASPGDKRLAAYLML
LSSYATARLMMNNEY
KLPVDFERSFQEEKP
GPLPYPERQKRARSM
DSAKELQRSVEFDRE
TGTLVSKREVELEKN
GLFNKNPRHSSSENN
FNVPDEERCSFATVN
EREREREREAERAAK
QQLNKDTRSLGEEPV
FSPLISARFGMGSMP
KTKTDYTRIKSLSIN
SSPSSGQRSASPSVP
QHTTMATRSPALPPE
VQQVTFSRGTDGQPL
KKKGGLFRRGSLLGS
GNFTDAERRKCEEEI
FLNPDPLRADGISDL
WFETQEKRLKQQHRI
QGYIIEKRRHDKPDF
SLWSNTVRCYLIYTD
FDFQAMGRIFVGLCQ
TPGGTISRASRDLLK
DTDMDSLREARREDM
KKLRLKFRDVKCIPD
EPSFEATRSRNRYSV
SSGKSVARTSLQAED
VMLADSPREESNKRQ
LSCLTTSRSMTDKLA
KNYVIDKRESTRKAY
VWGGIWPRHRFSLQV
GRREAGSRAHPLLRL
YIQLEVPREATISYI
DSVSEATRDQDGVLR
NQLAEKVRLRLRYEE
ILKPMMLRRLKDDVE
VSPFPKKRGRPKRQM
ATDQEKSRKLHELTV
MVVLSLPRIQSQRYQ
XXXXXXMRGAMELEP

FREEEQLRQEREEQQ
QNLPVQDRNLMLPDG
DEFTTLARDLYAFYP
DMAAHEERVNEVNQF
TAEDIQERRQQVLDR
KMAEESERRAKEADQ
ALHQKTLRVDVCTTD
LAAERHARLNDSYRL
RLDDEIGRKVYMRDR
IKIQAMWRRYRAKKY
ESLHQNSRATLLYGK
QSKALTERLKLNSKR
PSLEGDLRGPDVKLE
TFYVTKSRDALTETA
AVSSADPRAPGESPC
PISDGGSRVIGYHLE
PHQIGGIRFLYDNLV
GNMELEKRRQALMEQ
EEQQESARAVPEKST
PDLEKVVRYLAGCGL
EMEPLVPRVKEVKSA
YNINTYARFCKCCRE
EKIDLEVRSLETCMY
LAKMFLNRINYWHLE
TILDMVERECGTIIE
KLAYYEIRYQFYKRD
VPTVTWLRKGQVLST
DMNKVKLRAIERSPG
LLDSPYARVHIAEDQ
GKMPLEERIQKIKEI
KLKPVELRELLNPVV
LQVVACTRGGMSRLF
YLQHLCYRNDKVKTD
DEVEVYSRANEQEPC
EDFSQLLRNFGQWLQ
LLFRGANRDVRNYNS
TKSEKLVRLHQEYQR
GPLASHMRWFIWLMA
QSVQETVRVRKVDVS
SLPCLSLRKLPPRSE
KEVEVAKRNLAQQKI
LSPGNSARLTPLRYD
TVGDSSARPARRVLL
QEEITEYRRQLQART
MPWKTCWRYLTSGGF
IVVEEKVRVPEEPRV
PSFSRQLRDVQETVG
KKFGGPGRMKQSCVL
TSLQKLFREVRIMKI
PPVETIQRTTARDPI
SQLLTSVRRMVLTLT
CKLAALVRHRVSLFG
TLFSNVQRVQGVSER
EVQYEIFRSLMYWMT
IELPDLGRFYKIRVW
AAEELLQRCISSALP
ELSHDNIRVKWFKND
VPDALNDRHPDVRKC
NNAENLFRAFLGELK
VIAMLGFRLDAVKSN

KNMLIHGRAMLLVGR
AQAGAKSRPKKREGV
DFEYKLDREFLKGCK
LDALVMDRVAFVKLL
VSIEAPKREPQPIKE
PIGYQCSRVYWSTTD
SANKENFRILVESVK
HQLTEEDRERLKEEL
EAAQLSERTAQEQRT
ECIQMKARLTQELQQ
AGPGLLPRKPPGLAA
NFPVHLSRIRRLRAE
KVDQFVTRFLLRETV
HYPFENKREFEDAFP
NNLHSGIRKLEDYLT
RNLWQSSRPLICLLD
IQSAPNVRFQWFKAG
ISSPPACRSPSPVAN
GYPGGKARIIHKESD
DQRLHTTRSVSMQDE
APPVTSARFPSWVTF
DKCNDIIRNKDDTAA
IEVPISGRPKPTITW
TEINAKLRNIIVLDS
VRQEQHKRLEDLRRR
ITDASALRLVDKAQV
SILLDFTRGSRGRKN
FHHCEQCRQYMDFTS
EYEMPLYRKYWPNHI
IRPRPPSRSRAAQSP
RAARTLYRIELLRKV
VGGSGLPRGRRPPRW
MHHDPFLRSMAYWIL
LCTVATLRETYGEMA
CAKQEPERNECFLQH
DDNPNLPRLVRPEVD
KYLYEIARRHPYFYA
ELLFFAKRYKAAFTE
KASSAKQRLKCASLQ
FKAWAVARLSQRFPK
LLECADDRADLAKYI
MFLYEYARRHPDYSV
YSVVLLLRLAKTYET
FQNALLVRYTKKVPQ
PTLVEVSRNLGKVGS
CKHPEAKRMPCAEDY
CTESLVNRRPCFSAL
PIPQCPPRLDVQTIQ
WNPIEGERYLSFLRF
QFLEQAQRELAELLD
LNFTMNDRHTGPAWN
CDCELQHRVEAIVAF
FCQEMKFRNKMVEYL
QEESQAARIIRNTTA
LVIEEQLRRAAYLNM
DDIKCLTRDLDQASM
DMKADVTRLPATIAR
YLHLLTLRGSVLRVE
MKCSSMQRFGERAFK
GKNIYVERQRPALGE

SKPEGTPRGPQVYTM
KLTGMAFRVPTPNVS
SLGKVGTRCCTKPES
GPLSLASRNRSQDSG
LKNVNNMRIFGEAAE
CQKGFVLRGSGVIHC
VVVKLLIRRPECFGP
CAKQEPERNECFLQH
TCKKFDLRVTIKPAP
KATLKNLRKQYRNVK
LVEVMMARRDDLSFC
TENAIGSRKFLSLGL
YHKDLQTRATFMEVL
ANIHKEQRKFCLAAE
CSGSLVERRPCFSAL
YDDKAIERLLDRNQD
EHMPNDSRLKCLLDP
VLVFEQVRLLMKSYS
TEILKWLREILICRN
SFEVDPTRLEPSESL
NMGQWKIRAFYEHAP
FSKKCSGRWKTMSAK
EIMEACMRDIPTCKW
VNGILVSRGVKDMEL
TLGSTVGRLQEQLGP
GLAESGARYTEMPHV
PLPPLLARVGGNIEV
TYPGLSKRRLKPEAI
STLQTIYRLSKGRSL
YPVGTVLRYICRPGY
GGITGNMRQEAIDRF
EKETDWLRNEMNKDL
ANQDFTNRINKLKNS
KYLPGLLRSGFYDLL
GVVWVPFRGADYSLR
RIPPVAVRLQMSERN
LFLHGYQRFWIETEA
LAFDVGLREHATGYL
VCALGNSRVASALCS
ENAQTGGRKRGMSRR
VSLHLPSRSSPTVFR
LHKTIVKRRMSHVSG
YELLLKVRPEQLVKH
PMKYSCQRRARLITQ
VLVNSLHRIITNSAL
DTTVKKTRRRGQKAE
EKADMEDREKEDKIK
SCSRSVSREINLKDY
WLISKLDRLESSSGI
PAVNGTLRTSSYEEH
WVQAVVSRFDEQLPI
VPLVVFKREKEIARK
DLSWSMLRGCKEICC
KGDPKKPRGKMSSYT
NPDGSPARRVPVVTQ
IPITSSTRNIRLYPD
MLQQLLRRLVFDVPQ

IQQQLEQRISASGAE
TSFSNYYRTWNVVVH
EAGGAGTRARSLWRV
GQPKDTMRLDETMLV
DDIAILARYMLMLSF
FQNAILVRYTQKAPQ
AHTVVTSRVVNRADT
GDLYGAERIAELLGL
QTLWLWARPRAGGAW

Supporting Information S4: The benchmark dataset $\mathbb{S}_{\xi}(\mathrm{T})$ used to train and test the model for predicting the possibility of carbonylation at Thr site. It contains 121 positive samples and 732 negative samples. None of the sequences included has ≥ 30% pairwise sequence identity with any other in a same subset. See the main text for further explanation.

(1) List of the 121 peptide samples in the positive subset $\mathbb{S}_{\xi}^{+}(\mathrm{T})$

HLELRNRTPAAVREL
PRKRPPVTQAAGALP
LLAKQHKTQRSPVRI
NEPEAEKTHLFAKQE
RKSLVMHTPPVLKKI
GSKRQLQTPKEKAQA
KDGSVTGTKRLRCMP
DTEEADRTSSKKTKT
MPRRLTQTGKRKHGA
GTARRTGTPSDPRRR
NSKHLNGTITAKYTY
GKEMLEQTLQKVTEL
NELLKQKTIELTRAC
LDTMSKKTTALDQLS
ASKEQGPTPVEKKKK
DLIQHRRTQDHKIAK
IEEELKTTKRKMNLK
VQKANPGTLAAEIRA
WIWVQLRTGLARDGR
VSAAQGTTGTAPGAR
FQASLPLTRIDEEEA
LNDKHLKTLSKTVFS
RPLKSQITVTRHDKR
DSKNSKKTNTEFLHT
ERVEQFLTIARRRGR
PDDRTARTLTLIAKV
RDLRFVRTAEILAGK
KDNIPPLTMKRIRER
KGITFIFTDSEIKDE
MHFRSLRTKLLLMSR
RQPVTKNTFRQYRVL
SEVLVQQTLQRIRQS
FVQRRGHTVLKIHLP
KAKKPAATRKSSKNP
MAVLEALTGVLRSCG
HSGKLYKTKSNKELH
VQSIKLITEGASKRI
YSAEWKSTVSRLGRW
GAFKAVMTSIKQLSS
RDKLKFPTRPKVRVP
VKRGDIFTLEEELPK

QLIPIRHTKTREKVD
NEIVNIRTSLLNLVD
KQELEVLTANIQDLK
PKDEDERTVYVELLP
EKIRLAKTQQASKHI
EDLGRKRTTMLKIAT
EVAEKQATDVKPKAA
EKFKALYTLPRSVDD
ARIKAKLTQIRRYGE
SKGELLSTLLTKEKA
LTKLKAKTDNVVQAK
QWIRATATALERCSE
PLGAEKITGELEEMR
ISNVDVETQSGKTVI
LPDMSVKTPKISMPD
RSIRKSSTSSDNFKA
TPESKKATSCFPRPM
SVFLRVRTNVGVRVL
SVSVEKSTSSNRKNQ
TSPLQSPTKAKPKVE
SQGKLLRTILFGVKR
STAVGLVTKDPGKKP
LGKKLGSTVGLVAKD
NYDKILATKKNLDHV
RAALDKATVLLSMSK
LLRAGYLTLYGIEAL
ETKETTTTNKQTSTD
TPKKPAPTTPKEPAP
RTARAITTRSGQTLS
KNGKVYFTSDAGIAG
IALTTYETLRLCLDE
SSVGWDATEDLRISR
TEELIRLTQRLRFHK
NHFLQVETRRAGERL
QTSLIVATLKKMLPI
DVKSTAFTFRSSKEK
NKEKEMVTSLFCKLG
LRIHAHFTGLRYLLY
TSSPPRKTRRLSPSA
RLLRIAATPSCHLLH
PPKRAPSTTLTLRSK
LRKGIPRTKSVGEDE
EPARWDTTLPTPSPK
WKKLLEATELKGIKL
VEKPPKFTEKGNLEV
LRLLNKHTALRGEMS
ATRVRNITKKSSHLP
SYKLKSATGSVVSTR
ITKKMDKTIPEGRIR
VARQTLETIRSVGYY
DLKTIYQTGCQTSTV
ITKSSTSTIKDKDEL
VRVTGIPTPVVKFYR
IMKETRKTVVPKVIV
KKLSDISTVVGKEVQ
SSEPKECTYTIPKLL
VDVKFKDTVILKAGE
KILSNNNTSENTLKR
CLQTLLDTKFVHFID

KPLKKKPTPVLLPQS
GIISSPLTGKRQDSG
VLCIIQDTTNSKTVN
QLSKKLKTVLDQARQ
LEEIKERTATGLTHI
KKTILMVTNRRVLCI
RKIKVSNTLESRLDL
KGFTVKRTLVIHQRT
LLAPLNDTRVVHAAK
NETRESETKLKELPG
DGTWSPRTPSCGDIC
RDSARQSTLDKELXX
AVSEHEATKCQSFRD
KEGYYGYTGAFRCLV
SSLLEACTFRRPXXX
LEGNKRITCRNGQWS
QKLEPLGTELHKNAK
CLLPKIETMREKVLT
KVGTRCCTKPESERM
TPVSEKVTKCCTESL
EKQIKKQTALVELLK

(2) List of the 732 peptide samples in the negative subset $\mathbb{S}_{\xi}^{-}(\mathrm{T})$

HGSIFSGTAAPRCEI
SRNKSGRTALMLACE
LPAIFEYTVVGEPAP
SIAFQMPTIAKDGNV
YDINQIITTAVMTHT
GAAGLEGTAATAAAG
AREPPGSTAGLPQEP
KMMHIAATLIQRRFR
CPPGLYQTVPPEEHY
QNIPPSFTKKLTKMD
SASAPSSTPTGTTVV
VSPRGMVTRSSPGAG
DEDDNMSTVMRLRTK
ARNGISPTNPTKLQI
RELQARLTLVGKEGP
NLKFQPPTLGPEPAA
SHRVGAATAGRLPAS
PAAASLSTATDGLAA
PKESMLKTTLTAFGF
YPEPRVLTLVRITPV
DSLPSPSTIVSGDIP
RVNKVPVTMTRYRST
HLRKAAITIQSSYRR
RLLMASPTRKPEPQV
QYHKMYRTVKAITGR
KSKKRNSTQLKSRVK
KAKEDDKTLSKEPSK
GGGRNDITGRFTRHL
ENHPQWDTAIEGDED
DRWNSAFTRKDEIIT
SEPSDAITCRDDVEA
PRCAEHGTCRDGKCE
NGDGGCGTGGRNCSA
LCKAFVPTCIEQIHV
DFEVLMKTAHGHLVP
IVYHRKVTDDISKIK

RSSRKICTIGIAPWG
VQGDSQGTPTSSQGS
YIVQKRETSRLAWTN
DRKILDDTEDTVVSQ
CRDELVRTTAQYDQA
QEKSPVNTKSLFKRL
IEELLSQTTNPDRFS
ATQPAAPTPTATITS
SKKDDFFTSFKISCQ
EWRRANHTPESCPET
PSAFYQQTLPNSHLT
MREEAERTRDELERR
GHMYMILTPSDIQIQ
SPQREVLTVPEANSE
KLESLVLTHLSRCDS
FSCGADGTMKMRILP
KPESIKVTTGDTCTL
VVKYRAGTSVKLRAG
LKDLLLNTMSQEEKA
SYSSLLETIEVKGAG
PHGGAMDTHFANMRS
KFTSITDTPEQVLAK
AQEERLKTLQKPESV
NPYSLLDTSEPEPPV
ICESLEKTKQKISHE
ECGLGHRTVPLLLAE
SFIDGLATFQISGAR
SSAMESLTKHLFAIL
RKLDLSDTKSIRAFA
PEAKCACTDSMGVPR
PGPPVNVTVKEISKD
VSPENLHTEEILVCG
VNIQLKGTNEYVPRF
PIAAVLATFVTHAYA
LESHMILTETLFRKI
RLTYTERTKSTITLD
LSRKLRETDSQLLKT
GDKRRVFTFPCLSAF
VLDEDEETKEPLVQV
SSQEKVATLTSQLSA
ESVSSLSTNDFSIPW
CFIKLCITLNEGKSI
SICSARVTLREPPSF
KELNALETSSSAMDM
SSTCEPSTVAAVLSR
ILKAALITEENQQLS
NKYHWEHTGLTLREV
MRTQEDLTQGLLLID
DKEGRELTLEKPELK
HQYKLKETIQKLSNV
QAEAVLKTLQELKKL
LDCVLDLTMLKIHNS
AKGDRAATLEEGNPT
LKGTQEITGDDRFEL
LVVFNHLTPPPPDEH
VSPLLSRTEFCTAPL
FGQRLDETVAYEQKF
KRLQEEKTQEKIQEE
PTGPELDTSYKGYMK
VTPEVKKTSFHVTNL

NSSNFMNTTNFQSLR
SASKHSPTEDEESAK
VEGAKKVTVTELLAG
ENMNRSETEACFFIC
EVCQKSLTGYLEKKR
GLSPGSLTLSAPSTH
GEQLGVVTNWPPSLE
LIGPCCATANLEAKW
AELERLRTQLLFSHE
GQTSMPGTLVLCLPQ
TLQRTGQTILPSLNE
VNSSVKRTQIKVTHL
MASFLYSTALPNHAI
TVAKELQTLHNLRKL
IARILKDTAIKSADN
GDGDLMMTSFERMLS
RIFADGKTWSYTYLE
DLRKVYGTVLSRHHH
ALGRDILTKTKQARE
AMMSVDETLMCSFQI
XMSLADLTKTNIDEH
AAGGPKLTKRGLAAP
SPAARISTSPIRSVR
FKGRPPPTVTWRKDE
AALECYNTFIGERTV
MAEILSGTESVPLTQ
LLAPVALTCGSDGSL
YEGWRIDTYLDIPLV
QQMCCVSTSIVSFVR
YEGGAVCTHARSLWR
KFHGVLKTLCLEVVL
RMVYCSLTDFQKAVY
SQRVNGLTSSKNSQP
CSLRPNQTEEGTTPP
HESQISFTIEGPLTS
AEAKPKSTCELYSSQ
SESPVYPTKFDSEKE
VEKLLDCTVIVDSVF
TVLKSSATFQSTVAG
KQQKEEKTKQEAYAQ
GCVCSEGTILHRRHS
LERQNLETFKDRMTE
IFESVHGTAPDIAGK
LGPADQKTGTPTPTS
KLCYDAFTENMAGEN
KKGASFQTVASLHKE
KDAYKVNTNLDYKKQ
VGKPGIPTGPIKFDE
QKSDDKVTLEERLDK
RDFVGQRTVTFSSLV
LEISEDCTYADVKAL
KNCREAFTADGDQVF
EPLPAKVTERHMQIT
ALMRSEETADLLAEK
IDHKEKSTEINHEIP
STERLKKTNEILKGI
CEESLSQTPPRVTGT
GPSSERATPAFHPVC
GPKQASFTDVRDPSL
KIQQLVNTLKRPKRP

ICELSFETEHILLQH
KKALLPPTVSLSATS
LHNFSIYTLLGKQVT
KDLVNRYTQNGSLDF
KGEDPLATDTRVSVE
LKANQVATGIRYNER
LQSHFIPTIGRLRKR
AFMDISATDLVLRKV
AFLRHKMTLISPIIL
YLRNLPITAHPEVFG
LWEGKPRTYITEERA
IRILCCLTFLVKVKS
GYGSNKKTKHMLPSG
EPALVPGTPKAEDRM
GYHVINNTFQSLLGC
XXXXMWPTRRLVTIK
RFRHLKKTSKEAVGV
KLAQLIATCPPSKSS
LKDSDEITEDDNIRM
GGEVIEDTYGAGGGE
PKAAKEKTVKKETKV
PPEVLDVTKSSVSLS
HPSVVMMTAGRCHTL
AVPAAISTSEAAPYA
VDRHREKTLRLLWKI
SLKSLILTALQRETE
RVDALERTLEQKNKE
AGGSLKVTILQSSDS
TLTAYIVTSLLGYRK
GKPVDGLTTLRNGTL
TKQSFSLTMSIEMPY
LVSKELSTWKERPAR
GEAGGGSTAAEEASE
IACDNIGTPLAVFSG
RPCDEDKTDSETGKL
RTSAPSPTALKLATV
RGQPRTRTRASVRGF
VENMILLTIQYLVRL
VMADDEFTQDLFRFL
NVSQDLDTIRSNINN
SRSKADYTSHLRSLV
KSRHSDGTEKNKLPS
SVPKVEGTLKGPEVD
KEEVPEPTPEPPKKQ
ADGLQGETQLLVSRK
NKVQLMVTDSELSNQ
ILYGPPGTGKTLLAK
CIEVVFVTKFLYSIL
EQSIWNVTVLPNSKW
GLNMCAPTDQDLITL
SLIKQIATKVHPGGT
HGREAKVTETARVPA
TDGSEIKTDEHYTVE
SFIWELITPTKDGQA
AAMRAFKTVTTKCSK
SDKFYKQTVNLQLQP
EIVFCAVTSNEQVKG
PPHLKPATEKLIVVN
EVKGAGMTEHYVTQL
CLKEIDSTLYKNLFV

SPNPDVWTSEQNPPY
EWPFLIITDLFLKSP
YEAQKRITQVFELEI
KAVVNEDTQGNVSQL
GFTFGTATLASGGTG
PSEAIEITAPEGSFA
AESGIRYTQMPHVME
DALCLVLTLMNGGDL
KSLQAVITNLTQGEE
LQPEPINTPTHTKQQ
GTLVSHVTLRLLKPE
VNVRRDSTGMPLWLV
MFDERIFTGNKFTKD
KVLKWNFTTPRDEYI
DITENAATVSWTLPK
SSIQEQITKANEEFQ
GSMLVSWTPPLDNGG
FLPLFKATINPQDHR
PRAEKEDTAELGVHL
CLSVLSSTSLRLHSN
QSLLQLSTCDVESKR
HSADVFHTFRQLDRL
HQQALPGTHIPEEAQ
SAPYLLATSCSDEKV
GLVVKAGTTVRFPAI
ALYSRIGTAEVEKPA
IALSSSETTKHATNT
NRLVDYITKTSCHLA
AEAQPEATAKNLLHS
FFLYSKLTVDKSRWQ
CVSSIFWTQEVSQAL
ALQPSVLTMCNGGAG
YKDSRGLTPLYHSAL
EPKECTYTIPKLLEG
EALIKKLTKQDNLAL
AIQIASATMPALSLN
QKEVEQQTGLSVFLP
LEEKAAMTDAMVPRS
NVIRSIQTDKREKYY
AGGHPLDTPHLPQEL
KVQIHHNTWLHFPGH
KAEPLAFTFSHDYKG
KLYIEAPTFDLQGSY
DCNKAAVTIQSKYRA
TILEPLFTESESKIF
RHLKESQTESTNAIL
GDGFYLPTAGAPGSL
LSLSVELTEAKLHHG
PSQFPSETAFLEEFG
RVASNPYTWFTMEAL
PSPFDCSTDQEEKIE
TSESSIHTITPSVVN
AELENIATLCFKALE
EQVAWALTKFPCLQL
KNYSLKYTKYTKKDT
QHPAASPTHPSAIRG
QNAQVCQTNPEPPAT
IPVNEKDTLTYFIYS
XXXMAAATVGRDTLP
IILGNPATELSVATH

KDNSRDETFHFQCDC
YLDGNEITAIQKHGI
LFGDVGDTGERLLYN
KKAEAVATVVAAVDQ
NLELRNLTVELEQKI
VEDNKRFTRIPLVQL
EALHELRTAEEFKTE
QDLRVLPTIDLSTMP
LADDLKKTVTIRAGA
AVNNYHKTNPTGTQE
INKFAEYTHEKWAFD
TIRGDLETSNLQDMQ
GEGECEQTPEELEPV
LREAGSVTALVQCVL
VARREQNTKRDIPDK
ELHICFETTKSNEAM
LGHDEDNTRISSAGC
VVRDAVITSNGTLPD
QMQLDDSTQLNSDLK
VKSVNSGTWIASNSE
RGMINVSTSSDMDHN
VRRQEDGTQPQVNGQ
LHPRAVSTSFGCRVN
NIAVILLTDLIIDHS
DYLRWVQTLSEQVQE
CHALSGSTAELREDL
ESWLVPATLPGPGES
HFHIRCFTCQVCGCG
QIQFSQCTDRIKDLE
NKPVSFLTAQQLQQL
WGWRIERTREGDSMA
PPPAPRPTSRYNSSS
WSWSEQNTNEQQPDV
NLGKKPGTITTVGLL
LEVIYGDTDSIMINT
ARLMSALTQLKERYS
TINIIICTVDYLLRL
QKREAEETAQKMAQL
PEKAKSPTKEEAKSP
DYESYEKTTRTSDVG
SGNIKNWTSLMAAVA
PPARLAKTPSSSSSQ
VDSSQKPTPSAREDP
MLCEEFVTSYKALKS
LVLEIEGTNVSTTYI
SQLKSDLTSRDDLIS
QTKVVPQTVMATVPV
EDSGGGGTGVKKKRK
DQIIPRMTTQSPLNQ
KEIDNDFTNETKISP
AENGKEGTAKEGAPG
PRPLPRSTFARPAFN
YSLLVLATDLGSPPQ
XXXMFKNTFQSGFLS
EERCSFATVNIQLKG
TPLGPGSTSSLERRK
PSSLKGSTSLLVHPV
GYPNRDSTKYAEIYG
KVDQHEWTKCNTTPT
SNQPASVTDYQNVSF

KFPGPSHTESYSWPP
PYLSEVLTFCLEVAR
CQDGSNLTASQRAYA
KNLRSISTSNQPRKK
KDVNERNTVKGFRKF
NCQDKLETSLHVLNQ
RSAELGCTVDEVESL
HMHQNVPTEEQRTNK
KKQSKKGTEEKKKPS
MPDPVDPTTVTKTFK
IASNSELTPSESLAT
RLQMFNVTPHHHQHS
RPDIKKATQAAKQAS
SKKLKKATGASKKSV
APKKPTSTKKPKTMP
ELKHHHVTLEASQKE
GWLLEEQTRKKQKPL
EVIYDAKTVNAVVEF
SECGKGFTMKRYLIA
IRLPRKATKTKKNYK
TPVPRVRTDYVVRPS
PVGKLTQTSGKTTQT
QARPGEGTAPLPPPG
TPLQPQATTLPAQTL
KHVSRQDTLQQCQAW
QNEFLGCTDRLVITP
SLTQAAFTPIPVDTS
KDPRQQSTPQAANSP
KREKEQVTEPAAKVP
EKNQALNTDNYGHDL
PESIPLGTEDRISAS
QHKAVLLTGEQGTAK
DWVDGEPTEAEKDFW
IWIFAHVTQGQDPWI
YLCNAQITMLQMLAL
KTDMLMITRRGVLFV
EELGSAGTSARCPPQ
LEARLWETEKICQLE
TDSRTSLTIENANRN
GPSGSGKTTVITILM
IREIQKVTKLDRDPA
DSLEEKYTEPVILNL
LWAALLVTFLAGCQA
FYYYWKKTPEAASSR
PYRDSKMTRILQDSL
VGEISVNTSVAFLPY
VVVGEIVTEEHLRRA
EFRIDSVTGAITVAK
RGASFGSTSVSGSEP
TTNKQTSTDGKEKTT
VEHLEDQTVTEFDDA
EGSDIESTEPQKQCS
QAPPPQPTQPLQPKA
SKSSQIGTGDLKILT
NSQLARHTSPSVISD
DRSQGGETLDECGLK
YYGLSGMTKNTFEVF
ENAEDDKTENQIPQR
XXMEDSSTDTEKEEE
SEAINELTVLVLVNT

MSLYASLTSEKVQSL
KAFCRMVTRKLETQK
QGEVEEQTFKEKELD
LDDNKLLTLPNGERL
EGYYEAITAVELKSR
SLHQEFSTKQKLLQN
VRDPVRDTPGTCHVT
KVEHVKVTVLDVPGP
KESDAMSTQDQHVLF
DSSNCSITREHICVH
NLVNKDSTRRDSGAY
TVKVTEATITGLIQG
TIVPHGYTEVANIPV
KTADHFWTDPSADEL
ALGEKPITFYRQALL
NSSNKSYTLNGTKTW
DVSGILATQVYTWVD
NLDLDNETFHIYQPQ
IKRKQQDTPGSPDHR
RHRINPETDLPRKLS
GKDQEERTKAIEAFR
ELYEHWKTLMNRQDQ
VKGAAGRTDLLRLRR
LVDIQGLTFQVICSY
FNYLKDATEVKVIIE
DVSRDGMTLTWYPPE
PNLSCKRTLSFQLLL
AVKGAPETLHSMFSQ
PSGADSKTVKLKSPV
RILRDVHTALSTPGS
PPALQAPTGVTPAPS
AIDGGKNTSGLPAEP
LDPECKCTDDRLWEA
SHREKPGTFYQQELP
IKRQVVETMGLFHDM
HPPLQPLTGSAGQPS
MHFESFITEKKQNIQ
PHIKPPPTTPIPQLP
ELAWHVKTITITEME
GNPRVLDTSRSSISI
DNEGISSTVITFMTS
KVEQAVETEPEPELR
RSQKSLWTIKSMSED
KKLKHRRTSETSISP
LSPVLPFTPAAMTQA
SSIYKEDTVLHLCLR
QEIVQKRTFTKWINS
TPPQPSLTASPSSRP
LEDEGEYTCMASNDY
FVIDGKKTTCKLKMK
SALAEGSTYVPYRDS
DGSRCFFTKSKTDYN
AAETARRTREFRSPP
KSLQWGITSPLLRCD
PPFSPHPTLLSSTPK
TFDIEAVTLGTTSNN
AEEFNDETNVRGRLF
FHAVAWGTPGFLTVM
VYLGRDKTMQLLEEL
ENLNKLMTNLKSTAP

PFQMDPETGLITVRE
QREIERNTCKIKLFC
TCIHGVWTQLPQCVA
YLDFSVSTTGMLAGV
IPEPSNITGNSITLT
NGVSVEDTINLLASL
TSSAQKITKSVKAPT
GSQFFITTKPAPHLD
PTDEVPSTPLALEPS
SEADSSETSVSEKEN
ERCIKELTYQAEEDK
IYGKPIPTIQWIKGD
YDGGSPVTGYWLEMK
SYSENIATLQFSRVE
HSKKVLNTGHPLVTS
VRPARRRTPSPDYDF
INIEHALTFLRNRSI
AVSSFKKTSEMEASS
SGSQMGSTLSLAERP
DGERDPFTCPGFASQ
HGNNQDLTRNSSTPG
YYALGQATGLDEEKR
NIVHLSKTHFPIVQN
LRRIANVTLIDLRGC
EYQCSSSTPSLNLTN
VAGQIRATQQQHDFT
SSVHAQSTLESVISQ
ESVSSPPTLHMKTGL
ESCGSNSTNRSTAES
NEKVEEFTTFVKALA
VKWFKNGTEILKSKK
LACKVTGTPPIKITW
YWVMAFITKTIKLVK
KKISAAQTRLSELHD
ILRQLRQTVEATNSM
LIRSASVTPKINQLC
PSVICEHTKQFQTIS
NKQQNFNTGIKDFDF
PYPSEASTTFPESHL
DMAPLDHTSDKSLLD
QKIERVSTKVIRTLK
GHPAPPVTSARFPSW
ILNSLGLTISVSPSD
GAEIEIRTYDLKANA
RKAAASATVSVQVTD
VLGQLGITKVFSNGA
VSMHKFLTIPRLEEL
LELYLVKTSDSGTYT
KKDGFNITTSEKCNI
VPPSSHETGFIQYLD
ASDKYRMTFIDSVAV
VDPSKFMTPYIAHSQ
GSAEGGPTELTPATS
VQGNPGKTKKGELSI
EIEERARTEIISTDN
QLRDVQETVGLPVVF
VPHILRSTLPATALP
DLDIIQVTVPDPSPT
VDASFMQTLWNMNPI
FPLPDEKTKKRIFQI

ENALQLGTPVLIENI
LLAILLKTTNRASDV
INNVRRMTSHPTLPY
EKNFSFLTKGANQHN
KQSQEEETHRLEIEE
PAAIAAATTTLAQEE
LEGKMKGTKVKTPEM
ARYSIENTDSSSLLT
GWGSPQATAMVLNNL
FGGLDVLTISLGGIP
EEGLRAWTEGEKQQV
KAEKSSSTDQKDSKP
YSLWEHSTKNHLQLE
THEASKLTNHVELFK
SLFVRDATRDHRGVY
XXXXXXMTTQAPTFT
DAYLVLHTAKTSRGF
YQVGKEFTSKMDLEK
KKEQCSSTQSKSNKT
QINKRYKTKDDIKST
REKVASDTEEADRTS
LGHLAKFTCEIQSAP
HVVFGSETGEGENND
TAITVSDTVAVKETS
ITLPVASTASTSLVV
ELNRKIATQEVLIRH
YQLSKEYTLDVYRLS
SLGGNAKTAIIANVH
MIKVRQYTVKEIREG
EFVSHPSTTCREQMY
DSGEKRVTITEKSVA
SFLSSNSTFCNKQNV
DCKKLIDTFGIRWPE
PNRTGARTLADIKAK
VQQVHEETANTIQRK
KVAERKETEFFSSLP
WAVDRCYTGSGRVAA
KKQKSIFTSVPDTPD
EARNDAGTASCSIEL
EMKKSVHTQVNTNTT
QELYLFDTTGKHLYT
DQLNEQVTKLQQQLK
ARLREVQTGWEDLRA
TIKAIKDTEAQKVST
NFKKENETLKSDLNN
DVNVIEGTKAVLECK
SCPSEPNTAALQAAL
VLPRVRFTRHGKELK
AASRPDSTHMVEARQ
KSDVPPLTTKGKKAT
AAQLKPMTPFKRTRI
KINREKATFKHPRTT
HCISLKDTNKRVESS
SPSADIGTTTEGDSI
PSRKSVLTDPAKLKK
FSVSICQTLMQDQQT
AEELSYKTAHEDISQ
LFVIDPNTGVITTQG
LDLGSLNTSSHRTVP
WVNEKMKTATDEAYK

ISVSPLATSALNPTF
LSVSVVQTPTTFQET
CSELSGNTKSRESIQ
VIFYRDKTEELMELT
SCGKKEYTIVVKVLD
IDQFLIATFMQLRLI
QQQAFPVTEIDCAQD
VKGGVDVTLPRVEGK
NSSPDEDTEVKTLKL
MATFECETSEPFVKV
FYLNKMKTFEEPPFE
KIPSRFSTPEFTILN
LYHSEAFTVNFGDTE
AGCPVGQTRTNTFEQ
VYWDYAMTIRLEEIV
PLKRLLNTWTNRYPD
SCQRDPNTGRLNGES
LILEGTFTGTPPISV
LQPTAGGTSKNEGTI
EAISLSETVKKVEYD
RRGRKPKTVLNGILS
RNPEGSRTLSPSRGK
VHERDVNTDFLLILL
KQRKDKDTLKAEEKK
ELPLIFITPLSDVKV
PKPAVAHTAEREQVI
SSGNCLHTDGNETNV
PPDRFKFTSVTDSLE
GTTVVKVTPDLKPTE
GIGPPVETIQRTTAR
RMYLSCLTTSRSMTD
RARHRCQTPHLLAAX
IEYLMGGTYRCTYTR
QPRPPPSTQAPSVNG
MIKLKRGTKGLVRLF
AGKASCTTHLYIKAP
SMALYNRTRRISQTS
DIKWVDDTHALGVFS
VLSNVEVTLNVLADS
RVFKIKSTVVVKDLV
RELEIARTNQEHAAL
EDISPVQTDALDSVR
LCLEPPNTHRVSSFL
XXXXMVLTLGESWPV
SLGVILYTLVSGSLP
VVVISGDTGCGKTTR
RVCSKRGTTESKEVL
DSEDESETIPTDKTV
YGADHPYTARELEAL
TSLHCALTEGLEILS
LGGNSYVTWNYAWSK
PRVRKPKTTPTPRKM
QYHRGEMTALREELK
NMVQRPSTVDFIDWV
NIEECSLTQQNKSTV
ILEILESTIEDYAQY
SEDDNFGTAQSNKAI
HQNVVAETLSALENK
ESDSGDYTCIATNMA
WKYYCLPTGWANFGV

EYDPSKATFVKVVPT
IYRGEELTYDYKFPI
PGEKRKMTDSHVLEE
ARAYPELTLAIFSEI
KCIDPGKTLPEALDY
KQLLEYQTMLKGRCP
RLHQLHITGKLPAGS
GDLPEAGTTRTFSVV
LNPAVLSTASLGSSQ
MTAFADRTVVTIAHR
DDQELQITDRIKIQK
ILGAKEGTQEFVHTE
DGEWVYNTFCIYKRC
AGAPPPCTAVTSDHL
ELPRHPPTFDNLYEY
QNYHLECTETQAWMR
PAIFGVLTKELLQLF
RGISKHSTSGTDEGE
LLHQGLSTSHFAWAF
KVDMKDVTKINREKA
AVQLNIFTAVLSALK
DTTKVSFTYDETAGM
KAVLQRKTACQDTDE
TVQSKILTTGENYKT
RRNCCYKTNSHGETV
PDCNFRVTDVIEGTE
RRGRAGATSGELPVV
GKQPFLGTLQDNLIE
KDAASFLTRMKEKHG
CGNFDLKTINEMRTP
KPDDKAKTLSYEPLS
SYWSIFMTLLHFVAS
GDGPRSETKEFTTPE
SRESKMQTRILKQDL
CYIQHVDTGLWLTYQ
YIIEKRETSRLAWAL
VSEPELATLSGDMAQ
AYDPEDETILEEAKV
SSKRRPRTRAQKVEV
SPDSPSSTPTPSPVS
LNESTFDTQITKKMG
EVRKSALTLVMGPLD
VYYSRSKTTHIIATN
YVIVPLKTGLQEVEV
VGIDLGSTGDTTEAL
ALAVVDVTKRHVDLK
ERKQERETFSSAEGT
APKAPEVTTVTKDSM
HYLHLRATVVSIQRR
TKDKKIITNHQKKNL
SDLDSNQTHSSGTVT
PFKAAPHTIHPPCVV
EGNDIRKTNVAQIRM
SPFSSPETSRFGHHI
IQRAAESTEEVSSLR
ANGEIAYTIAEGNTG
YRKHYEDTKANVHIP
GMMSDYGTARRTGTP
MAHMFVSTNLGESFM
QKICGKKTAFDPSVD

GQDYEHVTMLRDKFR
RMEIKDQTKCAASVG
EISSASRTCTFLPPF
RDGQVISTSTLPGVQ
KRFDFDYTTMHRIKL
DLKSLPDTELMKDTA
EQNMIGMTPTVIAVH
PKDPVIETIRNYFPE
QSLPASQTHMRVTAS
AVALVFLTGCQAWEF
DTDENVGTPCPVSAF
HSNMQFSTFDRDADQ
GFSWIEVTILKPHLQ
KLKPDPNTLCDEFKA
HHLGKEFTPSAQAAF
YILIGSSTSYCEIQG
LGANGRHTSSWLVTP
GRIGRLVTRAAFNSG
DSRFGSLTSNFKEFG
PENLPPLTADFAEDK
LKSNSMVTLGCLVKG
FPESWLWTIEELKEP
EPKNLVKTNCELYEK
KAQEAGYTNATTTDQ
GREEEFFTYRSSVTY
SMILDICTRYLGDVD
VKDSCVGTLVVKGDP
YRLVGEATISCWYSQ
YVVLARLTAQPAPSP
DEANQDFTNRINKLK
QDIIWDDTKQTVRVL
CQNDIMLTATCKSDG
LAKKYEATLEKCCAE
NLLDNWDTLGSTVGR
SLKYNFVTPLTHMVV
DPPACYGTVLAEFQP
